# Genome-wide identification and expression analysis of calcium‑dependent protein kinase and its related kinase gene families in melon (*Cucumis melo* L.)

**DOI:** 10.1371/journal.pone.0176352

**Published:** 2017-04-24

**Authors:** Haifei Zhang, Chunhua Wei, Xiaozhen Yang, Hejie Chen, Yongchao Yang, Yanling Mo, Hao Li, Yong Zhang, Jianxiang Ma, Jianqiang Yang, Xian Zhang

**Affiliations:** 1 Department of Horticulture, Northwest A&F University, Yangling, China; 2 Wenshan Academy of Agricultural Sciences, Wenshan, China; National Taiwan University, TAIWAN

## Abstract

The calcium-dependent protein kinase (CDPK) is a ser/thr protein kinase that plays vital roles in plant growth, development, and responses to multiple stresses. Despite an important member of the stress responsive gene family, little is known about the evolutionary history and expression patterns of *CDPK* genes in melon. Herein, a total of 18 *CDPK* genes and 7 CDPK-related protein kinases (*CRK*) genes were identified in the melon genome via bioinformatic analysis, which were unevenly distributed across eleven chromosomes with an apparent exception for chromosome 3. Comparative syntenic analysis between *Cucumis melo* L. and *Arabidopsis thaliana* revealed that 13 *CmCDPKs* and 19 *AtCPKs* existed in 20 corresponding syntenic blocks. In addition, based on gene structure and phylogenetic analyses, all *CmCDPKs* were divided into four groups (CDPK I-IV) and *CmCRKs* clustered into one group (CRK I). Interestingly, group CDPK IV was clearly distinct from the other three CDPK groups, but clustered with CRK I on the phylogenetic tree, implying their origination from a common ancestor. Furthermore, *CmCDPK*and *CmCRK* genes were differentially expressed in response to various stimuli, such as biotic stress (*Podosphaera xanthii*), abiotic stress (salt and cold), and hormone (abscisic acid) treatment. To our knowledge, this is the first report on *CDPK* and *CRK* gene families in melon, which provides a basic foundation for functional characterizations of *CmCDPK* and *CmCRK* genes in the future.

## Introduction

To survive frequently occurring environmental stresses during plant growth process, plants have evolved an effective defense mechanism comprised of sophisticated signal transduction pathways. Calcium (Ca^2+^), a universal second messenger, plays an important role in plant growth, development, and responses to various environmental stimuli [1–3]. During exposure of plants to stress, transient changes in Ca^2+^ concentrations in the cytoplasm can be sensed and decoded by an array of specific Ca^2+^ sensors and Ca^2+^-binding proteins, such as calmodulins (CaM), calmodulin-like proteins (CaML), calcineurin B-like proteins (CBL), and the calcium-dependent protein kinase (CDPK) [4–6]. CaM, CaML, and CBL serve as Ca^2+^ sensors that can combine and interact with target proteins to transfer signals to downstream pathways. However, CDPKs feature the unique activity of both Ca^2+^ sensing and response within a single protein that can directly translate Ca^2+^ signals into downstream phosphorylation signals. Thus, only CDPKs can function both as Ca^2+^ sensors and effectors [5,7–9].

Calcium-dependent protein kinases (CDPKs) are ser/thr protein kinases, also named CPKs, which have been identified throughout the plant kingdom as well as in several protozoa, but are absent in animals [10–12]. In different plant species, CDPKs have been identified and characterized via four typical domains, including a variable N-terminal domain (consisting of the myristoylation and palmitoylation sites), a catalytic ser/thr protein kinase domain, an auto-inhibitory domain (acting as a pseudosubstrate combined with kinase domain to inhibit activity), and a C-terminal regulatory calmodulin-like domain, containing EF-hand motifs for Ca^2+^ binding [13–15]. CDPK-related kinases (CRKs) are another type of protein kinase that share similar domain structures with CDPK, such as the ser/thr kinase domain. However, CRKs belong to an autocephalous gene family due to a lack of EF-hand domains [16,17].

To date, genome-wide analyses have identified 34 *CDPK* genes in Arabidopsis [13], 31 *CDPK* genes in rice [18,19], 20 *CDPK* genes in wheat [20], 30 *CDPK* genes in poplar [21], and 41 *CDPK* genes in cotton [22]. In recent years, an increasing number of *CDPK* genes have been identified in horticultural plants, such as 19 *CDPK* genes in cucumber [23], 29 *CDPK* genes in tomato [16] and 31 *CDPK* genes in pepper [17]. Compared to extensive studies of *CDPK* genes, only a few studies have been focused on the identification of *CRK* genes. By far, genome-wide analyses have identified eight *CRKs* in Arabidopsis [14], five *CRKs* in rice [19], nine *CRKs* in poplar [21], six *CRKs* in tomato [16] and five *CRKs* in pepper [17]. Nevertheless, our knowledge of *CDPK* and *CRK* gene families for many other economically important horticultural crops, such as the melon (*Cucumis melo* L.), still remains scanty.

*CDPK* genes are ubiquitously expressed in plant different organs, such as roots, stems, flowers, fruits and seeds [18,20,23,24], and their localizations have been validated in the plasma membrane, cytoplasm, nucleus, chromatin, cytoskeleton, chloroplast and mitochondrion [25–29]. Numerous studies have confirmed that *CDPKs* are involved not only in plant growth and development, but also in abiotic and biotic stress responses. In Arabidopsis, *AtCPK24* plays an important role in pollen development by connecting pathways between the vegetative nucleus and generative cells [12]. *OsCPK8*, *OsCPK17* and *OsCPK28* are almost undetectable in the panicles of rice, but are highly abundant in vegetative tissues [30]. In sandalwood, *CDPKs* are transiently expressed in the endosperm, but are highly activated and accumulated during periods of sprouting and fruit ripening [31], indicating that *CDPKs* participate in various processes of plant growth and development. In recent years, another pivotal role of *CDPKs* has been confirmed in the response of plants to biotic stresses. Some grapevine *CDPK* genes, *VpCDPK6*, *VpCDPK9*, *VpCDPK14*, *VpCDPK16*, and *VpCDPK19* play positive roles to powdery mildew pathogen caused by *Erysiphe necator* [32]. Expression of gene *TaCPK2* can be induced by powdery mildew in wheat [20], and *CaCDPK10* plays a negative role to *Ralstonia solanacearum* in *Capsicum annuum* [17]. *CDPKs* are also involved in the response of plants to abiotic stresses. *OsCPK4*, *OsCPK7*, *OsCPK12*, and *OsCPK21* transcripts were reported to be up-regulated in rice in response to salt stress [33–36]. During salt stress, *AtCPK23* is known to play a role in the response to salt stress by controlling K^+^ channels [37]. Furthermore, *OsCPK12* promotes salt tolerance via a reduction in ROS accumulation [35]. During cold stress, low temperature induced expression of *OsCPK13* [38], but *ZmCPK1* was reported to function as a negative regulator in the signaling pathway that responds to cold stimuli [39]. During drought stress, *ZmCPK4* and *OsCPK9* can enhance drought tolerance via stomatal closure [40,41]. *AtCPK4* and *AtCPK11* interact with the ABA transcription factors ABF1 and ABF4 and phosphorylate them to regulate drought tolerance [42]. *AtCPK10* is involved in plant responses to drought stress via the modulation of ABA- and Ca^2+^- regulated stomatal movements [43]. Moreover, *CDPKs* are also reported to participate in the signal transformation of hormone, such as abscisic acid (ABA) [44–46], salicylic acid (SA) [47], jasmonic acid (JA) [48], and gibberellic acid (GA) [38,49].

Despite extensive studies of *CDPK* and *CRK* genes on many other plant species, little is known about these two gene families in melon, an agriculturally and economically important crop around the world. Heavy losses in melon production are frequently caused by various biotic stresses such as powdery mildew, and abiotic stresses including drought, salt and extreme temperatures [50]. The availability of the complete melon genome sequence provides an opportunity to perform a genome-wide analysis of multigene families [51]. In this study, we identified *CDPK* and *CRK* gene families in the melon, and analyzed their genomic structures and chromosomal distributions, as well as their syntenic and phylogenetic relationships. To further elucidate their possible involvement in melon stress responses, transcriptional expression analyses were also performed under biotic (powdery mildew) and abiotic (salinity and low temperature) stresses as well as ABA treatment. Our study provides new insights into the evolutionary history of the *CmCDPK* and *CmCRK* gene families and reveals a set of potential candidate genes for future genetic modification to increase pathogen resistance and stress tolerance in the melon.

## Materials and methods

### Identification of melon *CDPK* and *CRK* genes

The protein sequences for 34 *CDPKs* and 8 *CRKs* of Arabidopsis were obtained from the Arabidopsis Information Resource (http://www.Arabidopsis.org/). To identify the melon *CDPK* and *CRK* gene family, Arabidopsis CDPK and CRK protein sequences were used as queries to search against the melon genome (https://melonomics.net/) using BLASTp with the E-value setting to 1e^-5^. In addition, HMM (Hidden Markov Model) profiles of protein kinase domain (PF00069) and EF-hand_7 domain (PF13499) [32] were downloaded from Pfam database [52] and also exploited for the identification of *CDPK* and *CRK* genes from melon genome using HMMER 3.0 (http://hmmer.janelia.org/) with default parameters. To further verify the reliability of these candidate genes, we also performed BLASTp search at NCBI using full-length amino acid of putative melon *CDPK* and *CRK* genes. Among those genes with alternative splice variants, the longest was chosen for further analysis. All non-redundant putative candidates were verified with the InterProScan program (http://www.ebi.ac.uk/interpro/) to confirm their completeness and presence of the core domain. Subsequently, all candidate gene sequences were future examined via the following online tools: SMART (http://smart.embl-heidelberg.de/) [53], Conserved Domain Database (CDD) (http://www.ncbi.nlm.nih.gov/cdd/), and ScanProsite (http://prosite.expasy.org/scanprosite/) [54]

### Genomic distribution and synteny analysis of *CDPK* and *CRK* genes in melon

Genes were mapped on chromosomes via identification of their chromosomal position, obtained from the melon database (https://melonomics.net/). MapInspect software was used to draw chromosomal distribution of *CmCDPKs and CmCRKs* (http://mapinspect.software.informer.com/1.0/). MCScanX software was utilized to detect the synteny relationship and duplication pattern of *CDPK* and *CRK* genes in the melon genome, as well as the synteny relationship between melon and Arabidopsis [55]. All melon protein sequences were subjected to search against themselves and proteins of Arabidopsis, respectively, using BLASTp with parameters E-value < 1e^-10^, and setting the output format as tabular (-m 8) [56]. The BLASTp tabular file combined with the melon gene and Arabidopsis gene location files served as input for MCScanX to analyze syntenic relationship and duplication types with default settings, visualizing them via Circos (http://circos.ca/).

### Genomic structure and phylogenetic analysis

Genomic DNA sequences of *CmCDPKs* and *CmCRKs* were obtained from the melon database (https://melonomics.net/), and corresponding cDNA sequences were downloaded from PLAZA (http://bioinformatics.psb.ugent.be/plaza/). The exon- intron organization was carried out with the online tool GSDS2.0 (http://gsds.cbi.pku.edu.cn/) [57]

Full-length protein sequences of *CDPK* and *CRK* genes in Arabidopsis, tomato, and rice were obtained from the Arabidopsis Information Resource (TAIR, https://www.Arabidopsis.org/), tomato genome database (http://solgenomics.net/), and rice genome database (TIGR, http://rice.tigr.org), respectively.

Full-length CDPK protein sequences of Arabidopsis (34) [13], tomato (29) [16], rice (29) [19], and melon (18), as well as CRK protein sequences of Arabidopsis (8) [14], tomato (6) [16], rice (5) [19], and melon (7) were aligned via the ClustalX 2.0 program with default settings. Finally, a phylogenetic tree was constructed with the aligned sequences, via MEGA6.0, using the neighbor-joining method and 1000 replicates for bootstrap [58].

### Plant material and stress treatments

The experiment was conducted in a greenhouse at the Northwest A&F University in China. The melon variety “Nantais Oblong”, an inbred line provided by the vegetable research center of Beijing, was used in our experiment. The seeds of melon variety “Nantais Oblong” were cultured in a greenhouse at a constant daily temperature of 25–28°C, a night temperature of 16–20°C, and a relative humidity of 60% to 80%. The roots, stems, leaves, male flowers and tendrils were sampled separately and placed into liquid nitrogen for the tissue specific analysis. When the seedlings reached the three- or four-leaf stage, uniform melon seedlings were selected and used for the future experiments. For biotic stress experiment, powdery mildew (maintained on leaves of *Podosphaera xanthii* race 2F) was sub-cultured onto fresh leaves every fourteen days. Then foliar portions of plants were inoculated using leaf-brushing inoculation at night and incubated in the greenhouse at a constant daily temperature of 25–28°C, a night temperature of 16–20°C, and a relative humidity of 80% to 95%. Plants cultured without inoculation were used as control. The third leaf was harvested at 0 h, 12 h, 24 h, 72 h, and 120 h post inoculation (hpi), immediately frozen in liquid nitrogen, and stored at -80°C.

For the salt stress treatment, melon seedlings at the three-leaf stage were transferred to Hoagland’s nutrient solution for hydroponics pre-culture [59], and upon development into the four-leaf stage, seedlings were treated with 250 mM NaCl, while normal plants, cultured in nutrient solution without NaCl were used as control. Low temperature stress treatment was performed by placing the plants in a growth chamber at 4°C and at 80% humidity, while plants cultured at 25°C and 80% humidity were used as control. For ABA treatment, melon seedlings at the four-leaf stage were sprayed with 100 μM ABA, while plants sprayed with water served as control. Following the respective treatments, the third leaves were harvested at 0 h, 1 h, 3 h, 6 h, 12 h, and 24 h post treatment (hpt), which were then immediately frozen in liquid nitrogen, and stored at −80°C for further analysis.

### RNA isolation and qRT‑PCR

Total RNA from leaves was isolated using the RNASimple Total RNA Kit (TIANGEN, China) following the manufacturer’s instructions. The integrity of RNA samples was measured via 2% agar gel electrophoresis. First-strand cDNA was synthesized via reverse transcription of 1μg total RNA using the FastQuant RT Kit (TIANGEN, China) following the manufacturer’s instructions. Gene-specific primers for each *CmCDPK* and *CmCRK* were designed by Primer Premier 6.0 (S1 Table). The cDNA was diluted to 100 ng/μl for qRT-PCR, which was conducted on a Bio-Rad Real-time PCR system (Foster City, CA, USA) using the SYBR Premix Ex Taq II kit (Vazyme). The reaction mixture was prepared in a total volume of 20 μl containing: 10.0 μl SYBR Green Master mix, 0.4 μl of each forward and reverse primers (10 μM), 0.4 μl Rox Reference Day1, 2.0 μl cDNA and dilute with ddH_2_O to 20 μl. The PCR conditions consisted of pre-denaturing at 95°C for 5 min, followed by 40 cycles of 95°C for 10 s and 60°C for 30 s. To verify the specificity of the amplicons of each primer pair, melting-curve analyses of the products were conducted at the end of each PCR cycle with 95°C for 15 s, 60°C for 60 s, and then a constant increase from 60°C to 95°C with temperature increasing steps of 0.3°C/s. The melon *β-actin* gene (GenBank accession number AY859055) was used as internal control [50]. Each relative expression level was calculated following the 2^-ΔΔCt^ method [60]. SPSS 21.0 software was used for statistical analysis, and the data were depicted as mean value ± SD of three biological replicate with three technical replicates. The significance of the differential expression between treatments and controls was verified via Student’s t-test. The relative expressions were log2 transformed and visualized for heat map using Mev 4.8.1 and the column diagrams were prepared using Origin 9.0 software package.

## Results

### Identification and distribution of *CDPK* and *CRK* genes in melon

After bioinformatic analysis, we obtained a total of 25 non-redundant sequences. After domain prediction of these sequences via InterProScan, SMART, CDD and ScanProsite, a total of 18 *CmCDPKs* (typically containing both STKs_CAMK protein kinase and EF-hand domain), and 7 *CmCRKs* (containing solely a STKs_CAMK kinase domain) were identified in the melon genome, designated as *CmCDPK1* to *CmCDPK18* and *CmCRK1* to *CmCRK7* based on their chromosomal positions, respectively (Table 1).

**Table 1:** Characteristics of *CDPK* and *CRK* gene family in melon.

Gene	ID^a^	Chr	Gene length	No.ofaa^b^	MW (kDa)^b^	pI^b^	EF hand^c^	N-myristoylation^d^	N-Palmitoylation^e^	N-terminal aa^f^
*CmCDPK1*	MELO3C024122	Chr1:9620346..9633306	12961	501	56.25	5.31	4	No	No	MEKPIKAS
*CmCDPK2*	MELO3C015707	Chr1:27325271..27331475	6205	566	63.36	5.59	4	No	Yes	MGNTCRGS
*CmCDPK3*	MELO3C015189	Chr2:5589335..5593855	4521	531	59.8	6.46	4	No	Yes	MGNCCATP
*CmCDPK4*	MELO3C003730	Chr4:3635832..3639934	4103	516	58.64	5.87	4	Yes	Yes	MGLCFTRT
*CmCDPK5*	MELO3C009574	Chr4:29891445..29896126	4682	585	64.97	5.34	4	No	Yes	MGNTCVGP
*CmCDPK6*	MELO3C009565	Chr4:29965064..29968628	3565	661	74.22	5.06	4	No	Yes	MGNNCLRR
*CmCDPK7*	MELO3C009161	Chr4:32669721..32673288	3568	527	59.42	5.86	4	No	Yes	MGNCCRSP
*CmCDPK8*	MELO3C014588	Chr5:1122720..1126606	3887	529	59.56	6.62	4	No	Yes	MGNCCATP
*CmCDPK9*	MELO3C014556	Chr5:1421889..1424594	2706	535	59.81	5.65	4	Yes	Yes	MGNCCSRE
*CmCDPK10*	MELO3C025374	Chr6:29289988..29294658	4671	519	58.65	6.34	4	Yes	Yes	MGICTSKG
*CmCDPK11*	MELO3C016330	Chr7:22108243..22112765	4523	507	56.75	5.31	3	No	Yes	MGNCSGLP
*CmCDPK12*	MELO3C017756	Chr7:24692093..24697309	5217	527	60.03	5.3	4	Yes	Yes	MGSCVSVQ
*CmCDPK13*	MELO3C007388	Chr8:2507696..2511639	3944	552	61.65	6.03	4	Yes	Yes	MGCCSSTQ
*CmCDPK14*	MELO3C007904	Chr8:6156505..6161719	5215	543	61.59	8.75	4	Yes	Yes	MGVCFSAS
*CmCDPK15*	MELO3C026727	Chr8:16955408..16959420	4013	535	60.53	6.38	4	No	Yes	MGNCCVAP
*CmCDPK16*	MELO3C012326	Chr10:1218591..1222869	4279	575	63.95	5.42	4	No	Yes	MGNTCVGP
*CmCDPK17*	MELO3C023209	Chr11:233802..237889	4088	503	56.27	5.07	4	No	No	MSKSSSAA
*CmCDPK18*	MELO3C002215	Chr12:24386696..24391834	5139	546	62.15	6.15	4	Yes	Yes	MGNCNACV
*CmCRK1*	MELO3C018436	Chr1:261445..267908	6464	603	67.22	9.06	0	Yes	Yes	MGICTSKP
*CmCRK2*	MELO3C026598	Chr4:25956480..25961897	5418	572	64	8.51	0	Yes	Yes	MGLCHGKP
*CmCRK3*	MELO3C007546	Chr8:3526437..3531775	5339	604	67.33	8.72	0	Yes	Yes	MGLCVSKP
*CmCRK4*	MELO3C019099	Chr8:11875126..11881306	6181	570	64.05	6.91	0	No	Yes	MGICQAKV
*CmCRK5*	MELO3C005914	Chr9:24018542..24021758	3217	561	63.12	8.9	0	Yes	Yes	MGHYCSKG
*CmCRK6*	MELO3C020930	Chr11:2732657..2737240	4584	622	69.44	9.15	0	Yes	Yes	MGLCNSKP
*CmCRK7*	MELO3C022260	Chr11:29251128..29259710	8583	609	67.76	8.91	0	No	Yes	MGQCYGKT

^a^.The gene name in melon database(https://melonomics.net/).

^b^.The number of aa, MW, pI was predicted using ProtParam (http://web.expasy.org/protparam/).

^c^.The number of EF hands was predicted by ScanProsite tool(http://prosite.expasy.org/scanprosite/).

^d^.The myristoylation site was predicted by Myristoylator program in ExPASy (http://web.expasy.org/myristoylator/).

^e^.The palmitoylation site was predicted by CSS-plam 4.0 (http://csspalm.biocuckoo.org/).

^f^.The first eight amino acids at the N-terminal. The amino acids underlined indicate putative palmitoylation sites.

**Fig 1:**
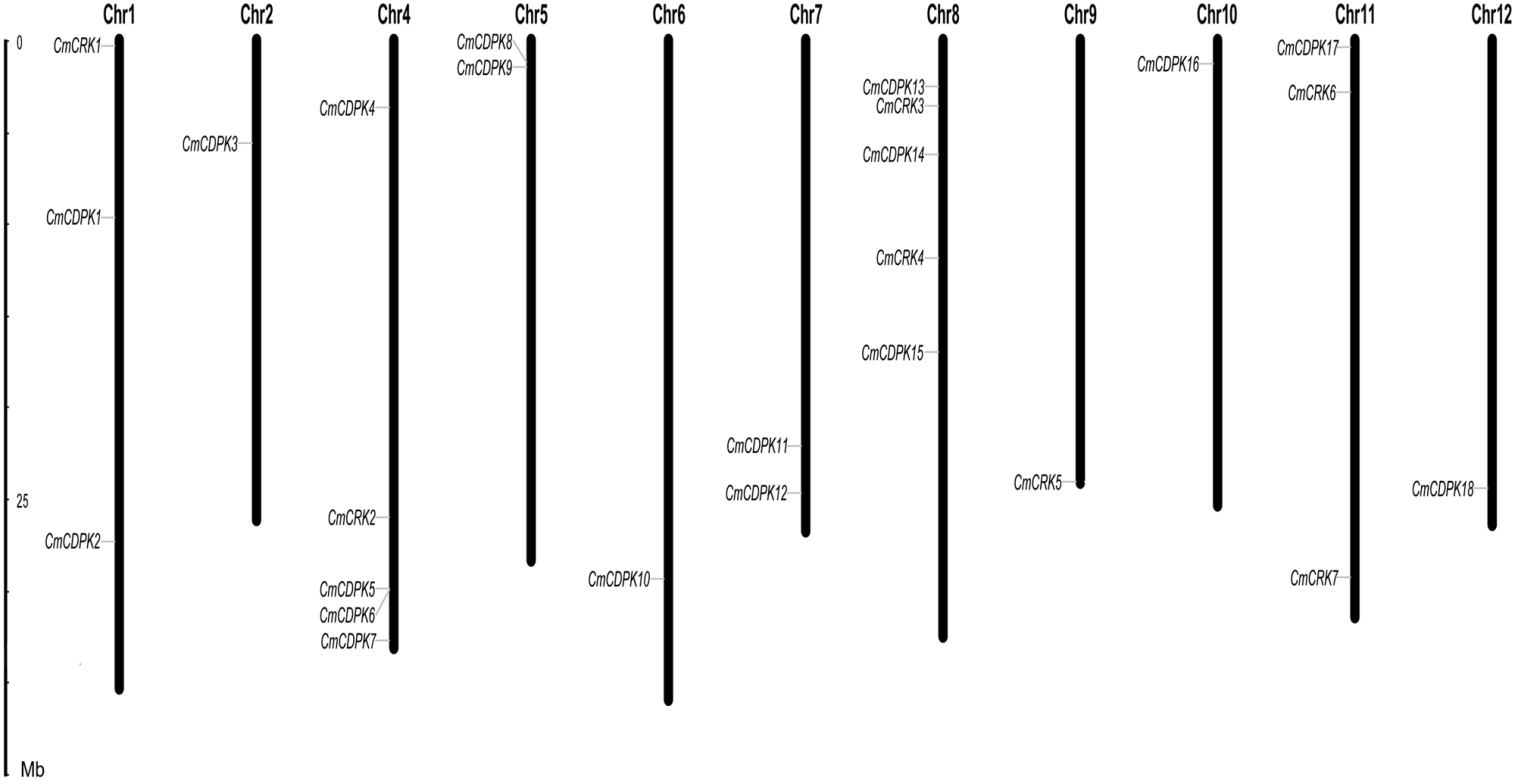
Chromosomal distribution of melon *CDPK* and *CRK* genes.

To understand the genomic distributions of *CmCDPKs* and *CmCRKs*, 25 identified genes (18 *CmCDPKs* and 7 *CmCRKs*) were mapped onto melon chromosomes. As shown in Fig 1, all of these genes were unevenly distributed across 11 melon chromosomes, with only exception for chromosome 3, which contained none of them. The largest number of *CmCDPK* genes (four) was located on chromosome 4, followed by chromosome 8 (three genes) and chromosome 1, 5, and 7 (two genes each). However, among 7 *CmCRK* genes, chromosome 1, 4, 8, 9, and 11 contained 1, 1, 2, 1, and 2 *CmCRKs*, respectively (Fig 1; Table 1).

### Prediction of biochemical characteristics of *CmCDPK* and *CmCRK* genes

Although the genomic sequences of 18 *CmCDPKs* vary widely, from 2705 to 12960 bp, the numbers of predicted amino acid are relatively similar, ranging from 501 to 585 amino acids, with the exception of *CmCDPK6* that encodes 661 amino acids (Table 1). As a result, the molecular weights of the CmCDPKs in our study ranged from 56.25 to 74.22 kDa, with a predicted pI value of less than seven for most of these (except CmCDPK14). The 18 CmCDPKs were confirmed to contain the typical CDPK structure, including an N-variable domain, a protein kinase domain, an autoinhibitory domain, and a CaM-like domain [13]. It is to be noted that the EF hand motif in the CaM-like domain recognizes and binds Ca^2+^ molecules [14,61]. In our study, all the CmCDPKs were predicted to contain four EF-hands, with the exception of CmCDPK11 containing three EF-hands (Table 1). Among the identified 18 CDPKs in melon, 7 CmCDPKs were predicted to contain myristoylation sites, while 16 CmCDPKs were predicted to contain palmitoylation sites (Table 1). Additionally, there were two CmCDPKs, CmCDPK1 and CmCDPK17, which had neither myristoylation nor palmitoylation sites.

In comparison, we only obtained seven CmCRK genes in the melon genome, with predicted protein molecular mass from 63.12 to 69.44 kDa (Table 1). Similar to *CDPK* and *CRK* genes in tomato [16], almost all *CmCRK* genes (except for *CmCRK4*) encode basic proteins, whereas *CmCDPK* genes encode either acidic or neutral proteins. According to the bioinformatic prediction, five CmCRK proteins contained myristoylation sites, while all CmCRK proteins contained palmitoylation sites.

### Gene duplication and synteny analysis of *CmCDPK* and *CmCRK*

**Fig 2:**
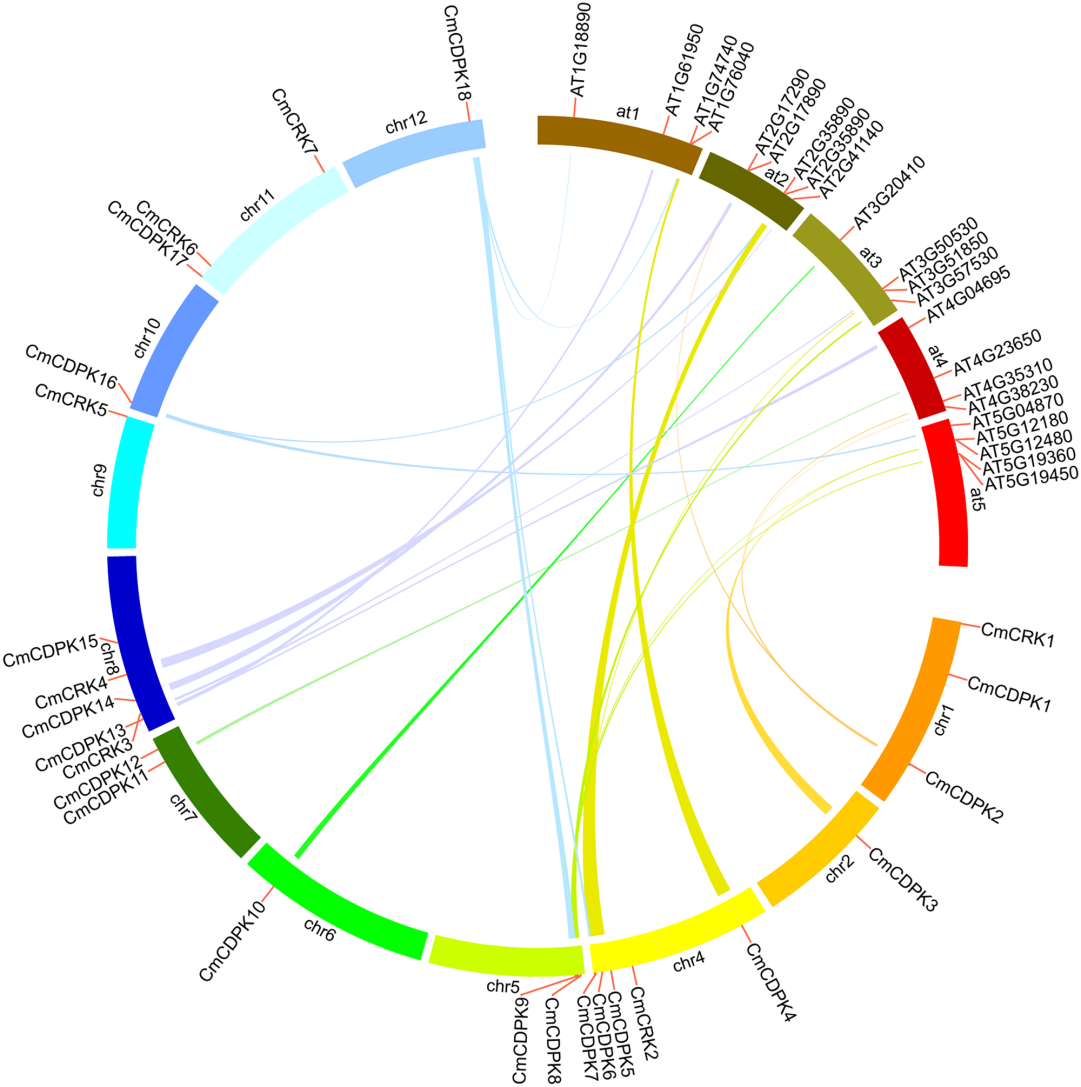
Segmental duplication of melon *CDPK* genes, and synteny analysis of melon and Arabidopsis *CDPK* and *CRK* genes. Chromosomes of melon and Arabidopsis were depicted in different color and circle form. The approximate distribution of each *CmCDPK*, *CmCRK*, *AtCPK*, and *AtCRK* is marked with a short red line on the circle. Coloured curves denote the details of syntenic regions between melon and Arabidopsis *CDPK* and *CRK* genes.

In addition to duplication of the whole genome, tandem duplication and segmental duplication also play important roles in the expansion and function of gene family [62,63]. To investigate possible evolutionary relationships between *CmCDPKs* and *CmCRKs*, we analyzed duplication events in melon using MCScanX software, visualizing the results via Circos. As shown in Fig 2, two pairs of *CmCDPKs* were identified as segmental duplications (*CmCDPK7/ CmCDPK18* and *CmCDPK9/ CmCDPK18*) (S2 Table); however no tandem duplication event existed among *CDPK* genes in melon. Moreover, we neither found segmental duplications nor tandem duplications in CRK genes in melon.

To further explore evolutionary connections between melon *CDPK/CRK* genes and Arabidopsis *CDPK/CRK* genes, a synteny analysis was performed via MCScanX using default parameters. As a result, we found 20 collinear gene pairs in melon *CDPK* and Arabidopsis *CDPK* gene family, including 13 *CmCDPKs* and 19 *AtCPKs* (Fig 2; S2 Table). These results imply that the syntenic relationships could be divided into three types. The first type is that a single *CmCDPK* corresponded to a single Arabidopsis *CDPK* gene, including *CmCDPK3-AT5G12480 (AtCPK7)*, *CmCDPK4-AT1G76040 (AtCPK29)*, *CmCDPK6-AT2G35890 (AtCPK25)*, *CmCDPK7-AT3G51850 (AtCPK13)*, *CmCDPK10-AT3G20410 (AtCPK9)*, *CmCDPK11-AT4G23650 (AtCPK3)*, *and CmCDPK14-AT2G17890 (AtCPK16)*. For the second type, a single *CmCDPK* corresponded to two Arabidopsis *CDPK* genes, including *CmCDPK8*-AT5G19450 *(AtCPK8)/ AT3G57530 (AtCPK32)*, *CmCDPK9- AT5G12180 (AtCPK17)/ AT5G19360 (AtCPK34)*, *CmCDPK13-AT1G61950 (AtCPK19) / AT4G04695 (AtCPK31)*, *CmCDPK16-AT5G04870 (AtCPK1)/ AT2G35890*, *(AtCPK25)*, and *CmCDPK18- AT1G18890 (AtCPK10)/ AT1G74740 (AtCPK30)*. The last type only contained one melon gene *CmCDPK2*, which corresponded to three Arabidopsis *CDPK* genes: *AT4G35310* (*AtCPK5)*, *AT2G35890 (AtCPK6)*, and *AT4G38230* (*AtCPK26*). Moreover, two collinear gene pairs were identified in the melon and Arabidopsis *CRK* gene families including *CmCRK3-AT3G50530 (AtCRK5)* and *CmCRK4- AT2G41140 (AtCRK1)*.

### Phylogenetic analysis and structure of the *CDPK* and *CRK* gene family

**Fig 3:**
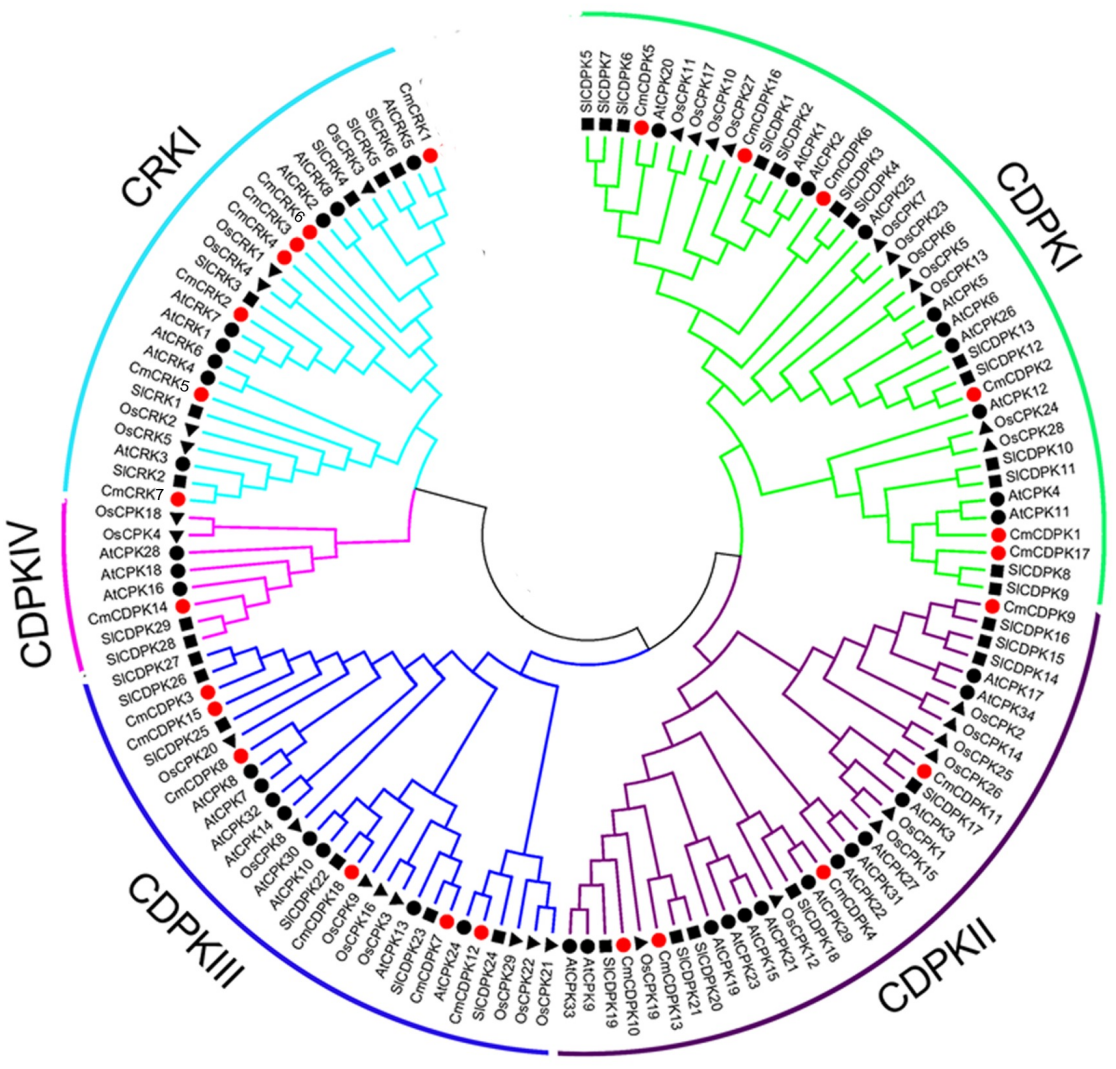
Phylogenetic analysis of *CDPK* and *CRK* genes. Phylogenetic relationships among melon (red circle), Arabidopsis (black circle), tomato (black square) and rice (black triangle). The phylogenetic tree was constructed using the aligned protein sequences by the Neighbor-Joining method with 1,000 bootstrap replicates. Distinct colors lines are used to cluster the subfamily of *CDPK* and *CRK* genes.

To deeply analyze the phylogenetic relationships between *CDPK* and *CRK* genes, CDPK and CRK full-length protein sequences of melon (18 *CDPKs* and 7 *CRKs*), Arabidopsis (34 *CDPKs* and 8 *CRKs*), tomato (29 *CDPKs* and 6 *CRKs*), and rice (29 *CDPKs* and 5 *CRKs*) (S1 Data Sheet) were aligned via Clustal X2.0, and then were used to construct a phylogenetic tree with MEGA6.0 (Fig 3). According to the classification of Arabidopsis *CDPK* gene [13,64], the 110 *CDPK* genes from the four species were divided into four groups (CDPK I, CDPK II, CDPK III and CDPK IV), while all *CRKs* fell into one group CRK I. Interestingly, group CDPK IV evidently clustered with CRK I on the phylogenetic tree. Of the four CDPK groups, CDPK I was the largest, consisting of 6 melon *CDPKs* combined with 10 Arabdopsis *CDPKs*, 13 tomato *CDPKs*, and 11 rice *CDPKs*. CDPK II contained 5 *CDPKs* from melon, 12 from Arabidopsis, 8 from tomato, and 8 from rice, while group CDPK III contained 6, 9, 6, and 8 *CDPKs* from melon, Arabidopsis, tomato, and rice, respectively. Finally, CDPK IV was the smallest group that comprised of one melon *CDPK*, 3 Arabidopsis *CDPKs*, 2 tomato *CDPKs*, and 2 rice *CDPKs* (Fig 3). However, all the 26 *CRK* homologues gathered into group CRK I on the phylogenetic tree. As shown in Fig 3, lots of CDPK and CRK proteins from Arabidopsis, tomato, and rice clustered into sub-branches, while there were no pairs of paralogous relationships among *CmCDPKs* and *CmCRKs*.

**Fig 4:**
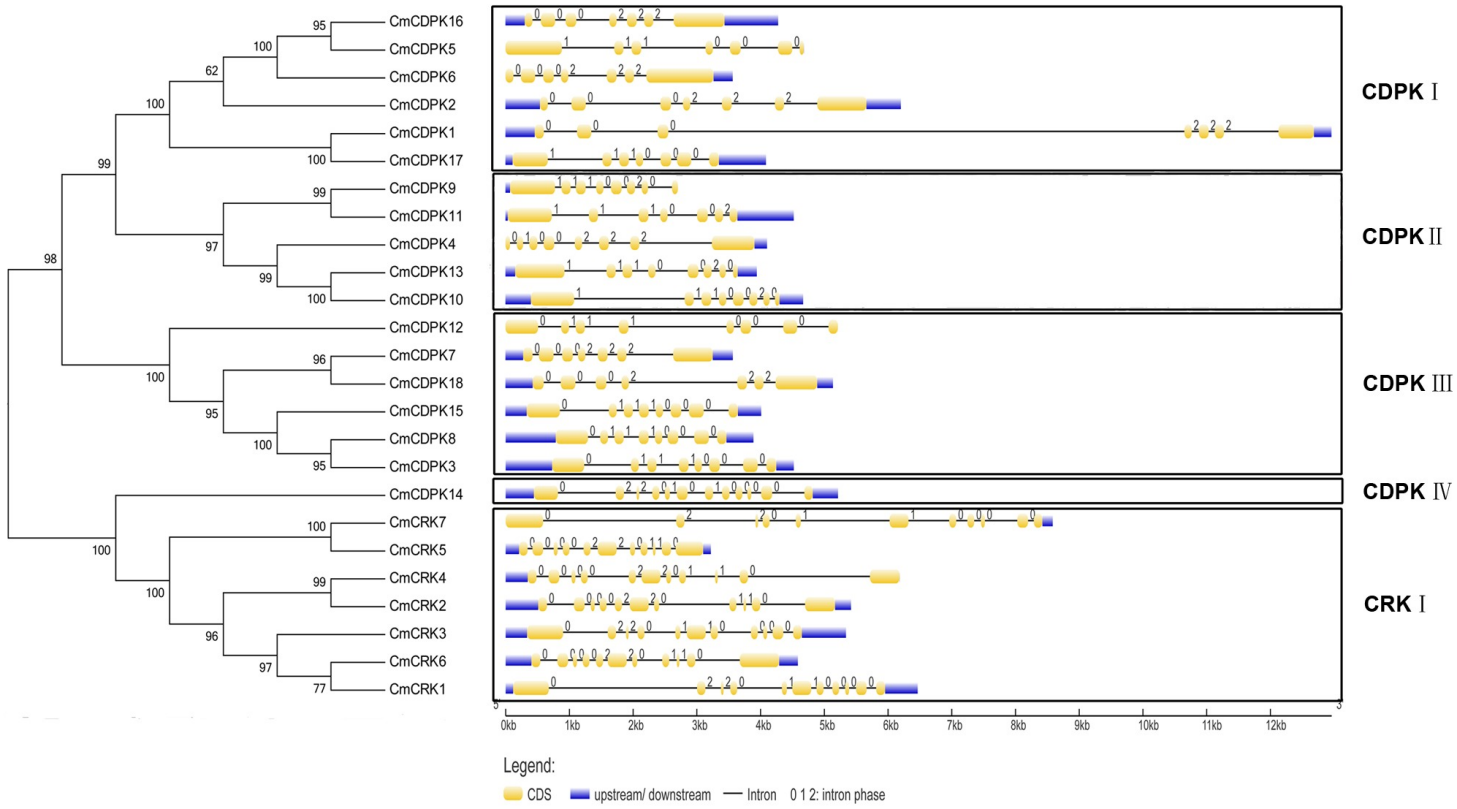
Phylogenetic relationship and exon-intron organization of melon *CDPK* and *CRK* genes. The phylogenetic tree was constructed using the full-length protein sequences of 18 melon *CDPK* genes and 7 melon *CRK* genes by the Neighbor-Joining method with 1,000 bootstrap replicates. Gene structures were performed by online tool GSDS 2.0 (http://gsds.cbi.pku.edu.cn/). Exons and introns are represented by yellow boxes and gray lines, respectively. The intron phase numbers 0, 1 and 2 are labeled at the beginning of each intron. The diagram is drawn to scale.

The exon-intron structures can also provide important evidence to support phylogenetic relationships within a gene family [22]. To get further insights into evolutionary relationships between the CDPK and CRK gene families, gene structures of 18 *CmCDPKs* and 7 *CmCRKs* were integratively depicted, based on the gene annotation profiles and completed melon genome sequences (Fig 4). According to the relatively high bootstrap values, five groups (CDPK I–IV and CRK I) could be observed in Fig 4, which was consistent with the topology of Fig 3. All members of group CDPK I had seven exons, with two distinct intron phase patterns (111000 and 000222), while *CmCDPKs* in CDPK II and III contained seven or eight exons, respectively, sharing similar intron patterns with CDPK I. For example, compared to *CDPKs* in group I, four genes (*CmCDPK3*, *CmCDPK8*, *CmCDPK12*, and *CmCDPK15*) in CDPK III possessed eight exons with an additional intron gain in the 5’ end, exhibiting a phase pattern 0111000, while the intron phase pattern of the remaining two genes (*CmCDPK7* and *CmCDPK18*) in this group was 000222. Group CDPK IV contained only one gene: *CmCDPK14*. Strikingly, *CmCDPK14* carried twelve exons with an intron phase pattern 02201010000, which was significantly above that of *CmCDPKs* (with seven or eight exons) in the other three groups. However, it was similar to *CmCRK1*, *CmCRK3*, and *CmCRK7* of group CRK I, which had eleven exons with phase pattern 0220110000 (Fig 4), inferring that these genes likely derived from a common ancestor. The remaining four members in CRK I carried eleven exons with another intron phase pattern of 0000220110.

### Expression profiles of melon *CDPK* and *CRK* genes in different tissues

**Fig 5:**
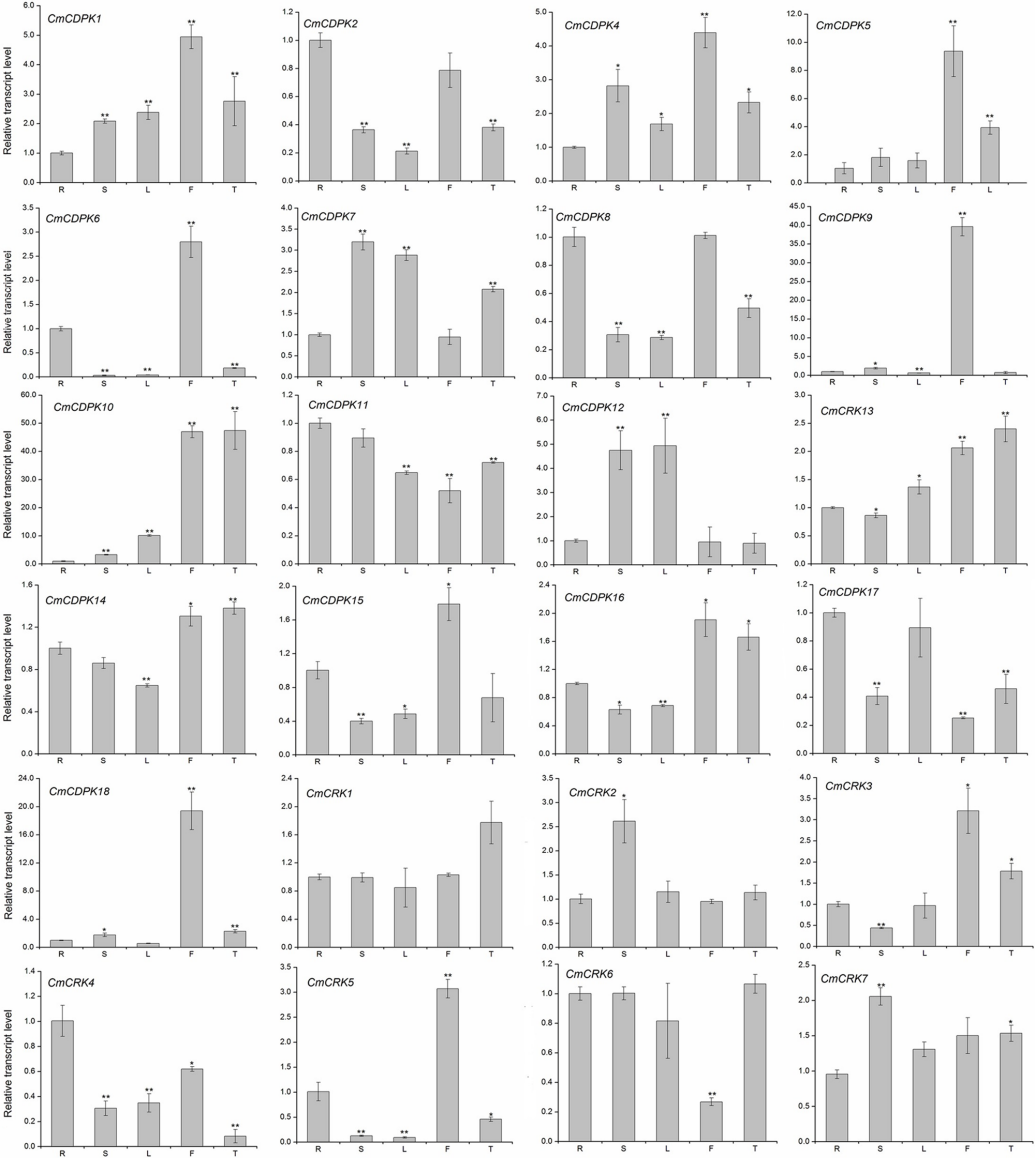
Expression profiles of *CmCDPK* and *CmCRK* genes in different tissues by quantitative RT-PCR. The transcript levels of the respective genes in roots were used as reference and set to a value of 1. R, roots; S, stems; L, leave; F, male flowers; T, tendrils.

To assess the potential functions of *CmCDPK* and *CmCRK* genes during melon development, we investigated the expression patterns of all *CmCDPK* and *CmCRK* genes in five tissues (roots, stems, leave, male flowers and tendrils). As shown in Fig 5, all the identified *CmCDPKs* (except *CmCDPK3*) and *CmCRKs* were expressed in at least one of the five tissues, whereas the expression level of *CmCDPK3* was not detected in any of the five organs. Some *CmCDPKs* and *CmCRKs*, such as *CmCDPK9*, *CmCDPK18*, and *CmCRK5*, were strongly expressed in male flowers, while *CmCRK1* was expressed at nearly the same levels in all tissues (Fig 5). All these data implied that *CmCDPK* and *CmCRK* genes might be involved in the growth and development of different tissues of melon.

### Expression profiles of melon *CDPK* and *CRK* genes under *P*. *xanthii* inoculation

**Fig 6:**
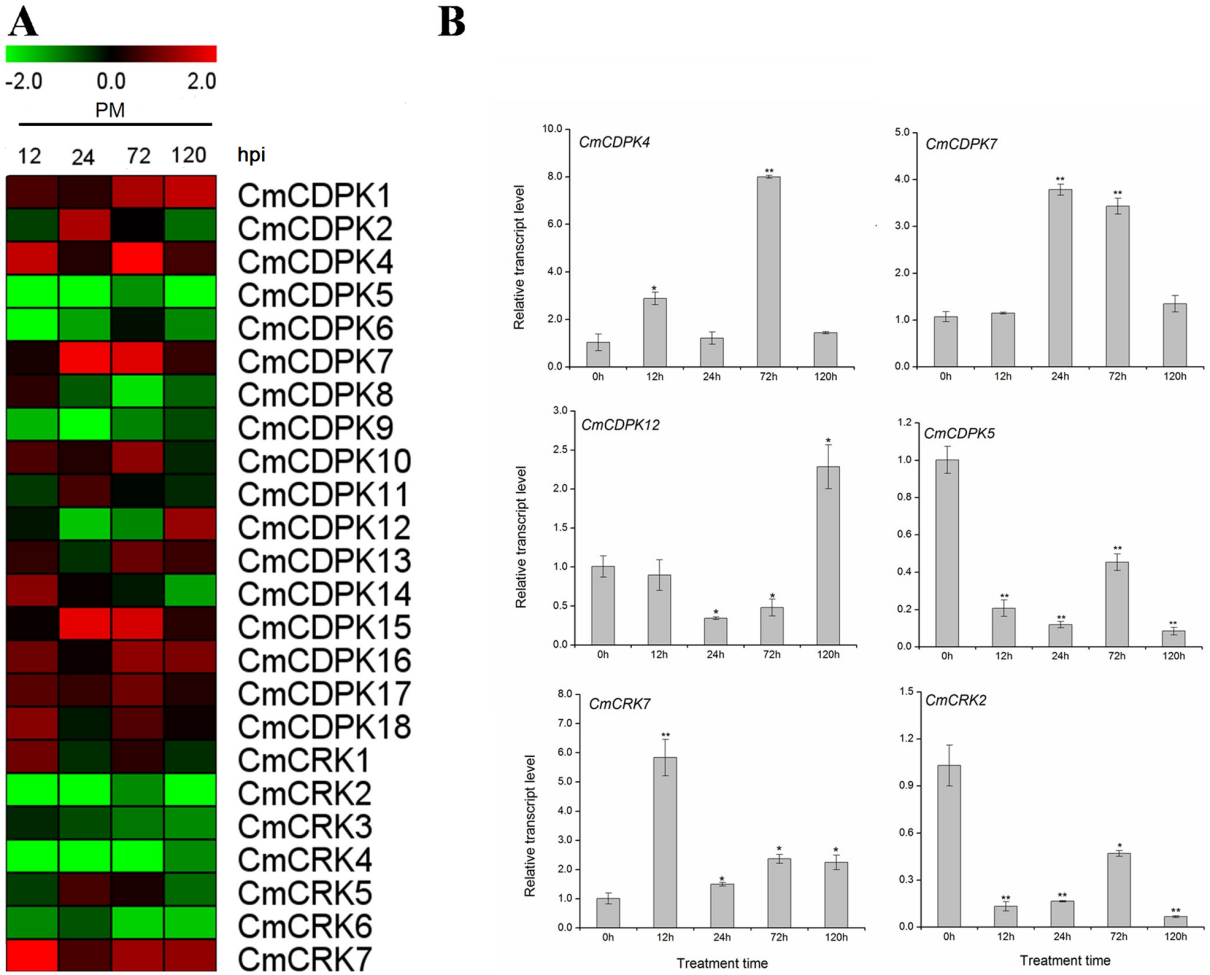
Expression of *CDPK* and *CRK* gene in melon following inoculation with *P*. *xanthii* by quantitative RT-PCR. **(A)** Expression of *CmCDPK* and *CmCRK* following inoculation with *P*. *xanthii*. The relative transcript level was log2 transformed and visualized as heat map by Mev4.8.1. Gene highly or lowly expressed was colored by red or green, respectively. **(B)** Detailed expression of selected *CmCDPKs* and *CmCRKs* with different expression patterns following *P*. *xanthii* inoculation. Means ± standard deviation of three biological replicates each with three technical replicates were plotted as histogram by Origin 9.0. “*,**” significantly differ from the 0 h control at *p*<0.05 and 0.01, respectively, according to the Student’s test.

Powdery mildew is one of the most devastating biotic stresses that decreases melon yield and quality [65]. To get insight into the response of melon *CDPK* and *CRK* genes to powdery mildew, we designed specific primers for all *CmCDPKs* and *CmCRKs*, and examined their expression patterns in melon following inoculation with *P*. *xanthii* race 2F (Fig 6). However, the expression of *CmCDPK3* had not been detected in leaves after using three independent pairs of specific primers. Therefore, it was discarded in further analysis. Results showed that the majority of *CmCDPKs* and *CmCRKs* responded to *P*. *xanthii* inoculation, differing in their expression patterns (Fig 6A). Using fold-changes above two fold or below 0.5-fold as thresholds, 10 *CmCDPK* genes and one *CmCRK* gene were found to be up-regulated upon *P*. *xanthii* inoculation. Among of these, the transcription level of *CmCDPK7* reached a peak of nearly 3.8-fold at 24 h post inoculation (hpi), and rapidly decreased to around 1.3-fold at 120 hpi, similar with the expression pattern of *CmCDPK15*. Moreover, *CmCDPK4* was up-regulated to 2.8-fold at 12 hpi, and then increased rapidly to 8.0-fold at 72 hpi. The only *CmCRK* gene induced by *P*. *xanthii* inoculation was *CmCRK7*, which peaked 5.8-fold at 12 hpi, but then fluctuated around 2.3-fold at 72 hpi (Fig 6B). By contrast, we found a total of ten genes with repressed expression levels following *P*. *xanthii* inoculation. Among them, six were *CmCDPK* genes (*CmCDPK5*, *CmCDPK6*, *CmCDPK8*, *CmCDPK9*, *CmCDPK12*, *and CmCDPK19*) and four were *CmCRKs* (*CmCRK2*, *CmCRK3*, *CmCRK4*, and *CmCRK6*). The transcript abundance of *CmCDPK9* as well as that of *CmCRK4* decreased to around 0.30-fold at 12 hpi, which remained at a significantly lower level than that of control for the rest of the treatment time Intriguingly, *CmCDPK12* was down-regulated at 24 hpi but increased to 2.3-fold at 120 hpi (Fig 6B). All these results imply that *CmCDPK* and *CmCRK* genes likely played important roles in mediating the responses of melon to *P*. *xanthii* induced stress.

### Expression profiles of melon *CDPK* and *CRK* genes under salt, low temperature, and ABA treatment

**Fig 7:**
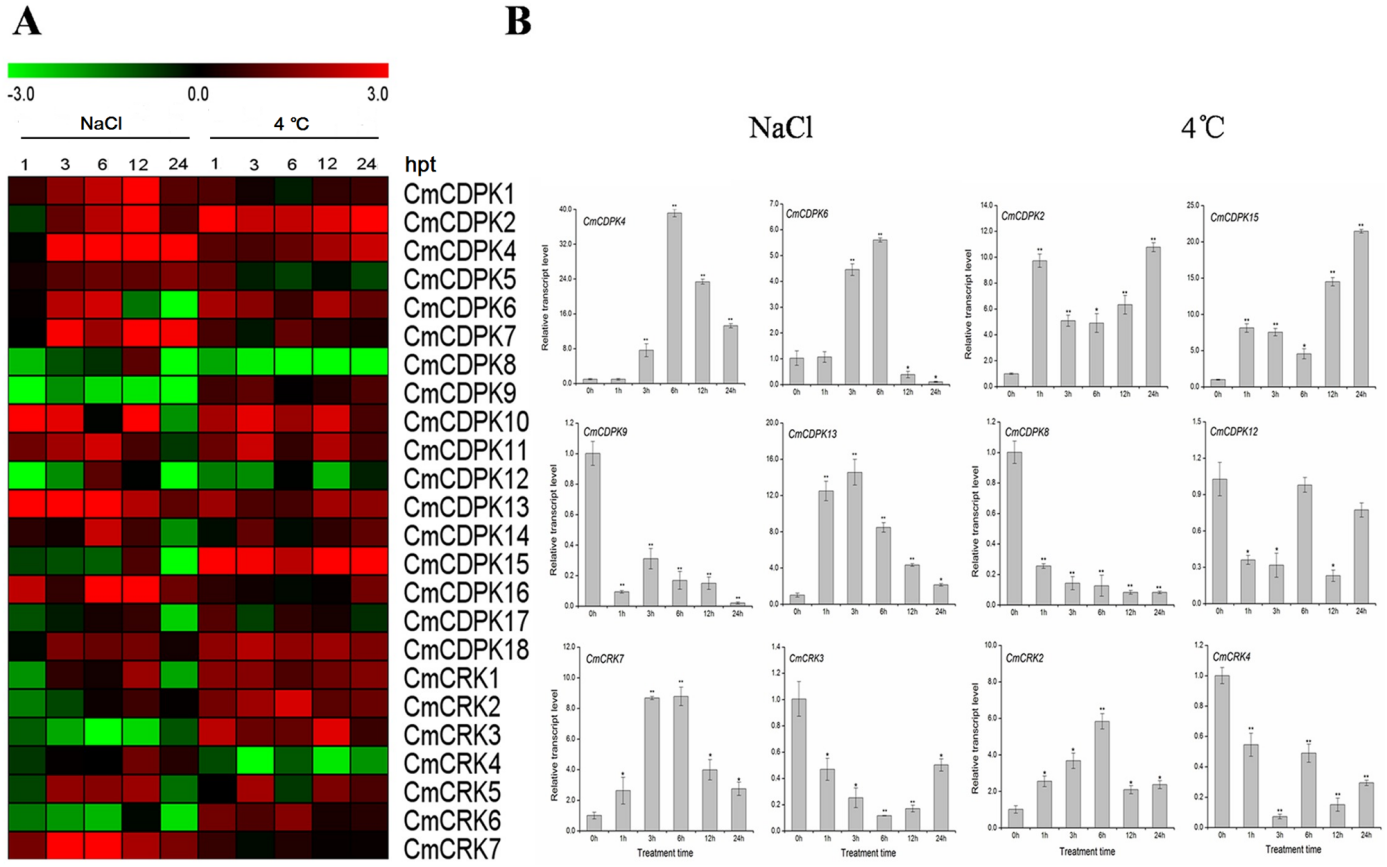
Expression of *CDPK* and *CRK* gene in melon under salt (250 mM NaCl) and cold (4°C) by quantitative RT-PCR. **(A)** Expression of *CmCDPK* and *CmCRK* under salt (250 mM NaCl) and cold (4°C). The relative transcript level was log2 transformed and visualized as heat map by Mev 4.8.1. Gene highly or lowly expressed was colored by red or green, respectively. **(B)** Detailed expression of selected *CmCDPK* and *CmCRK* with different expression patterns under salt (250 mM NaCl) and cold (4°C). Means ± standard deviation of three biological replicates each with three technical replicates were plotted as histogram by Origin 9.0. “*,**” significantly differ from the 0h control at p<0.05 and 0.01, respectively, according to the Student’s test.

**Fig 8:**
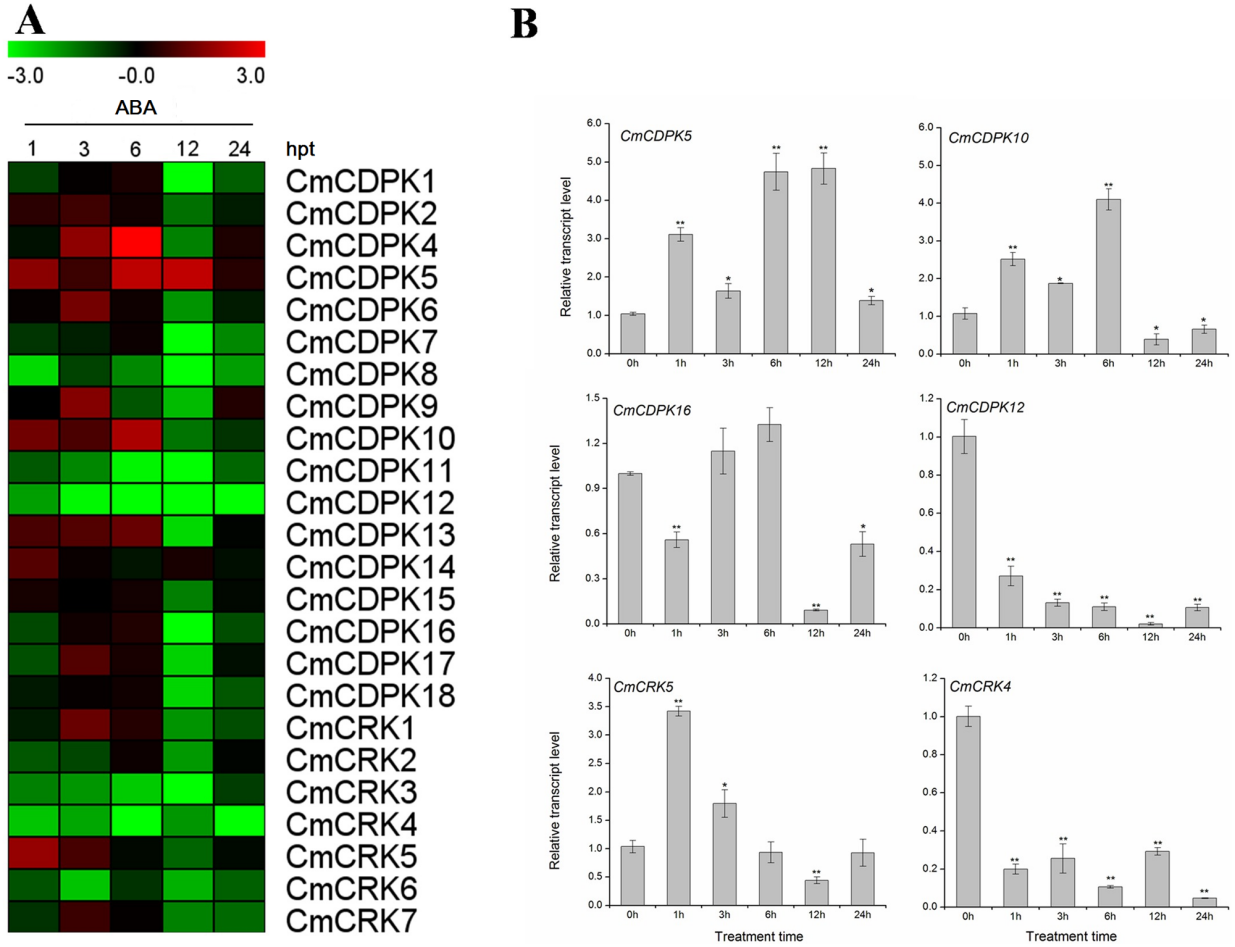
Expression of *CDPK* and *CRK* gene in melon under ABA (100 μM) treatment by quantitative RT-PCR. **(A)** Expression of *CmCDPK* and *CmCRK* under ABA (100 μM) treatment. The relative transcript level was log2 transformed and visualized as heat map by Mev 4.8.1. Gene highly or lowly expressed was colored by red or green, respectively. **(B)** Detailed expression of selected *CmCDPK* and *CmCRK* with different expression patterns under ABA (100 μM) treatment. Means ± standard deviation of three biological replicates each with three technical replicates were plotted as histogram by Origin 9.0. “*,**” significantly differ from the 0h control at p<0.05 and 0.01, respectively, according to the Student’s test.

Previous research showed that *CDPK* genes are widely involved in the adaptation of plants to environment stimuli, and that the expressions of *CDPK* genes are effected by salt, cold and exogenous phytohormones [7]. However, little is known about how melon *CDPK* and *CRK* genes respond to these stimuli. To detect potential functions of melon *CDPK* and *CRK* genes under abiotic stresses and hormone treatment, we investigated the expression of *CmCDPKs* and *CmCRKs* under salt (250 mM NaCl), cold (4°C), and abscisic acid (ABA 100 μM) treatments. As shown in Figs 7 and 8, almost all *CmCDPKs* and *CmCRKs* (except *CmCDPK3* as described above) responded to at least one stress; however some responded only slightly. Compared to the salt and ABA treatment, cold stress showed a stronger and more complex effect on the transcript levels of *CmCDPKs* and *CmCRKs*. Nonetheless, some genes showed similar expression patterns under different treatments. *CmCDPK2*, *CmCDPK4*, *CmCDPK13*, and *CmCDPK18* were up-regulated under both NaCl and cold stresses, while *CmCDPK8* showed lower transcription levels in all treatments. Furthermore, converse expression patterns were also observed, such as the down-regulation of the transcription level of *CmCDPK11* due to ABA treatment, which was up-regulated under NaCl and cold stresses.

Following NaCl treatment, the transcription abundance of *CmCDPK13* sharply increased to 12.5-fold at 1 h post treatment (hpt) and reached to a maximum of 14.6-fold at 3 hpt. However, it decreased gradually for remaining treatment duration, similar to the expression pattern of *CmCRK7* (Fig 7B). Four genes (*CmCDPK1*, *CmCDPK2*, *CmCDPK4*, and *CmCDPK7*) have been found to be strongly up-regulated at 3 hpt and remained at significantly elevated levels (Fig 7A). However, the expression level of *CmCDPK9* dramatically reduced to 0.09-fold at 1 hpt and remained at a stable low level, similar to the expression pattern of *CmCRK3* (Fig 7B). Moreover, *CmCDPK6* showed a much more complex expression pattern, with 1.0-fold at 1 hpt, which increased from 4.5-fold to 5.6-fold at 3 to 6 hpt, then rapidly decreased from 0.39-fold to 0.11-fold at 12 to 24 hpt, respectively (Fig 7B).

The expression profiles of *CmCDPK* and *CmCRK* genes in response to 4°C treatment were shown in Fig 7. Strikingly, only three genes (*CmCRK8*, *CmCRK12*, and *CmCRK4*) were significantly down-regulated due to cold stress. All of other genes were up-regulated, differing at expression levels and treatment times, with the exception for *CmCDPK1* and *CmCRK7* that displayed no obvious changes. The transcript level of *CmCDPK15* rapidly increased to 8.1-fold at 1 hpt, but gradually decreased to 4.6-fold at 6 hpt, which was then up-regulated to a considerable 21.5-fold at 24 hpt, similar to the expression pattern of *CmCDPK2* (Fig 7B). Moreover, cold stress significantly up-regulate *CmCDPK2* at all treatment times, compared to its expression abundance in response to high salinity (Fig 7A). These expression analyses suggest that the majority of *CmCDPKs* and *CmCRKs* were involved in the low temperature response of melon.

As a phytohormone, ABA widely participates in the response of plant to biotic and abiotic stresses [66]. In the current study, we found that all *CmCDPK* and *CmCRK* genes (except for *CmCDPK5* and *CmCDPK14*) were down-regulated at 12 hpt with ABA treatment, such as *CmCDPK16* reaching 0.09-fold at 12 hpt (Fig 8B). The expression levels of *CmCDPK12* and *CmCRK4* were significantly decreased after ABA treatment at all treatment times, while only a few genes, such as *CmCDPK5* were up-regulated by exogenous ABA (Fig 8B). *CmCRK5* in particular responded rapidly to ABA, reaching 3.4-fold at 1 hpt, afterward sharply decreased to 0.44-fold at 12 hpt, similar to the expression trend of *CmCDPK10* (Fig 8B). When compared to NaCl and 4°C treatments, the majority of *CmCDPK* and *CmCRK* genes likely acted as negative regulators in the signal transduction induced by exogenous ABA.

## Discussion

With the completion of the sequencing of the whole genome, an increasing number of studies have focused on the analysis of a particular gene family. Compared to *CRK* genes, the *CDPK* gene family has been investigated in far more plants. However, the relationship between the *CDPK* and *CRK* gene families in melon still remain ambiguous. In the present study, we investigated the physico-chemical characteristics, gene structures, chromosome distributions, synteny relationships, duplication events, and phylogenetic relationships between *CmCDPK* and *CmCRK* genes. Furthermore, we analyzed gene expression patterns in response to biotic and abiotic stresses. Our work provides a better understanding of *CDPK* and *CRK* gene families in melon, which will serve as a molecular genetic basis for the genetic improvement of melon.

### Identification of *CDPK* and *CRK* gene families in melon

To the best of our knowledge, the *CDPK* and *CRK* gene families in melon have never been studied before. In the present study, we identified 18 *CDPK* and 7 *CRK* genes in the melon sequenced genome (Fig 1; Table 1), and all *CmCDPKs* had both STKs_CAMK protein kinase and EF-hand domains, whereas *CmCRKs* had only STKs_CAMK protein kinase [16]. Compared to *CDPK* genes in Arabidopsis (34), tomato (29), rice (31), poplar (30) and cotton (41) [13,16,18,21,22], we found a lower amountof *CDPK* genes in melon. However, this is similar to cucumber (19 *CsCDPKs*) [23], an important vegetable crops from Cucurbitaceae family.

Gene duplication events play a major role in genomic rearrangements and expansions. Whole genome duplication (γ, β, α) events often occur in angiosperms leading to an increasing numbers of whole genome genes [63,67]. Meanwhile, tandem and segmental duplications also play important roles in *CDPK* gene family expansion [68,69]. Two tandem and 12 segmental duplications have been reported in the *CDPK* gene family of poplar [21], 13 segmental duplications in cotton [22], and seven segmental duplications in rice [19], indicating that segmental duplications play a predominant role in *CDPK* gene expansion in a majority of plants. Similarly, the low number of segmental and tandem duplications explains the low *CDPK* gene numbers in cucumber [23]. In our study, only two segmental duplication events were identified among *CmCDPKs* (*CmCDPK7/ CmCDPK18* and *CmCDPK9/ CmCDPK18*) and no tandem duplication was detected (Fig 2; S2 Table), which is a plausible reason for the relatively low number of *CDPKs* in melon. Taken together, it can be postulated that 18 *CmCDPKs* and 7 *CmCRKs* are sufficient for mediating Ca^2+^ signals in melon.

### Structure analysis and evolutionary relationships of *CmCDPKs* and *CmCRKs*

The *CDPK* genes show a high combination with Ca^2+^ via EF-hands, and allosteric properties of Ca^2+^ binding and the activation threshold may cause the differences in number and position of EF hands [70]. Furthermore, the special structure of EF-hand domain can be used to distinguish the *CRK* gene family from the *CDPK* gene family. In general, but not in all cases, the *CDPK* genes in plants have four EF-hands. However, exceptions have been reported for several *CDPK* genes in Arabidopsis and maize that contain two or three EF-hands [13,71]. In our study, all *CmCDPKs* (except for *CmCDPK11*) contain four EF-hands and *CmCRKs* contain no EF-hand domains (Table 1). Hence, it will be interesting to explore differences in the biological function between *CmCDPK11* (three EF-hands) and other *CmCDPKs* (four EF-hands) in the future.

The N-terminus of a subset of CDPK proteins contained a myristoylation motif, which is supposed to promote protein-membrane and protein—protein interactions [72]. Palmitoylation is reversible and provides a regulatory mechanism of the subcellular localization [70,72,73]. Among the identified 18 CmCDPKs in melon, 7 CmCDPKs were predicted to contain myristoylation sites, while 16 CmCDPKs were predicted to contain palmitoylation sites and two CmCDPKs have neither myristoylation nor palmitoylation sites (Table 1). All the seven CmCRK proteins contained palmitoylation sites, and five of them contained myristoylation sites. This is consistent with the previous findings that most CRKs have N-terminal modifications [74].

In our study, we investigated phylogenetic relationships to reveal the evolutionary history of the *CDPK* and *CRK* gene families. Our results revealed that all *CDPK* and *CRK* genes from melon, Arabidopsis, tomato, and rice were distributed among all four groups, which is consistent with an origin of the *CDPK* gene before the divergence of eudicot and monocot [16,32]. Apart from degeneration of EF-hands, *CRKs* showed high similarities with *CDPKs*. *CDPK* genes were identified throughout the whole plant kingdom, even in some ancient plants, such as algae [75]. However *CRK* genes are absent in algae [16]. Previously, it was demonstrated that CRK and CDPK IV originated from a common ancestor, and that they separated after the split of green algae and the last common ancestor of the land plant lineage [16,22]. In agreement with this, a close phylogenetic relationship was found between the CDPK IV and CRK groups in our study (Fig 3). Compared to CDPK and CRK proteins from Arabidopsis, tomato, and rice clustering into sub-branches, there was no pairs of paralogous relationships among CmCDPKs and CmCRKs in phylogentic tree (Fig 3), which may be a possible reason for the number variations of *CDPK/CRK* genes between melon and the other three species.

The exon-intron structure reflects the evolution, expansion, and functional relationships of a gene family [22]. A change in the number of introns reflects the rate of gene divergence. Moreover, the insertion of a small DNA fragment alters the phase, finally leading to variations in gene function [76]. Three main types of mechanisms can cause the differences in the exon-intron structure (exon/ intron gain/ loss, exonization/ pseudoexonization and insertion/ deletion) [32,77]. To get further insights into evolutionary relationships between the *CDPK* and *CRK* gene families, we analyzed the exon-intron structure and intron phases of *CmCDPKs* and *CmCRKs*. As shown in Fig 4, the *CDPKs* were divided into four subgroups, which was consistent with the topology of the phylogenetic tree (Fig 3). The exon numbers of *CmCDPKs* in CDPK I–III were seven or eight. However, gene *CmCDPK14* in group CDPK IV contained 12 exons, which was similar to the exon numbers of *CmCRKs*. Furthermore, group CDPK IV clustered with CRK I rather than with the other three CDPK groups in Figs 3 and 4, indicating that they originated from a common ancestor [16,22]. Previous studies reported that intron loss happens more easily than intron gain during evolution [78]. The distance tree and gene structure (intron number and intron phase) suggest that the ancient ancestor of *CmCDPK* genes may have a similar intron number with *CmCRKs* in the early time, but experienced intron lost events during the process of evolution. Therefore, we propose that CDPK IV, which contains 12 exons, may be a more ancient lineage of the *CDPK* gene family.

### Function of *CDPK* and *CRK* genes in response to stimuli

An increasing number of studies have confirmed the crucial role of *CDPKs* and *CRKs* in plant stress response and in related signaling pathways [64]. Consequently, we investigated the expression of *CmCDPKs* and *CmCRKs* under biotic stress (powdery mildew), abiotic stress (salt and cold), and hormonal (ABA) treatment. In our study, the majority of *CmCDPKs* and *CmCRKs* were transcriptionally modified by salt, cold, ABA, and powdery mildew inoculation (Figs 6–8). Some genes showed similar expression patterns under a specific stress. As shown in S1 Fig, *CmCDPK5*, *CmCDPK6*, *CmCDPK8*, and *CmCDPK9* were strongly down-regulated due to *P*. *xanthii* inoculation, *CmCDPK9*, *CmCDPK12*, and *CmCDPK17* were down-regulated in response to salt stress, *CmCDPK2*, *CmCDPK4*, *CmCDPK10*, *CmCDPK13*, and *CmCDPK18* were up-regulated due to cold stress, and most *CmCDPKs* were down-regulated after ABA treatment. These observations indicate that some *CDPK* genes could cooperatively regulate a specific stimulation, consistent with the findings of many plants, such as *CDPK* gene in tomato [16], poplar [21], cucumber [23], canola [29] and grape [32].

Some *CmCDPKs* and *CmCRKs* were found to respond to various stresses with different expression patterns in the current study. For instance, *CmCDPK15* was up-regulated by cold stress, but down-regulated by salt stress (Fig 7). Transcriptional abundance of *CmCDPK9* was down-regulated by both salt and ABA treatments, while cold stress did not result in any changes (S1 Fig). *CmCRK2* was up-regulated under cold stress, but strongly down-regulated after powdery mildew inoculation (S1 Fig). Notably, *CmCDPK8* was down-regulated in all the responses to powdery mildew inoculation, salt, cold, and ABA treatment, indicating its multiple functions in plant response to diverse stimuli (S1 Fig). These results imply complex functionality of *CmCDPKs* and *CmCRKs* in multiple signaling pathways, which is consistent with previous studies [7].

There are twenty-two collinear blocks of CDPK and CRK genes between melon and Arabidopsis (Fig 2; S2 Table). Intriguingly, we found correlated functional connections in syntenic genes. For example, *AtCPK6* was found to significantly improve salt tolerance [79], and its collinear gene *CmCDPK2* was also up-regulated during salt stress. Both *CmCDPK8* and its collinear gene *AtCPK32* were involved in the response to ABA stress [80]. The synteny analysis of melon and Arabidopsis may provide insights into the prediction of gene function for CDPK and CRK genes.

It has been widely known that *CDPKs* play critical roles in different aspects of plant response to abiotic stresses. In cucumber, almost all *CsCDPKs* were up-regulated under cold stress, but only half of them were induced at the transcriptional level after exposure to high salinity [23]. However, the majority of *CmCDPKs* in melon were up-regulated under both cold and salt stresses (Fig 7). It is interesting to point out that all the identified *CmCDPK* genes (except for *CmCDPK5* and *CmCDPK14*) were strongly down-regulated at 12 hpt with ABA treatment, while most cucumber *CsCDPKs* still remain at high expression levels at 12 hpt with exposure to ABA stimuli [23].

In the present study, we performed an in-deep analysis of two pairs of segmental duplications, *CmCDPK7/ CmCDPK18* and *CmCDPK9/ CmCDPK18*. Although *CmCDPK7* and *CmCDPK18* had similar exon-intron structures in terms of exon number and intron phase (Fig 4), their expression profiles were different in response to stress. They were both up-regulated by salt stress but the extent was stronger in *CmCDPK7* than in *CmCDPK18*, which was similar for powdery mildew treatment (Figs 6 and 7). Based on the observations mentioned above, we suggest that *CmCDPK7* and *CmCDPK18* may have undergone subfunctionalization, which greatly adds gene specific functionality [81]. We found distinct expression profiles between *CmCDPK9* and *CmCDPK18*. For instance, *CmCDPK9* was down-regulated but *CmCDPK18* was up-regulated due to salt stress and powdery mildew inoculation (Figs 6 and 7). Furthermore, their exon numbers and intron phases were apparently different, and *CmCDPK9* belonged to *CDPK* II while *CmCDPK18* belonged to *CDPK* III in the phylogenetic tree (Figs 3 and 4). Above all, the two genes might undergo a divergent process of structure and function in their evolutionary history.

## Conclusions

We identified 18 *CDPK* and 7 *CRK* genes in the melon genome that were unevenly mapped among 11 chromosomes. Based on gene structure and phylogenetic tree analyses, *CmCDPKs* were divided into four groups with conserved domain. Synteny analysis identified two segmental duplication events of *CmCDPKs* and 22 syntenic blocks of *CDPK* and *CRK* genes among melon and Arabidopsis. Expression profile analysis of *CmCDPKs* and *CmCRKs* due to various stresses implied their crucial roles in response to multiple signaling pathways. This study provides important insights into the evolution and function of *CDPK* and *CRK* genes in melon, which will be vitally useful to melon breeding programs.

## Supporting information

S1 Data Sheet Full-length protein sequence of *CDPK* and *CRK* gene from melon, Arabidopsis, tomato and rice. (DOCX)

S1 Fig Heatmap showing the expression patterns of *CmCDPKs* and *CmCRKs* under biotic (powdery mildew) and abiotic (salinity and low temperature) stresses as well as ABA treatment. (TIF)

S1 Table The qRT-PCR primers of *CDPK* and *CRK* genes in melon. (XLS)

S2 Table The synteny regions between melon and Arabidopsis *CDPK* genes. (XLSX)
