# Supplementary material for: Genome-wide identification and expression analysis of calcium‑dependent protein kinase and its related kinase gene families in melon (Cucumis melo L.)
Source: PLoS One. 2017 Apr 24;12(4):e0176352. doi: 10.1371/journal.pone.0176352 (PMC5402965; doi:10.1371/journal.pone.0176352)
Supplement: S1 Data Sheet — (DOCX) [file pone.0176352.s001.docx]

**Sheet 1** Full-length protein sequence of *CDPK* and *CRK* gene from melon, *Arabidopsis*, tomato and rice

>CmCDPK1

MEKPIKASQSSSTKVASSVLPYQTPRLRDHYLLGKKLGQGQFGTTYLCTHRATGDLYACKSIPKRKLLCKEDYEDVWREIQIMHHLSEHPNVVQIKGTYEDSVFVHLVMELCAGGELFDRILLKGHYSEREAAKLIKTIVGVVETCHSLGVMHRDLKPENFLFDNPGEDAKLKATDFGLSVFYQPGNSFCDVVGSPYYVAPEVLRKHYGHEVDVWSAGVILYILLSGVPPFWAETDSGIFRQILYGKLDFNSEPWPNISDSAKDLITKMLTRDPKERISAHEVLCHPWIVDDTVAPDKPIDSAVLSRLKQFSAMNKLKKMALRVIAERLSEEEIGGLKELFKMIDTDNSGSITFEELKAGLKKVGSELMESDIKSLMDAADIDNSGTIDYGEFLAATLHLNKIEREDNLVAAFSYFDKDGSGYITIDELQQACKDFGLGDVHLDEIIKEIDQDNDGRIDYEEFAAMMRKGDGGVGRSRTMRSNLNFNLADAFGIKDKPLEN

>CmCDPK2

MGNTCRGSFKGNIFQGYSQPEDSSTPNSKRNTNTSADHSPSSINTNNLISQEFAKENKIKSKDNDCNPTFLSPTKKDYTMRKSAENQAYYVLGHKTANIRDLYTLGRKLGQGQFGTTYLCTEITTGIEYACKSISKRKLIAKEDVEDVRREIQIMHHLAGHKNIVTIKGAYEDSLYVHIVMELCSGGELFDRIIQRGHYSERKAAELTRIIVGVVETCHSLGVMHRDLKPENFLLVNKDDDFSLKAIDFGLSVFFKPGQIFTDVVGSPYYVAPEVLLKHYGPAADVWTAGVILYILLSGVPPFWAETQQGIFDAVLKGHIDFDSDPWPLISDSAKDLIRKMLCSRPSDRLTAHEVLCHPWICENGVAPDRALDPAVLSRLKQFSAMNKLKKMALRVIAESLSEEEIAGLREMFTAMDTDNSGAITFDELKAGLRRYGSTLKDIEIRDLMDAADIDNSGTIDYGEFIAATIHLNKLEREEHLVAAFRYFDKDGSGYITVDELQQACAEHNMTDVYLEDIIREVDQDNDGRIDYGEFVAMMQKGNAGIGRRTMRNSLNLSMRDGPGAL

>CmCDPK3

MGNCCATPATPSGQQHKGKNKKQNPFAADYVVSNGNGGGNWVLKDPTGRDISALYDLGIELGRGEFGVTYLCTDRNTGEKLACKSISKKKLRTAVDIDDVRREVEIMKNLPKHPNIVSLRDTYEDEHAVHIVMELCEGGELFDRIVARGHYTERAAAVVMRTIVEVVQMCHKHGVMHRDLKPENFLFGNKKETTPLKAIDFGLSVFFKPGERFNEIVGSPYYMALEVLKRNYGPKVDVWSAGVILYILLCGVPPFWAETEQGVAQAIIRSVIDFKRDPWPIVSDNAKDLVKKMLDPDPKRRLTAQEVLEHPWLQNAKKAPNVSLGETVKARLKQFSVMNKLKKRALRVIAEHLSVEEVAGIKEAFEMMDTGKRGKINLDELRVGLQKLGQQIPDPDLQILVEAADLDGDGTLNYSEFVAVSVHLKKMANDEHLHKAFSFFDKNQSGYIEIEELRNALNDDDETNGEDVINAIMHDVDTDKDGRISYEEFAAMMKAGTDWRKASRQYSRERFNSLSLKLMRDGSLHLTNEAR

>CmCDPK4

MGLCFTRTRDIPIDSFNNGSDTPNPQIKPQSQNPPPPSKPQQIPSYKSVPPSQIGPITGRPYINITTLYELHKELGRGQFGITYLCTEKSTGRKYACKTISRRKMVNPKDIEDVKREILILQHLTGQPNIVEFKGAYEDKRNLHLVMELCSGGELFDRIIKKKSYSEREAASICKQILNVVQACHFMGVMHRDLKPENFLMVSQDEDSPIKATDFGLSVFIEEGKVYRDMVGSAYYIAPEVLQRNYGKEIDMWSAGVILYILLCGEPPFWGKTEDDILKEVRKGILKMEDAPWPSISSSAKDLVSKMLTRNPKKRITAAEALEHPWLKIEGEASTKPIDSAVLIRMRQFRAMNKFKQLALKVMAENLSEEELKGLKQMFTNIDTDRSGTITFDELKTGLSRLGSRLSEHEIKQLMDAADVNRNGTIDYDEFITATMHRHRLDKEENMYKAFQFFDIDGSGLITRDELKQAMSQYGIGDEDTIDEIINDVDIDGDGKINYDEFVNMMTKGTVDAKMN

>CmCDPK5

MGNTCVGPNLGNNGFLKSVTAAVWPSRPPEERLPPPKDGADSKSKTNASSDSSKGADASKDSEPQKDVQTQSTPPETVKIGNNDQNKVVEREMSSRVIKDETRPPEGTKSKKGTHIKRLSSAGLQIDSVLGRKTDNIKDHYTLGRKLGQGQFGTTFLCVEKASGKEFACKSIAKRKLTTKEDVEDVRREIQIMHHLAGHPNVIQIVGAFEDAVAVHVVMELCAGGELFDRIIQRGHYTERKAAQLARIIVGVVEACHSLGVMHRDLKPENFLFINQQEESALKTIDFGLSMFFRPGETFTDVVGSPYYVAPEVLRKLYGPECDVWSAGVIIYILLSGVPPFWDETEQGIFEQVLKGDLDFISEPWPSISGEAKDLVRRMLVRDPKKRLTAHEVLCHPWVKADGVAPDKPLDSAVLSRLNQFSAMNKLKKMAIRVIAESLSEEEIAGLREMFKMIDTDNSGQITLEELKHGLERVGANLEDSEISGLMQAADVDNSGTIEYGEFVAAMLHLNKIQKEDHLFAAFSYFDKDGSGYITQDELQQACEKFGLSDIRLEDIMREVDQDNDGRIDYSEFVAMMQDTGYGQR

>CmCDPK6

MGNNCLRRNSKGGLFYSISHPIWWSRSEMDPHTKKGVSNKGIKNPNPNPNPNEEVQNTPPEPVMIVDERAKPLECEAKPVEVIKPKEEDIIIIKPLEPLVPMDDLNKPAEIPPRYVEERLPVELLIPKPADEPPKRIEERMPPIESALPKEDSSGGGNNDNVSEDLIKMKKPKKIVSAGLMVESVLQTKTGHLREFYSLGRKLGHGQFGTTFLCVEKSTGKEYACKSIAKRKLATMEDVEDVRREIQIMHHLIGTPSIVSIKGAYEDAVAVHVVMELCEGGELFDRIVKLGHYTERQAAELARTIIGVIEACHSLGVMHRDLKPENFLFVDSREDSPLKAIDFGLSIFFKPGDIFSDVVGSPYYVAPEVLCKLYGPESDVWSAGVILYILLSGVPPFWAETEQEIFDEVLHGDLDFTLDPWPSISDSAKDLVRKMLIRNPKERLTAHEVLCHPWLQVDGVAPDKPLDSAVLSRLKQFSAMNKLKKMALRVIAESLSEEEIAGLKEMFKMIDTDNSGQITFEELKDGLRRFGANLNETEIKDLMQAADFDNNGCIDYGEFIAATLHLNKAGREDHLFAAFQYFDKDGSGYITQDELQKACEEFGIENVHLEDMIREVDQDNDGRIDYNEFVTMMQKGNGELGKKGQQNTNFSIGFREALPVC

>CmCDPK7

MGNCCRSPAAVAREDVKSSFSGQDHSRRDSNAGKKVPVTVLNGVPKENIEEKYMVDRELGRGEFGVTYLCIDRQTRDLLACKSISKRKLRTAVDIEDVRREVAIMKHLPKNSSIVSLKEACEDENAVHLVMELCEGGELFDRIVARGHYTERAAAAVMRTIVEVVQLCHKHGVIHRDLKPENFLFANKKENSPLKAIDFGLSIFFKPGEKFSEIVGSPYYMAPEVLKRNYGPEIDIWSAGVILYILLCGVPPFWAETEQGVAQAILRGLIDFKRDPWPSISESAKSLVKQMLEPDPKLRLTAKQVLDHPWLQNIKKAPNVPLGDVVKSRLKQFSMMNRFKRKALRVIADFLSTEEVEDIKEMFKKIDTDNDGIVNIEDLKAGIHNFSSQLAEPEIQMLIEAVDTNGKGTLDYGEFVAVSLHLQRMANDEHLRKAFSYFDKDGNGFIEPDELRDALVEDGADDCTDVANDIFQEVDTNKDGLISYEEFVAMMKTGTDWRKASRHYSRGRFNSLSIKLMKDGSLNLGSE

>CmCDPK8

MGNCCATPSTPSAKPKGKNKKTNPFSAKDVEKNVNGHGNKLQVLSDPTGHNISAHYDLGRELGRGEFGVTYLCTDTNTGEKFACKSISKKKLRTSVDIEDVRREVQIMKHLPNHPNVVMLKDTYEDDQAVHIVMELCEGGELFDRIVARGHYTERAAAVVMRTIVEVVQACHKHGVMHRDLKPENFLFANKKESSPLKAIDFGLSITFKPGDVFNEIVGSPYYMAPEVLKRNYGPEVDVWSAGVILYILLCGIPPFWAETEQGVAQAIIRSVIDFRRDPWPRVSENAKDLVRKMLNPDPKRRLTAQEVLDHPWLQNAKKAPNVPLGETVKARLKQFSVMNKLKKRALRVIAEHLSVEEVAGIKEAFDMMDVEKRGKINLEELRSGLQKLGQQIPEADLQILIEAAGVQDDGALNYGEFVAVSIHLKRMANDEHLHKAFAFFDQNQSGFIEIEELRAVLLDDEETNSEDVVSAIMHDVDTDKDGRISYEEFAAMMKAGTDWRKASRQYSRERFNSLSLKLFREGSLKLTS

>CmCDPK9

MGNCCSRENPDEAPATSVKEESNNADNSKDDGGNTNHGNSTNNPSSKTPPSASPAASTKPSKPSQIGTVLGRPMEDVRSTYSIGKELGRGQFGVTHLCTHKASGEQLACKTIAKRKLVNKEDIEDVRREVQIMHHLTGQPNIVELKGAYEDKHSVHLVMELCAGGELFDRIIAKGHYTERAAASLLRTIVQIVHTCHSMGVIHRDLKPENFLLLSKDEDSPLKATDFGLSVFYKQGEVFKDIVGSAYYIAPEVLKRRYGPEVDIWSVGVMLYILLCGVPPFWAESEHGIFNAILRGHIDFTSDPWPSISPAAKDLVRKMLNSDPKQRLSAFQVLNHPWIKEDGEAPDTPLDNAVLNRLKQFRAMNKFKKVALRVIAGCLSEEEIMGLKQMFKSMDTDNSGTITLEELKQGLAKQGTKLSEYEVKQLMEAADADGNGTIDYDEFITATMHLNRMDREEHLYTAFQYFDKDNSGYITTEELEQALREYGMHDGRDIKEILSEVDADNDGRINYDEFVAMMRKGNPEANPKKRRDVFV

>CmCDPK10

MGICTSKGKYSRQYDRYEQQPKAPAQHAHRSPEYHQPAAAAKSSGPPKSPKHTPFRSDTILGKPLEDVKLHYTIGKELGRGQFGVTYLCTENSSGKQYACKSILKRKLVTRNDKEDIRREIHIMQHLSGQPNIVEFKGAYEDKQSVHLIMELCAGGELFDRIIAKGHYSEKAAASICRSIVNVVHICHFMGVMHRDLKPENFLLASKEEDAMLKATDFGLSVFIEEGKVYRDIVGSAYYVAPEVLRRKYGKEIDIWSAGVMLYILLSGVPPFWAETEKGIFDSILQGHIDFESEPWPQISHSAKDLVKKMLTQDPANRITSAQVLEHPWMREGGDASDKPIDSAVFTRLKQFRAMNKLKKLALKVIAENLSEEEIQGLKAMFTNMDTDKSGTITYAELKSGLARLGSTLSEAEVKQLMEAADVDGNGTIDYIEFITATMHRYKLEKEEHLYKAFQHFDKDNSGYITRDELKAAMKDYGMGDEETIREIISEVDADNDGRINYDEFCSMMRSGTQQAKLF

>CmCDPK11

MGNCSGLPTGQTSTAYPLGHSDADYRPPSNGVVKVLPPDSDPSLPPPLPKEISTSSAPPAVGRVLGRPFSDVRSFYSFRGELGRGQFGVTYLVTHKETKQDFACKSIATRKLIDQDDVEDVRREVQIMHHLTGHPHIVELKEVYEDRHYVNLIMELCAGGELFDRIIAKGHYSECTAASLCRQIVTVVHNCHSMGVMHRDLKPENFLFLSADENSPLKATDFGLSVFFKPGDVFKDLVGSAYYVAPEVLRRRYGPEADVWSAGVILYILLSGVPPFWAENDQGIFDAVLRGHIDFSSDPWPSISSGAKDLIRKLLHSDPKERLSAIEALNHPWMKEDGEASDKPLDIAVLTRMKQFRAMNKLKKVALKVIAENLSEEEIVGLKEMFKSMDTDNSGTITFEELKAGLPKLGTKLSESEVRQLMEAADVDGNGSIDYIEFITATMHMNRVEREDHLFRAFEYFDKDKSGYITMEELETALKKYNMGDEKTIKEIIAEVDTDNVQFLLSQ

>CmCDPK12

MGSCVSVQPRPGSFSKRPKDINPLPDHVLIESVRKSSVRHAASILSHDSTGDNIFEKYRFGKELGRGEFGITHQCFDIETGDTFACKTISKSKLRSEINVEDVRREVAIMRSLPKHPNIVTFKEAFEDNEAVYFVMELCEGGELFDRIVSKGHYTERAAANVTKTIVEICKVCHENGVIHRDLKPENFLFADESENSQLKAIDFGLSIFFEPDQRFGEIVGSPYYMAPEVLRRSYGPEIDVWSAGVILYILLCGVPPFWAESEEGIAHAIVRGNIDFERDPWPKVSKEAKELVVGMLDPNPYNRMTVEEVLAHPWIQNKNQARNVSLGENVGIRIKQFTLMNKFKKKVLRVVADHLSDEQMEGIRRMFHMMDTDQNGDLTFEELKNGLHMIGHSLPDPDVRMLMDAADLDGNGTLSCEEFATMSIHLRKMSTDELLTQAFNFFDKNQNGYIEYDELREALMDDNEKVIQDIISDVDSDKDGRISYEEFKAMLTTGMDWKMSSRQYSRAMLHALSLKLFKDKSVAVQN

>CmCDPK13

MGCCSSTQKPPSSGINGFDAARGVATRGPYQPYQPQSTVPIPHKVSAPQTQIPSNRNQPQPPPTAEHPPSASAFSKPAVSTPRTAQNYPETILGRPYDDIKKYYTLGKELGRGQFGITYLCTENSTGQTYACKSILKRKLISKNDKDDMKREILILQHLSGQPNIVEFKGAYEDRFSVHLVMELCAGGELFDRIIAKGQYSEKAAAEICRAIVNVVQICHFMGVMHRDLKPENFLLASKKEDAMLKATDFGLSVFIEEGKVYRDIVGSAYYVAPEVLRRNYGKEIDVWSAGVILYILLSGVPPFWAENEKGIFDAILQGDIDFASAPWPTISESAKDLVRKMLMQDPKKRITPAQVLDHPWIREGGEASDKPIDNAVLSRMKQFRAMNKLKKLALKVIAENLSEEEIKGLKAMFANIDTDNSGTITYEELKTGLARLGSRLSEAEVKQLMEAADVDGNGSIDYIEFISATMHRHRLERDEHLYKAFQFFDKDSSGYITKDELETAMKDYGMGDEASIREIISEVDTDNDGRINYQEFCAMMRSGTTQPGKLF

>CmCDPK14

MGVCFSASKVSGSNSNTNNTAVNHHRRGSTANPQPSTTDTSQNQKRPNDNPKRNSQQLKIKEKNVSRRQSGVIPCGKRTDFGYHKNFDERYTIGKLLGHGQFGYTYVAVDKANGDRVAVKKIEKNKMILPIAVEDVKREVKILQELTGHENVVQFHNAFEDDSYVYIVMELCEGGELLDRILSKKDSRYTEKDAAVVVRQMLKVAAECHLHGLVHRDMKPENFLFKSAKEDSHLKATDFGLSDFIKPGKKFHDIVGSAYYVAPEVLKRKSGPESDVWSIGVITYILLCGRRPFWDKTEDGIFKEVLRNKPDFRRKPWPSISPSAKDFIKKLLVKDPRARLTAAQALSHRWVREGGDASEIPIDISVLSNMRQFVKYSRLKQFALRALASTIGEEELADLRDQFDAIDVDKNGSISLEEMRQALAKDLPWKLKESRVLEILQAIDVNTDGLVDFTEFVAATLHVHQLEEHDSVKWQQRSQAAFEKFDIDKDGFITPEELRMHTGLKGSIDPLLEEADIDKDGKISLSEFRRLLRTASISSRPHI

>CmCDPK15

MGNCCVAPPRNPEDQNKGKRKKKPNPFSIDYGVNHFAGGNGGSHKLTVLTNPTGCEIGLQYELGRELGRGEFGITHLCTDKVTGEKFACKSISKKKLRTAVDIEDVRREVQIMRHLPKHQNIVSLKDTFEDDNAVHLVMELCEGGELFDRIVARGHYTERAAAVVTKTIVEVVQMCHKHGVMHRDLKPENFLFGNKKENAPLKAIDFGLSVFFKPGERFNEIVGSPYYMAPEVLKRNYGPEVDVWSAGVILYILLCGVPPFWAETEQGVAQAIIRSVIDFKRDPWPKVSDNAKDLVRKMLDPDPKRRLTAQGVLDHPWLQNVKKAPNVSLGETVRARLKQFSVMNKLKKRALRVIAEHLSVEEVAGIKEGFEKMDTGNKGKINIDELRVGLHKLGHQIEDADLQILMEAGDVDNDGYLDCREFVAISVHLRRIGDDEEHLRKAFDFFDQNLSGYIEIEELRSTLADEIDENSEEVINAIINDVDTDKDGRISYDEFATMMKAGTDWRKASRQYSRERFNSLSLNLMRDGSLQLKQ

>CmCDPK16

MGNTCVGPSISKNGFFQSVSAAMWRSRSPENSVSHHTNGESVHEVAASEPESPLPVQNQPPEKVTMPESQVKPEPPSEPKVRPNPVMKRVGSAGLRGGSVLQTKTGNFKEYYSLGKKLGQGQFGTTYMCVEKATGKEYACKSIAKRKLVTEDDVEDVRREIQIMHHLAGHPNVISIKGAYEDAVAVQVVMELCAGGELFDRIIQRGHYTERKAAELTRTIVGVVEACHALGVMHRDLKPENFLFVGKEEESLLKTIDFGLSVFFKPGEKFNDVVGSPYYVAPEVLRKRYSHEADVWSAGVIIYILLSGVPPFWAESEQGIFEEVLHGDLDFSSDPWPSISDSAKDLVRRMLVRDPKRRLTAYEVLCHPWVQVDGVAPDKPLDSAVLSRLKQFSAMNKLKKMAIKVIAESLSEEEIAGLKEMFKMIDTDNSGQITFEELKAGLKKFGANLKESEIYDLMQAADIDNNGTIDYGEFVAATLHLNKIEKEDHLLAAFSYFDKDGSGFITHDELQQACKEFGIEDLQLEEMMHEVDQNNDGTIDYNEFVAMMQKGNVTNAGKKGLQSTFSIGFREALKL

>CmCDPK17

MSKSSSAAPPPSAKPSWVLPYRTQPLTDFYTLGKKLGQGQFGTTFLCTDKQTGFNYACKTIPKRKLLCKEDYEDVWREIQIMHHLSEHPNIVRIKGTYEDPVSVHLVMELCEGGELFDRIVQKGQYSEREAAKLIGVIVSVLESCHSLGVMHRDLKPENFLFQSVDEDAALKATDFGLSVFYKPGETFSDVVGSPYYVAPDVLRKHYGPESDVWSAGVILYILLSGVPPFWAETEIGIFRQILQGRLDFESEPWPGISASAKDLIRKMLDRNPKRRLTAHEVLCHPWIVDDKVAPDKPLDSAVLSRLKQFSAMNKLKKMALRVIAERLSEEEIGGLKELFKMIDTDNSGTITFDELKEGLKRVGSELMESEIKDLMDAADIDNSGTIDYGEFLAATIHLNKLEREENLLSAFSYFDKDGSGFITIDELQLACKEFGLSELHLDDMISEIDEDNDGRIDYGEFAAMMRKGNGGVGRRTMRGPMNLGEALGLSASANNQSIDNPT

>CmCDPK18

MGNCNACVRPETTEDEKSVNRKKNEKERKSNPYTRETATRSPAPLRVLKDVMPLSHRTRISDKYILGLELGRGEFGITYLCTDRETKQALACKSISKRKLRTAVDIEDVRREVAIMSNLPEHPNIVKLKATYEDNENVHLVMELCEGGELFDRIVARGHYTERAAANVARTIAEVVRMCHANGVMHRDLKPENFLFANKKEHSPLKAIDFGLSVFFKPGEKFSEIVGSPYYMAPEVLKRNYGPEVDIWSAGVILYILLCGVPPFWAETEQGVALAILRGVLDFKREPWPQISESAKSLVRQMLEPEPKKRLTAQQVLDHPWLQNAKKAPNVPLGDIVRMRLKQFSVMNRFKKKALRVIAEHLSVEEVEVIRDMFSLMDTDNDGKVSFEELKAGLKKVGSQLAEPEMKMLMEVADVDGNGVLDYGEFVAVTIHLQRMENDEHFRRAFMFFDKNESGFIELDELRVALSDESGETDSDVLNEIMREVDTDKDGQISYDEFVAMMKTGTDWRKASRQYSRERFKSLSLNLMKDGSLQLHDGLTGQAVVV

>CmCRK1

MGICTSKPPPKPNPYAPRDPDGRIDPSHTPKSAPTPHRKDDLVAGKQSPFFPFFSPSPSPYFSKKKSQNSPLPGGGPESATSTPGRTPGRFFRRSFAPPSPAKHIRAVLARRLGKKAGSTAAIPEEGDEESGIELDKRFGFSKELTSRLEVGEEVGRGHFGYTCSAKFKKGEHKGQQVAVKVIPKAKMTTAIAIEDVRREVKILRALTGRTNLVQFYDAFEDHDNVYIVMELCEGGELLDRILSRGGKYSEEDAKAVMVQILTVVAFCHLQGVVHRDLKPENFLYTSKDENAQLKAIDFGLSDFVKPDERLNDIVGSAYYVAPEVLHRSYGTEADVWSIGVIAYILLCGSRPFWARTESGIFRAVLKADLSFDEGPWPSLSFEAKDFVKRLLNKDPRKRLTAAQALSHPWIRNHKGAKVPIDILIFKVMRIYMRSSSLRKAALRAVSKTLTVDELSYLKEQFELLEPNKNGFITLETIKMGLAKHATDAMNESRTLDFLANLNTLQYRGMDFDEFCAAALSIHQLEALDRWEQHARCAYEIFEKNGNRAIVIEELASELGLGPAIPLHVVLQDWIRHTDGKLSFLGFVKLLHGVSSRSLAKVS

>CmCRK2

MGLCHGKPIEQNPKPNSENPNSVIQSETPKTPTSFPFYSPSPLPNLFKNSPANSSLTSTPLRLFKRPFPPPSPAKHIRALLARRHGSVKPNEASIPEGSECDVALDKNFGYSKHFAAHYDLGEEVGRGHFGYTCSARAKKGSFKGQQVAVKIIPKSKMTTAIAIEDVRREVKILRALTGHKNLVQFYDSYEDEENVYVVMELCEGGELLDRILSRGGKYSEEDAKVIMVQILSVVAYCHLQGVVHRDLKPENFLFTSKDETSTLKAIDFGLSDYVKPDERLNDIVGSAYYVAPEVLHRSYGTEADMWSIGVIAYILLCGSRPFWARTESGIFRAVLKADPNFEEAPWPSLSIDAIDFVKRLLNKDYRKRLTAAQALCHPWLADHQDIKIPLDTITFKLVRSYICSSSLRKSALGALAKTLSAVQLGYLQKQFTLLGPNKNGLISMQNFKTALIKNSTDAIKDSRVLDYANVVSSIQYRKLDFEEFCAAAISIYQLEGMENWEQHARHAYDLFDKDGNRPIMIEELASELGLSPSVPVHVVLQDWIRHSDGKLSFLGFVRLLHGVSSRAFQKA

>CmCRK3

MGLCVSKPSSTTVVAVPAIQDALPPLETSLPSVDTGNRSNETREGEKIPFDLEVAKKSPYFPPFTPSPVHYLFSKKSPLRSPPNVSSNSTPKRFFKKPFPPPSPAKHIMAVLARRHGSVKPNEASIPEGNVIEEPGLDKSFGFSKHFRNKYELGEEVGKGHFGHTCRATVKKGELKGKQVAVKVIPKAKMTTAIAIEDVRREVKILKALSGHKNLVHFYDACEDHDNVYIVMELCEGGELLDRILSRGGKYTEDDAKSVLIQILNVAAFCHLQGVVHRDLKPENFLYTSKDESSQLKAIDFGLSDFVKPDERLNDIVGSAYYVAPEVLRRSYGTEADVWSIGVIAYILLCGSRPFWARTESGIFKAVLKADPNFDDPPWPSLSSESRDLVKGLLNKDPEKRLTAAQALSHPWFKDSKEVKIPLDVHLLKLMRVYMCSSSLRKAALKALSKTLSIDEQGYFKIQFALLEPNKNGTISLENIKETLMKNGTEAMKESRTIDFLASLNALQFRRMDFEEFCAATLSVRQLEDLGHWEQLARSAYEAFEKDGNRAIMIEELASELGLSPSAPVHSVLQDWIRHTDGKLSFLGFVKLLHGPSSRAGRRQ

>CmCRK4

MGICQAKVIANPKAKCEEDNTDSQTRNPTPNLPFFTPSPLSSVFKNSPSVGTKRRSRSRSRPFPPPSPAKHIKELLARRHGSGKPNQVAIPENEEGEGEVVEVNREFGYSKQFVGNYEIGEEVGRGHFGFTCKAKAKKGSLKGRNVAVKIIAKAKMTTTIAVEDVKREVKILQALNGHKNLVQFHGAFEDDINVYIIMELCEGGELLDRMLLRGGMYSEEDAKTVLVQVLSAIAFCHLQGIVHRDLKPENFLFTTKEEDSTLKVIDFGLSDYVNPDEKLNDIVGSAYYVAPEVLQRSYGTEADMWSIGVIAYILLCGSRPFWDRTESGIFRTVLREDPSFDDEPWPSLSPDAKDFVQRLLHKDQHKRLTAAQALCHPWLTNHPNVKIPLDMIVYKLVRAYIYSSSLRKLALNALAKTLSFVQLAYLREQFDLLGPNKNGFISLQNFKMALMKASTDAMNDSGVIEYVDMVSSSQYRRLDFEEFCAAAISVHQLKGMDSWEQHARYAYDLFEEYGNRTIMIEELASELGLAPSVPLQVVLQEWIRPSDGKLSFMGFIKLLNGVPPSTNKKT

>CmCRK5

MGHYCSKGVSDVSNHPSNSGIPQHTTPSPFSSPLPPGIPPSPATTPGRKFRWPLPPPSPAKPIMAILRRKSRKPSIPEDGKSGGEELGEVQLDKSFGYSNNFAAKFELGMEVGRGHFGHTFWAKGKKGDLKGIPVAVKIISKSKNWLWQMTSAVGIEDVRREVKILKALSGHDNLVHFHDAFEDANNIYMVMELCEGGELLERIASRGGKYPEQESKAIIVQILSVVAFCHLQGVVHRDLKPENFLFLKKNENTGLKVIDFGLSDFVKPDERLNDVVGSAYYVAPEVLYRSYSFEADIWSIGVVAYILLCGGRPFWARTESGIFRSVLRADPNFDDSPWPNISAEAKDFVKRLLNKDHRKRMTAAQALTHQWLRDENIAVPLDNFIYKSVKAYIRATPFKRAALKALAKALTEDELFYLRTQFKLLEPQNRFVTLDNFKAALARNATDAMRESHVADILKMMEPLARERMDFEEFCAAAISVHQLEAVAGWESIATRAFEYFEQEGNRVISVHELVQEMNLGPAAYSFLQNWTRSSDGKLSFFGFTRFLHGVTVRNSNTRH

>CmCRK6

MGLCNSKPSPNSDLFAEKSGSRTPNEGSNSTQPNSVTAMGGASPLPPSSENGRDGRKTGNRRSESHEMDNVKKSPFFPFYSPSPAHYLFSKKSPARSPANASANSTPKRFFRKPFPPPSPAKHIRAVLARRHGSVKPNEVSIPEGSEAEGVTGLDKNFGFSKHLGSKYELGEEVGRGHFGYTCAAKFKKGELKGQQVAVKIIPKSKMTTAIAIEDVRREVKILKSLSGHKNLVNFYDAYEDHDNVYIVMELCEGGELLDRILARGGKYTEEDARAVMTQILYVVAFCHLQGVVHRDLKPENFLFTSKDENSPLKAIDFGLSDFAKPDERLNDIVGSAYYVAPEVLHRSYSTEADVWSIGVIAYILLCGSRPFWARTESGIFKAVLKADPIFDEPPWPSLSSEAKDFVKRLLVKDPRKRMSAAQALSHPWIKNSMDVKAPLDILIFKLMKIYMRSSYLRKAALRAVSRTLTIDELFYLKMQFSLLEPSKNGTINIENIKEALMKNITNGMKESRIPDLLTSLSALQYRRMDFEEFCAAAVSIHQLEALDRWEQHARYAYDLFEKDGNRPIVIEELASELGLSPSVPVHAVLHDWIRHTDGKLSFLGFVKLLHGPSSRTLAKPS

>CmCRK7

MGQCYGKTIPTTDNGVTPTTITTTVSPPSTTGYGADPSHFPSGSGAGNGVVNVKTTTATTPARTSYPSPWPSPYPHGVGASPLPVGVSPSPARASTPGRFFKRRFAPPSPAKHIKASLAKRFGYTKPKEGPIPEERGAEPEQLLDKSFGYGKNFGAKYELGKEVGRGHFGHTCSAKGKKGELKDQPVAVKIISKAKMTTAISIEDVRREVKILKSLSGHKHLVKFHDACEDANNVYIVMELCEGGELLDRILSRGGRYTEEDAKNIVVQILSVVAFCHLQGVVHRDLKPENFLFTSRSEDADMKLIDFGLSDFVRPDERLNDIVGSAYYVAPEVLHRSYTLEADIWSIGVITYILLCGSRPFWARTESGIFRAVLRADPNFDDLPWPSVSPEAKDFVKRLLNKDYRKRMTAVQALTHPWLQDDSRRIPLDILIYKLVKSYLQATPFKRAALKALSKALSETELFYLRAQFALLEPNHDGRVGLDNFKMALMRNATDAMRESRVHEIVNSLEPLAYRRMDFEEFCAASISTHQLEALDRWEQIACVAFEHFEGEGNRVISVEELARELNLGSSAYSILKEWIRGDGKLSFLGYTKFLHGVTLRSSNTRHH

>AtCPK1

MGNTCVGPSRNGFLQSVSAAMWRPRDGDDSASMSNGDIASEAVSGELRSRLSDEVQNKPPEQVTMPKPGTDVETKDREIRTESKPETLEEISLESKPETKQETKSETKPESKPDPPAKPKKPKHMKRVSSAGLRTESVLQRKTENFKEFYSLGRKLGQGQFGTTFLCVEKTTGKEFACKSIAKRKLLTDEDVEDVRREIQIMHHLAGHPNVISIKGAYEDVVAVHLVMECCAGGELFDRIIQRGHYTERKAAELTRTIVGVVEACHSLGVMHRDLKPENFLFVSKHEDSLLKTIDFGLSMFFKPDDVFTDVVGSPYYVAPEVLRKRYGPEADVWSAGVIVYILLSGVPPFWAETEQGIFEQVLHGDLDFSSDPWPSISESAKDLVRKMLVRDPKKRLTAHQVLCHPWVQVDGVAPDKPLDSAVLSRMKQFSAMNKFKKMALRVIAESLSEEEIAGLKEMFNMIDADKSGQITFEELKAGLKRVGANLKESEILDLMQAADVDNSGTIDYKEFIAATLHLNKIEREDHLFAAFTYFDKDGSGYITPDELQQACEEFGVEDVRIEELMRDVDQDNDGRIDYNEFVAMMQKGSITGGPVKMGLEKSFSIALKL

>AtCPK2

MGNACVGPNISGNGFLQTVTAAMWRPRIGAEQASSSSHGNGQVSKEAASEPATDQVQNKPPEPITMPSSKTNPETKLKPDLEIQPEEKKEKVLAEETKQKVVPEESKQEVPPEESKREVVVQPESAKPETKSESKPETTKPETTSETKPETKAEPQKPKHMRRVSSAGLRTESVLQRKTENFKEFYSLGRKLGQGQFGTTFLCLEKGTGNEYACKSISKRKLLTDEDVEDVRREIQIMHHLAGHPNVISIKGAYEDVVAVHLVMELCSGGELFDRIIQRGHYTERKAAELARTIVGVLEACHSLGVMHRDLKPENFLFVSREEDSLLKTIDFGLSMFFKPDEVFTDVVGSPYYVAPEVLRKRYGPESDVWSAGVIVYILLSGVPPFWAETEQGIFEQVLHGDLDFSSDPWPSISESAKDLVRKMLVRDPKRRLTAHQVLCHPWVQIDGVAPDKPLDSAVLSRMKQFSAMNKFKKMALRVIAESLSEEEIAGLKQMFKMIDADNSGQITFEELKAGLKRVGANLKESEILDLMQAADVDNSGTIDYKEFIAATLHLNKIEREDHLFAAFSYFDKDESGFITPDELQQACEEFGVEDARIEEMMRDVDQDKDGRIDYNEFVAMMQKGSIMGGPVKMGLENSISISLKH

>AtCPK3

MGHRHSKSKSSDPPPSSSSSSSGNVVHHVKPAGERRGSSGSGTVGSSGSGTGGSRSTTSTQQNGRILGRPMEEVRRTYEFGRELGRGQFGVTYLVTHKETKQQVACKSIPTRRLVHKDDIEDVRREVQIMHHLSGHRNIVDLKGAYEDRHSVNLIMELCEGGELFDRIISKGLYSERAAADLCRQMVMVVHSCHSMGVMHRDLKPENFLFLSKDENSPLKATDFGLSVFFKPGDKFKDLVGSAYYVAPEVLKRNYGPEADIWSAGVILYILLSGVPPFWGENETGIFDAILQGQLDFSADPWPALSDGAKDLVRKMLKYDPKDRLTAAEVLNHPWIREDGEASDKPLDNAVLSRMKQFRAMNKLKKMALKVIAENLSEEEIIGLKEMFKSLDTDNNGIVTLEELRTGLPKLGSKISEAEIRQLMEAADMDGDGSIDYLEFISATMHMNRIEREDHLYTAFQFFDNDNSGYITMEELELAMKKYNMGDDKSIKEIIAEVDTDRDGKINYEEFVAMMKKGNPELVPNRRRM

>AtCPK4

MEKPNPRRPSNSVLPYETPRLRDHYLLGKKLGQGQFGTTYLCTEKSSSANYACKSIPKRKLVCREDYEDVWREIQIMHHLSEHPNVVRIKGTYEDSVFVHIVMEVCEGGELFDRIVSKGCFSEREAAKLIKTILGVVEACHSLGVMHRDLKPENFLFDSPSDDAKLKATDFGLSVFYKPGQYLYDVVGSPYYVAPEVLKKCYGPEIDVWSAGVILYILLSGVPPFWAETESGIFRQILQGKIDFKSDPWPTISEGAKDLIYKMLDRSPKKRISAHEALCHPWIVDEHAAPDKPLDPAVLSRLKQFSQMNKIKKMALRVIAERLSEEEIGGLKELFKMIDTDNSGTITFEELKAGLKRVGSELMESEIKSLMDAADIDNSGTIDYGEFLAATLHINKMEREENLVVAFSYFDKDGSGYITIDELQQACTEFGLCDTPLDDMIKEIDLDNDGKIDFSEFTAMMKKGDGVGRSRTMRNNLNFNIAEAFGVEDTSSTAKSDDSPK

>AtCPK5

MGNSCRGSFKDKLDEGDNNKPEDYSKTSTTNLSSNSDHSPNAADIIAQEFSKDNNSNNNSKDPALVIPLREPIMRRNPDNQAYYVLGHKTPNIRDIYTLSRKLGQGQFGTTYLCTEIASGVDYACKSISKRKLISKEDVEDVRREIQIMHHLAGHGSIVTIKGAYEDSLYVHIVMELCAGGELFDRIIQRGHYSERKAAELTKIIVGVVEACHSLGVMHRDLKPENFLLVNKDDDFSLKAIDFGLSVFFKPGQIFTDVVGSPYYVAPEVLLKRYGPEADVWTAGVILYILLSGVPPFWAETQQGIFDAVLKGYIDFESDPWPVISDSAKDLIRRMLSSKPAERLTAHEVLRHPWICENGVAPDRALDPAVLSRLKQFSAMNKLKKMALKVIAESLSEEEIAGLREMFQAMDTDNSGAITFDELKAGLRKYGSTLKDTEIHDLMDAADVDNSGTIDYSEFIAATIHLNKLEREEHLVAAFQYFDKDGSGFITIDELQQACVEHGMADVFLEDIIKEVDQNNDGKIDYGEFVEMMQKGNAGVGRRTMRNSLNISMRDA

>AtCPK6

MGNSCRGSFKDKIYEGNHSRPEENSKSTTTTVSSVHSPTTDQDFSKQNTNPALVIPVKEPIMRRNVDNQSYYVLGHKTPNIRDLYTLSRKLGQGQFGTTYLCTDIATGVDYACKSISKRKLISKEDVEDVRREIQIMHHLAGHKNIVTIKGAYEDPLYVHIVMELCAGGELFDRIIHRGHYSERKAAELTKIIVGVVEACHSLGVMHRDLKPENFLLVNKDDDFSLKAIDFGLSVFFKPGQIFKDVVGSPYYVAPEVLLKHYGPEADVWTAGVILYILLSGVPPFWAETQQGIFDAVLKGYIDFDTDPWPVISDSAKDLIRKMLCSSPSERLTAHEVLRHPWICENGVAPDRALDPAVLSRLKQFSAMNKLKKMALKVIAESLSEEEIAGLRAMFEAMDTDNSGAITFDELKAGLRRYGSTLKDTEIRDLMEAADVDNSGTIDYSEFIAATIHLNKLEREEHLVSAFQYFDKDGSGYITIDELQQSCIEHGMTDVFLEDIIKEVDQDNDGRIDYEEFVAMMQKGNAGVGRRTMKNSLNISMRDV

>AtCPK7

MGNCCGNPSSATNQSKQGKPKNKNNPFYSNEYATTDRSGAGFKLSVLKDPTGHDISLQYDLGREVGRGEFGITYLCTDKETGEKYACKSISKKKLRTAVDIEDVRREVEIMKHMPKHPNVVSLKDSFEDDDAVHIVMELCEGGELFDRIVARGHYTERAAAAVMKTIVEVVQICHKQGVMHRDLKPENFLFANKKETSALKAIDFGLSVFFKPGEQFNEIVGSPYYMAPEVLRRNYGPEIDVWSAGVILYILLCGVPPFWAETEQGVAQAIIRSVIDFKRDPWPRVSDSAKDLVRKMLEPDPKKRLTAAQVLEHTWILNAKKAPNVSLGETVKARLKQFSVMNKLKKRALRVIAEHLSVEEAAGIKEAFEMMDVNKRGKINLEELKYGLQKAGQQIADTDLQILMEATDVDGDGTLNYSEFVAVSVHLKKMANDEHLHKAFNFFDQNQSGYIEIDELREALNDELDNTSSEEVIAAIMQDVDTDKDGRISYEEFVAMMKAGTDWRKASRQYSRERFNSLSLKLMRDGSLQLEGET

>AtCPK8

MGNCCASPGSETGSKKGKPKIKSNPFYSEAYTTNGSGTGFKLSVLKDPTGHDISLMYDLGREVGRGEFGITYLCTDIKTGEKYACKSISKKKLRTAVDIEDVRREVEIMKHMPRHPNIVSLKDAFEDDDAVHIVMELCEGGELFDRIVARGHYTERAAAAVMKTILEVVQICHKHGVMHRDLKPENFLFANKKETSALKAIDFGLSVFFKPGEGFNEIVGSPYYMAPEVLRRNYGPEVDIWSAGVILYILLCGVPPFWAETEQGVAQAIIRSVIDFKRDPWPRVSETAKDLVRKMLEPDPKKRLSAAQVLEHSWIQNAKKAPNVSLGETVKARLKQFSVMNKLKKRALRVIAEHLSVEEVAGIKEAFEMMDSKKTGKINLEELKFGLHKLGQQQIPDTDLQILMEAADVDGDGTLNYGEFVAVSVHLKKMANDEHLHKAFSFFDQNQSDYIEIEELREALNDEVDTNSEEVVAAIMQDVDTDKDGRISYEEFAAMMKAGTDWRKASRQYSRERFNSLSLKLMREGSLQLEGEN

>AtCPK9

MGNCFAKNHGLMKPQQNGNTTRSVEVGVTNQDPPSYTPQARTTQQPEKPGSVNSQPPPWRAAAAAPGLSPKTTTKSNSILENAFEDVKLFYTLGKELGRGQFGVTYLCTENSTGKKYACKSISKKKLVTKADKDDMRREIQIMQHLSGQPNIVEFKGAYEDEKAVNLVMELCAGGELFDRIIAKGHYTERAAASVCRQIVNVVKICHFMGVLHRDLKPENFLLSSKDEKALIKATDFGLSVFIEEGKVYRDIVGSAYYVAPEVLRRRYGKEVDIWSAGIILYILLSGVPPFWAETEKGIFDAILEGHIDFESQPWPSISSSAKDLVRRMLTADPKRRISAADVLQHPWLREGGEASDKPIDSAVLSRMKQFRAMNKLKKLALKVIAENIDTEEIQGLKAMFANIDTDNSGTITYEELKEGLAKLGSKLTEAEVKQLMDAADVDGNGSIDYIEFITATMHRHRLESNENLYKAFQHFDKDSSGYITIDELESALKEYGMGDDATIKEVLSDVDSDNDGRINYEEFCAMMRSGNPQQQQPRLF

>AtCPK10

MGNCNACVRPDSKESKPSSKPKKPNRDRKLNPFAGDFTRSPAPIRVLKDVIPMSNQTQISDKYILGRELGRGEFGITYLCTDRETHEALACKSISKRKLRTAVDIEDVRREVAIMSTLPEHPNVVKLKASYEDNENVHLVMELCEGGELFDRIVARGHYTERAAAAVARTIAEVVMMCHSNGVMHRDLKPENFLFANKKENSPLKAIDFGLSVFFKPGDKFTEIVGSPYYMAPEVLKRDYGPGVDVWSAGVIIYILLCGVPPFWAETEQGVALAILRGVLDFKRDPWPQISESAKSLVKQMLDPDPTKRLTAQQVLAHPWIQNAKKAPNVPLGDIVRSRLKQFSMMNRFKKKVLRVIAEHLSIQEVEVIKNMFSLMDDDKDGKITYPELKAGLQKVGSQLGEPEIKMLMEVADVDGNGFLDYGEFVAVIIHLQKIENDELFKLAFMFFDKDGSTYIELDELREALADELGEPDASVLSDIMREVDTDKDGRINYDEFVTMMKAGTDWRKASRQYSRERFKSLSINLMKDGSLHLHDALTGQTVPV

>AtCPK11

METKPNPRRPSNTVLPYQTPRLRDHYLLGKKLGQGQFGTTYLCTEKSTSANYACKSIPKRKLVCREDYEDVWREIQIMHHLSEHPNVVRIKGTYEDSVFVHIVMEVCEGGELFDRIVSKGHFSEREAVKLIKTILGVVEACHSLGVMHRDLKPENFLFDSPKDDAKLKATDFGLSVFYKPGQYLYDVVGSPYYVAPEVLKKCYGPEIDVWSAGVILYILLSGVPPFWAETESGIFRQILQGKLDFKSDPWPTISEAAKDLIYKMLERSPKKRISAHEALCHPWIVDEQAAPDKPLDPAVLSRLKQFSQMNKIKKMALRVIAERLSEEEIGGLKELFKMIDTDNSGTITFEELKAGLKRVGSELMESEIKSLMDAADIDNSGTIDYGEFLAATLHMNKMEREENLVAAFSYFDKDGSGYITIDELQSACTEFGLCDTPLDDMIKEIDLDNDGKIDFSEFTAMMRKGDGVGRSRTMMKNLNFNIADAFGVDGEKSDD

>AtCPK12

MANKPRTRWVLPYKTKNVEDNYFLGQVLGQGQFGTTFLCTHKQTGQKLACKSIPKRKLLCQEDYDDVLREIQIMHHLSEYPNVVRIESAYEDTKNVHLVMELCEGGELFDRIVKRGHYSEREAAKLIKTIVGVVEACHSLGVVHRDLKPENFLFSSSDEDASLKSTDFGLSVFCTPGEAFSELVGSAYYVAPEVLHKHYGPECDVWSAGVILYILLCGFPPFWAESEIGIFRKILQGKLEFEINPWPSISESAKDLIKKMLESNPKKRLTAHQVLCHPWIVDDKVAPDKPLDCAVVSRLKKFSAMNKLKKMALRVIAERLSEEEIGGLKELFKMIDTDKSGTITFEELKDSMRRVGSELMESEIQELLRAADVDESGTIDYGEFLAATIHLNKLEREENLVAAFSFFDKDASGYITIEELQQAWKEFGINDSNLDEMIKDIDQDNDGQIDYGEFVAMMRKGNGTGGGIGRRTMRNSLNFGTTLPDESMNV

>AtCPK13

MGNCCRSPAAVAREDVKSNYSGHDHARKDAAGGKKSAPIRVLSDVPKENIEDRYLLDRELGRGEFGVTYLCIERSSRDLLACKSISKRKLRTAVDIEDVKREVAIMKHLPKSSSIVTLKEACEDDNAVHLVMELCEGGELFDRIVARGHYTERAAAGVTKTIVEVVQLCHKHGVIHRDLKPENFLFANKKENSPLKAIDFGLSIFFKPGEKFSEIVGSPYYMAPEVLKRNYGPEIDIWSAGVILYILLCGVPPFWAESEQGVAQAILRGVIDFKREPWPNISETAKNLVRQMLEPDPKRRLTAKQVLEHPWIQNAKKAPNVPLGDVVKSRLKQFSVMNRFKRKALRVIAEFLSTEEVEDIKVMFNKMDTDNDGIVSIEELKAGLRDFSTQLAESEVQMLIEAVDTKGKGTLDYGEFVAVSLHLQKVANDEHLRKAFSYFDKDGNGYILPQELCDALKEDGGDDCVDVANDIFQEVDTDKDGRISYEEFAAMMKTGTDWRKASRHYSRGRFNSLSIKLMKDGSLNLGNE

>AtCPK14

MGNCCGTAGSLIQDKQKKGFKLPNPFSNEYGNHHDGLKLIVLKEPTGHEIKQKYKLGRELGRGEFGVTYLCTEIETGEIFACKSILKKKLKTSIDIEDVKREVEIMRQMPEHPNIVTLKETYEDDKAVHLVMELCEGGELFDRIVARGHYTERAAASVIKTIIEVVQMCHKHGVMHRDLKPENFLFANKKETASLKAIDFGLSVFFKPGERFNEIVGSPYYMAPEVLRRSYGQEIDIWSAGVILYILLCGVPPFWAETEHGVAKAILKSVIDFKRDPWPKVSDNAKDLIKKMLHPDPRRRLTAQQVLDHPWIQNGKNASNVSLGETVRARLKQFSVMNKLKKRALRVIAEHLSVEETSCIKERFQVMDTSNRGKITITELGIGLQKLGIVVPQDDIQILMDAGDVDKDGYLDVNEFVAISVHIRKLGNDEHLKKAFTFFDKNKSGYIEIEELRDALADDVDTTSEEVVEAIILDVDTNKDGKISYDEFATMMKTGTDWRKASRQYSRDLFKCLSLKLMQDGSLQSNGDTK

>AtCPK15

MGNCCGTAGSLIQDKQKKGFKLPNPFSNEYGNHHDGLKLIVLKEPTGHEIKQKYKLGRELGRGEFGVTYLCTEIETGEIFACKSILKKKLKTSIDIEDVKREVEIMRQMPEHPNIVTLKETYEDDKAVHLVMELCEGGELFDRIVARGHYTERAAASVIKTIIEVVQMCHKHGVMHRDLKPENFLFANKKETASLKAIDFGLSVFFKPGERFNEIVGSPYYMAPEVLRRSYGQEIDIWSAGVILYILLCGVPPFWAETEHGVAKAILKSVIDFKRDPWPKVSDNAKDLIKKMLHPDPRRRLTAQQVLDHPWIQNGKNASNVSLGETVRARLKQFSVMNKLKKRALRVIAEHLSVEETSCIKERFQVMDTSNRGKITITELGIGLQKLGIVVPQDDIQILMDAGDVDKDGYLDVNEFVAISVHIRKLGNDEHLKKAFTFFDKNKSGYIEIEELRDALADDVDTTSEEVVEAIILDVDTNKDGKISYDEFATMMKTGTDWRKASRQYSRDLFKCLSLKLMQDGSLQSNGDTK

>AtCPK16

MGLCFSSAAKSSGHNRSSRNPHPHPPLTVVKSRPPRSPCSFMAVTIQKDHRTQPRRNATAKKTPTRHTPPHGKVREKVISNNGRRHGETIPYGKRVDFGYAKDFDHRYTIGKLLGHGQFGYTYVATDKKTGDRVAVKKIDKAKMTIPIAVEDVKREVKILQALTGHENVVRFYNAFEDKNSVYIVMELCEGGELLDRILARKDSRYSERDAAVVVRQMLKVAAECHLRGLVHRDMKPENFLFKSTEEDSPLKATDFGLSDFIKPGKKFHDIVGSAYYVAPEVLKRRSGPESDVWSIGVISYILLCGRRPFWDKTEDGIFKEVLKNKPDFRRKPWPTISNSAKDFVKKLLVKDPRARLTAAQALSHPWVREGGDASEIPIDISVLNNMRQFVKFSRLKQFALRALATTLDEEELADLRDQFDAIDVDKNGVISLEEMRQALAKDHPWKLKDARVAEILQAIDSNTDGFVDFGEFVAAALHVNQLEEHDSEKWQQRSRAAFEKFDIDGDGFITAEELRMHTGLKGSIEPLLEEADIDNDGKISLQEFRRLLRTASIKSRNVRSPPGYLISRKV

>AtCPK17

MGNCCSHGRDSADNGDALENGASASNAANSTGPTAEASVPQSKHAPPSPPPATKQGPIGPVLGRPMEDVKASYSLGKELGRGQFGVTHLCTQKATGHQFACKTIAKRKLVNKEDIEDVRREVQIMHHLTGQPNIVELKGAYEDKHSVHLVMELCAGGELFDRIIAKGHYSERAAASLLRTIVQIVHTCHSMGVIHRDLKPENFLLLNKDENSPLKATDFGLSVFYKPGEVFKDIVGSAYYIAPEVLKRKYGPEADIWSIGVMLYILLCGVPPFWAESENGIFNAILRGHVDFSSDPWPSISPQAKDLVKKMLNSDPKQRLTAAQVLNHPWIKEDGEAPDVPLDNAVMSRLKQFKAMNNFKKVALRVIAGCLSEEEIMGLKEMFKGMDTDSSGTITLEELRQGLAKQGTRLSEYEVQQLMEAADADGNGTIDYGEFIAATMHINRLDREEHLYSAFQHFDKDNSGYITMEELEQALREFGMNDGRDIKEIISEVDGDNDGRINYDEFVAMMRKGNPDPIPKKRRELSFK

>AtCPK18

MGLCFSSPKATRRGTGSRNPNPDSPTQGKASEKVSNKNKKNTKKIQLRHQGGIPYGKRIDFGYAKDFDNRYTIGKLLGHGQFGFTYVATDNNNGNRVAVKRIDKAKMTQPIEVEDVKREVKILQALGGHENVVGFHNAFEDKTYIYIVMELCDGGELLDRILAKKDSRYTEKDAAVVVRQMLKVAAECHLRGLVHRDMKPENFLFKSTEEGSSLKATDFGLSDFIKPGVKFQDIVGSAYYVAPEVLKRRSGPESDVWSIGVITYILLCGRRPFWDKTQDGIFNEVMRKKPDFREVPWPTISNGAKDFVKKLLVKEPRARLTAAQALSHSWVKEGGEASEVPIDISVLNNMRQFVKFSRLKQIALRALAKTINEDELDDLRDQFDAIDIDKNGSISLEEMRQALAKDVPWKLKDARVAEILQANDSNTDGLVDFTEFVVAALHVNQLEEHDSEKWQQRSRAAFDKFDIDGDGFITPEELRLQTGLKGSIEPLLEEADVDEDGRISINEFRRLLRSASLKSKNVKSPPGTEHIICHNLLDGICIEDTEERTSAVRFEYVSQVL

>AtCPK19

MGCLCINLKKKVKKPTPDISGEQNTEVKSREITPKEQPRQRQPAPRAKFQIVVQPHKLPLPLPQPQEKQKLINHQKQSTLQQPEPILGRPFEDIKEKYSLGRELGRGQFGITYICTEISSGKNFACKSILKRKLIRTKDREDVRREIQIMHYLSGQPNIVEIKGAYEDRQSVHLVMELCEGGELFDKITKRGHYSEKAAAEIIRSVVKVVQICHFMGVIHRDLKPENFLLSSKDEASSMLKATDFGVSVFIEEGKVYEDIVGSAYYVAPEVLKRNYGKAIDIWSAGVILYILLCGNPPFWAETDKGIFEEILRGEIDFESEPWPSISESAKDLVRNMLKYDPKKRFTAAQVLEHPWIREGGEASDKPIDSAVLSRMKQLRAMNKLKKLAFKFIAQNLKEEELKGLKTMFANMDTDKSGTITYDELKSGLEKLGSRLTETEVKQLLEDADVDGNGTIDYIEFISATMNRFRVEREDNLFKAFQHFDKDNSGFISRQELETAMKEYNMGDDIMIKEIISEVDADNDGSINYQEFCNMMKSCSQSHQSKLVQPN

>AtCPK20

MGNTCVGPNLNPNGFLQSVSAAVWRNQKPDDSIKSSKDESSRKKNDKSVNGDDSNGHVSSTVDPAPSTLPTPSTPPPPVKMANEEPPPKPITENKEDPNSKPQKKEAHMKRMASAGLQIDSVLGRKTENLKDIYSVGRKLGQGQFGTTFLCVDKKTGKEFACKTIAKRKLTTPEDVEDVRREIQIMHHLSGHPNVIQIVGAYEDAVAVHVVMEICAGGELFDRIIQRGHYTEKKAAELARIIVGVIEACHSLGVMHRDLKPENFLFVSGDEEAALKTIDFGLSVFFKPGETFTDVVGSPYYVAPEVLRKHYSHECDVWSAGVIIYILLSGVPPFWDETEQGIFEQVLKGDLDFISEPWPSVSESAKDLVRRMLIRDPKKRMTTHEVLCHPWARVDGVALDKPLDSAVLSRLQQFSAMNKLKKIAIKVIAESLSEEEIAGLKEMFKMIDTDNSGHITLEELKKGLDRVGADLKDSEILGLMQAADIDNSGTIDYGEFIAAMVHLNKIEKEDHLFTAFSYFDQDGSGYITRDELQQACKQFGLADVHLDDILREVDKDNDGRIDYSEFVDMMQDTGFGKMGLKVS

>AtCPK21

MGCFSSKHRKTQNDGGEKSIPINPVQTHVVPEHRKPQTPTPKPMTQPIHQQISTPSSNPVSVRDPDTILGKPFEDIRKFYSLGKELGRGQFGITYMCKEIGTGNTYACKSILKRKLISKQDKEDVKREIQIMQYLSGQPNIVEIKGAYEDRQSIHLVMELCAGGELFDRIIAQGHYSERAAAGIIRSIVNVVQICHFMGVVHRDLKPENFLLSSKEENAMLKATDFGLSVFIEEGKVYRDIVGSAYYVAPEVLRRSYGKEIDIWSAGVILYILLSGVPPFWAENEKGIFDEVIKGEIDFVSEPWPSISESAKDLVRKMLTKDPKRRITAAQVLEHPWIKGGEAPDKPIDSAVLSRMKQFRAMNKLKKLALKVIAESLSEEEIKGLKTMFANIDTDKSGTITYEELKTGLTRLGSRLSETEVKQLMEAADVDGNGTIDYYEFISATMHRYKLDRDEHVYKAFQHFDKDNSGHITRDELESAMKEYGMGDEASIKEVISEVDTDNDGRINFEEFCAMMRSGSTQPQGKLLPFH

>AtCPK22

MGNCCGSKPLTASDIVSDQKQETILGKPLEDIKKHYSFGDELGKGKSYACKSIPKRTLSSEEEKEAVKTEIQIMDHVSGQPNIVQIKGSYEDNNSIHIVMELCGGGELFDKIDALVKSHSYYSEKDAAGIFRSIVNAVKICHSLDVVHRDLKPENFLFSSKDENAMLKAIDFGCSVYIKEGKTFERVVGSKYYIAPEVLEGSYGKEIDIWSAGVILYILLSGVPPFQTGIESIIVSTLCIVDAEIKECRLDFESQPWPLISFKAKHLIGKMLTKKPKERISAADVLEHPWMKSEAPDKPIDNVVLSRMKQFRAMNKLKKLALKVIAEGLSEEEIKGLKTMFENMDMDKSGSITYEELKMGLNRHGSKLSETEVKQLMEAVSADVDGNGTIDYIEFISATMHRHRLERDEHLYKAFQYFDKDGSGHITKEEVEIAMKEHGMGDEANAKDLISEFDKNNDGKIDYEEFCTMMRNGILQPQGKLLKRLYMNLEELKTGLTRLGSRLSETEIDKAFQHFDKDNSGHITRDELESAMKEYGMGDEASIKEVISEVDTDNVSCTLQHIANISNIKQVLETL

>AtCPK23

MGCFSSKHRKTQNDGGGERSIPIIPVQTHIVDQVPDHRKPQIPSPSIPISVRDPETILGKPFEDIRKFYSLGRELGRGGLGITYMCKEIGTGNIYACKSILKRKLISELGREDVKTEIQIMQHLSGQPNVVEIKGSYEDRHSVHLVMELCAGGELFDRIIAQGHYSERAAAGTIKSIVDVVQICHLNGVIHRDLKPENFLFSSKEENAMLKVTDFGLSAFIEEGKIYKDVVGSPYYVAPEVLRQSYGKEIDIWSAGVILYILLCGVPPFWADNEEGVFVEILKCKIDFVREPWPSISDSAKDLVEKMLTEDPKRRITAAQVLEHPWIKGGEAPEKPIDSTVLSRMKQFRAMNKLKKLALKVSAVSLSEEEIKGLKTLFANMDTNRSGTITYEQLQTGLSRLRSRLSETEVQQLVEASDVDGNGTIDYYEFISATMHRYKLHHDEHVHKAFQHLDKDKNGHITRDELESAMKEYGMGDEASIKEVISEVDTDNALSPVEIMREVALEIGVPVNTFKQNNVQEDGLYLPVLNNAA

>AtCPK24

MGSCVSSPLKGSPFGKRPVRRRHSSNSRTSSVPRFDSSTNLSRRLIFQPPSRVLPEPIGDGIHLKYDLGKELGRGEFGVTHECIEISTRERFACKRISKEKLRTEIDVEDVRREVEIMRCLPKHPNIVSFKEAFEDKDAVYLVMEICEGGELFDRIVSRGHYTERAAASVAKTILEVVKVCHEHGVIHRDLKPENFLFSNGTETAQLKAIDFGLSIFFKPAQRFNEIVGSPYYMAPEVLRRNYGPEIDVWSAGVILYILLCGVPPFWAETEEGIAHAIVRGNIDFERDPWPKVSHEAKELVKNMLDANPYSRLTVQEVLEHPWIRNAERAPNVNLGDNVRTKIQQFLLMNRFKKKVLRIVADNLPNEEIAAIVQMFQTMDTDKNGHLTFEELRDGLKKIGQVVPDGDVKMLMDAADTDGNGMLSCDEFVTLSIHLKRMGCDEHLQEAFKYFDKNGNGFIELDELKVALCDDKLGHANGNDQWIKDIFFDVDLNKDGRISFDEFKAMMKSGTDWKMASRQYSRALLNALSIKMFKEDFGDNGPKSHSMEFPIARKRAKLLDAPKNKSMELQIS

KTYKPSGLRN

>AtCPK25

MGNVCVHMVNNCVDTKSNSWVRPTDLIMDHPLKPQLQDKPPQPMLMNKDDDKTKLNDTHGDPKLLEGKEKPAQKQTSQGQGGRKCSDEEYKKRAIACANSKRKAHNVRRLMSAGLQAESVLKTKTGHLKEYYNLGSKLGHGQFGTTFVCVEKGTGEEYACKSIPKRKLENEEDVEDVRREIEIMKHLLGQPNVISIKGAYEDSVAVHMVMELCRGGELFDRIVERGHYSERKAAHLAKVILGVVQTCHSLGVMHRDLKPENFLFVNDDEDSPLKAIDFGLSMFLKPGENFTDVVGSPYYIAPEVLNKNYGPEADIWSAGVMIYVLLSGSAPFWGETEEEIFNEVLEGELDLTSDPWPQVSESAKDLIRKMLERNPIQRLTAQQVLCHPWIRDEGNAPDTPLDTTVLSRLKKFSATDKLKKMALRVIAERLSEEEIHELRETFKTIDSGKSGRVTYKELKNGLERFNTNLDNSDINSLMQIPTDVHLEDTVDYNEFIEAIVRLRQIQEEEANDRLESSTKV

>AtCPK26

MGLALFSSDGKLIWKGSTQTGKRRPQEEATMKHSGGNQACYVLGQKTPSIRDLYSLGHKLGQGQFGTTYMCKEISTGREYACKSITKRKLISKEDVEDVRREIQIMHHLAGYKNIVTIKGAYEDPLYVHIVMELCSGGELFDRIIQRGHYSERKAAELIKIIVGVVEACHSLGVMHRDLKPENFLLVNKDDDFSLKAIDFGLSVFFKPGQIFEDVVGSPYYVAPEVLLKHYGPEADVWTAGVILYILVSGVPPFWAETQQGIFDAVLKGHIDFDSDPWPLISDSAKNLIRGMLCSRPSERLTAHQVLRHPWICENGVAPDRALDPAVLSRLKQFSAMNKLKQMALRVIAESLSEEEIAGLKEMFKAMDTDNSGAITFDELKAGLRRYGSTLKDTEIRDLMEAADIDKSGTIDYGEFIAATIHLNKLEREEHLLSAFRYFDKDGSGYITIDELQHACAEQGMSDVFLEDVIKEVDQDNDGRIDYGEFVAMMQKGIVGRTMRKSINMSIRNNAVSQ

>AtCPK27

MGCFSSKELQQSKRTILEKPLVDITKIYILGEELGRGNFGLTRKCVEKSTGKTFACKTILKTKLKDEECEEDVKREIRIMKQLSGEPNIVEFKNAYEDKDSVHIVMEYCGGGELYDKILALYDVGKSYSEKEAAGIIRSIVNVVKNCHYMGVMHRDLKPENFLLTSNDDNATVKVIDFGCSVFIEEGKVYQDLAGSDYYIAPEVLQGNYGKEADIWSAGIILYILLCGKSPFVKEPEGQMFNEIKSLEIDYSEEPWPLRDSRAIHLVKRMLDRNPKERISAAEVLGHPWMKEGEASDKPIDGVVLSRLKRFRDANKFKKVVLKFIAANLSEEEIKGLKTLFTNIDTDKSGNITLEELKTGLTRLGSNLSKTEVEQLMEAADMDGNGTIDIDEFISATMHRYKLDRDEHVYKAFQHFDKDNDGHITKEELEMAMKEDGAGDEGSIKQIIADADTDNDGKINFEEFRTMMRTESSLQPEGELLPIIN

>AtCPK28

MGVCFSAIRVTGASSSRRSSQTKSKAAPTPIDTKASTKRRTGSIPCGKRTDFGYSKDFHDHYTIGKLLGHGQFGYTYVAIHRPNGDRVAVKRLDKSKMVLPIAVEDVKREVQILIALSGHENVVQFHNAFEDDDYVYIVMELCEGGELLDRILSKKGNRYSEKDAAVVVRQMLKVAGECHLHGLVHRDMKPENFLFKSAQLDSPLKATDFGLSDFIKPGKRFHDIVGSAYYVAPEVLKRRSGPESDVWSIGVITYILLCGRRPFWDRTEDGIFKEVLRNKPDFSRKPWATISDSAKDFVKKLLVKDPRARLTAAQALSHAWVREGGNATDIPVDISVLNNLRQFVRYSRLKQFALRALASTLDEAEISDLRDQFDAIDVDKNGVISLEEMRQALAKDLPWKLKDSRVAEILEAIDSNTDGLVDFTEFVAAALHVHQLEEHDSEKWQLRSRAAFEKFDLDKDGYITPEELRMHTGLRGSIDPLLDEADIDRDGKISLHEFRRLLRTASISSQRAPSPAGHRNLR

>AtCPK29

MLQNQHKTTKNQRNKNIGTKYFLRKKIMGFCFSKFGKSQTHEIPISSSSDSSPPHHYQPLPKPTVSQGQTSNPTSNPQPKPKPAPPPPPSTSSGSQIGPILNRPMIDLSALYDLHKELGRGQFGITYKCTDKSNGREYACKSISKRKLIRRKDIEDVRREVMILQHLTGQPNIVEFRGAYEDKDNLHLVMELCSGGELFDRIIKKGSYSEKEAANIFRQIVNVVHVCHFMGVVHRDLKPENFLLVSNEEDSPIKATDFGLSVFIEEGKVYRDIVGSAYYVAPEVLHRNYGKEIDVWSAGVMLYILLSGVPPFWGETEKTIFEAILEGKLDLETSPWPTISESAKDLIRKMLIRDPKKRITAAEALEHPWMTDTKISDKPINSAVLVRMKQFRAMNKLKKLALKVIAENLSEEEIKGLKQTFKNMDTDESGTITFDELRNGLHRLGSKLTESEIKQLMEAADVDKSGTIDYIEFVTATMHRHRLEKEENLIEAFKYFDKDRSGFITRDELKHSMTEYGMGDDATIDEVINDVDTDNDGRINYEEFVAMMRKGTTDSDPKLIR

>AtCPK30

MGNCIACVKFDPDNSKPNQKKKPPRGRQRNPYDDPDGLRTHAPLRVIPMSHQSQISDKYILGRELGRGEFGITYLCTDRETREALACKSISKRKLRTAVDVEDVRREVTIMSTLPEHPNVVKLKATYEDNENVHLVMELCEGGELFDRIVARGHYTERAAATVARTIAEVVRMCHVNGVMHRDLKPENFLFANKKENSALKAIDFGLSVLFKPGERFTEIVGSPYYMAPEVLKRNYGPEVDVWSAGVILYILLCGVPPFWAETEQGVALAILRGVLDFKRDPWSQISESAKSLVKQMLEPDSTKRLTAQQVLDHPWIQNAKKAPNVPLGDIVRSRLKQFSMMNRLKKKALRVIAEHLSIQEVEVIRNMFTLMDDDNDGKISYLELRAGLRKVGSQLGEPEIKLLMEVADVNGNGCLDYGEFVAVIIHLQKMENDEHFRQAFMFFDKDGSGYIESEELREALTDELGEPDNSVIIDIMREVDTDKDGKINYDEFVVMMKAGTDWRKASRQYSRERFKSLSLNLMKDGSMHLHDALTGQSIAV

>AtCPK31

MGCYSSKNLKQSKRTILEKPFVDIGKVYILGDELGQGQFGITRKCVEKTSGKTYACKTILKTNLKSREDEEAVKREIRIMKHLSGEPNIVEFKKAYEDRDSVHIVMEYCGGGELFKKIEALSKDGKSYSEKEAVEIIRPIVNVVKNCHYMGVMLRDLKPENFLLSSTDKNATVKAIDFGCSVFIEEGEVHRKFAGSAYYIAPEVLQGKYGKEADIWSAGIILYILLCGKPPFVTEPEAQMFSEIKSAKIDVDSESWKFIDVKAKHLVNRMLNRNPKERISAAEVLGHPWMKDGEASDKPIDGVVLSRLKQFRDMNKLKKVALKVIAANLSEEEIKGLKTLFTNIDTDKSGTITLEELKTGLTRLGSNLSKTEVEQLMEAADVDGNGTIDIDEFISATMHRYRLDRDDHVYQAFQHFDKDNDGHITKEELEMAMKEHGVGDEVSIKQIITEVDTDNDGKINFEEFRTMMRSGSSLQPQRELLPIK

>AtCPK32

MGNCCGTAGSLAQNDNKPKKGRKKQNPFSIDYGLHHGGGDGGGRPLKLIVLNDPTGREIESKYTLGRELGRGEFGVTYLCTDKETDDVFACKSILKKKLRTAVDIEDVRREVEIMRHMPEHPNVVTLKETYEDEHAVHLVMELCEGGELFDRIVARGHYTERAAAAVTKTIMEVVQVCHKHGVMHRDLKPENFLFGNKKETAPLKAIDFGLSVFFKPGERFNEIVGSPYYMAPEVLKRNYGPEVDIWSAGVILYILLCGVPPFWAETEQGVAQAIIRSVLDFRRDPWPKVSENAKDLIRKMLDPDQKRRLTAQQVLDHPWLQNAKTAPNVSLGETVRARLKQFTVMNKLKKRALRVIAEHLSDEEASGIREGFQIMDTSQRGKINIDELKIGLQKLGHAIPQDDLQILMDAGDIDRDGYLDCDEFIAISVHLRKMGNDEHLKKAFAFFDQNNNGYIEIEELREALSDELGTSEEVVDAIIRDVDTDKDGRISYEEFVTMMKTGTDWRKASRQYSRERFNSISLKLMQDASLQVNGDTR

>AtCPK33

MGNCLAKKYGLVMKPQQNGERSVEIENRRRSTHQDPSKISTGTNQPPPWRNPAKHSGAAAILEKPYEDVKLFYTLSKELGRGQFGVTYLCTEKSTGKRFACKSISKKKLVTKGDKEDMRREIQIMQHLSGQPNIVEFKGAYEDEKAVNLVMELCAGGELFDRILAKGHYSERAAASVCRQIVNVVNICHFMGVMHRDLKPENFLLSSKDEKALIKATDFGLSVFIEEGRVYKDIVGSAYYVAPEVLKRRYGKEIDIWSAGIILYILLSGVPPFWAETEKGIFDAILEGEIDFESQPWPSISNSAKDLVRRMLTQDPKRRISAAEVLKHPWLREGGEASDKPIDSAVLSRMKQFRAMNKLKKLALKVIAENIDTEEIQGLKAMFANIDTDNSGTITYEELKEGLAKLGSRLTEAEVKQLMDAADVDGNGSIDYIEFITATMHRHRLESNENVYKAFQHFDKDGSGYITTDELEAALKEYGMGDDATIKEILSDVDADNDGRINYDEFCAMMRSGNPQQPRLF

>AtCPK34

MGNCCSHGRDSDDNKEEPRPENGGGGVGAAEASVRASKHPPASPPPATKQGPIGPVLGRPMEDVKSSYTLGKELGRGQFGVTHLCTQKATGLQFACKTIAKRKLVNKEDIEDVRREVQIMHHLTGQPNIVELKGAYEDKHSVHLVMELCAGGELFDRIIAKGHYSERAAASLLRTIVQIIHTCHSMGVIHRDLKPENFLLLSKDENSPLKATDFGLSVFYKPGEVFKDIVGSAYYIAPEVLRRKYGPEADIWSIGVMLYILLCGVPPFWAESENGIFNAILSGQVDFSSDPWPVISPQAKDLVRKMLNSDPKQRLTAAQVLNHPWIKEDGEAPDVPLDNAVMSRLKQFKAMNNFKKVALRVIAGCLSEEEIMGLKEMFKGMDTDNSGTITLEELRQGLAKQGTRLSEYEVQQLMEAADADGNGTIDYGEFIAATMHINRLDREEHLYSAFQHFDKDNSGYITTEELEQALREFGMNDGRDIKEIISEVDGDNDGRINYEEFVAMMRKGNPDPNPKKRRELSFK

>AtCRK1

MGICHGKPVEQQSKSLPVSGETNEAPTNSQPPAKSSGFPFYSPSPVPSLFKSSPSVSSSVSSTPLRIFKRPFPPPSPAKHIRAFLARRYGSVKPNEVSIPEGKECEIGLDKSFGFSKQFASHYEIDGEVGRGHFGYTCSAKGKKGSLKGQEVAVKVIPKSKMTTAIAIEDVSREVKMLRALTGHKNLVQFYDAFEDDENVYIVMELCKGGELLDKILQRGGKYSEDDAKKVMVQILSVVAYCHLQGVVHRDLKPENFLFSTKDETSPLKAIDFGLSDYVKPDERLNDIVGSAYYVAPEVLHRTYGTEADMWSIGVIAYILLCGSRPFWARTESGIFRAVLKAEPNFEEAPWPSLSPEAVDFVKRLLNKDYRKRLTAAQALCHPWLVGSHELKIPSDMIIY

KLVKVYIMSTSLRKSALAALAKTLTVPQLAYLREQFTLLGPSKNGYISMQNYKTAILKSSTDAMKDSRVFDFVHMISCLQYKKLDFEEFCASALSVYQLEAMETWEQHARRAYELFEKDGNRPIMIEELASELGLGPSVPVHVVLQDWIRHSDGKLSFLGFVRLLHGVSSRTLQKA

>AtCRK2

MGGCTSKPSSSVKPNPYAPKDAVLQNDDSTPAHPGKSPVRSSPAVKASPFFPFYTPSPARHRRNKSRDGGGGESKSVTSTPLRQLARAFHPPSPARHIRDVLRRRKEKKEAALPAARQQKEEEEREEVGLDKRFGFSKELQSRIELGEEIGRGHFGYTCSAKFKKGELKDQEVAVKVIPKSKMTSAISIEDVRREVKILRALSGHQNLVQFYDAFEDNANVYIVMELCGGGELLDRILARGGKYSEDDAKAVLIQILNVVAFCHLQGVVHRDLKPENFLYTSKEENSMLKVIDFGLSDFVRPDERLNDIVGSAYYVAPEVLHRSYTTEADVWSIGVIAYILLCGSRPFWARTESGIFRAVLKADPSFDEPPWPSLSFEAKDFVKRLLYKDPRKRMTASQALMHPWIAGYKKIDIPFDILIFKQIKAYLRSSSLRKAALMALSKTLTTDELLYLKAQFAHLAPNKNGLITLDSIRLALATNATEAMKESRIPDFLALLNGLQYKGMDFEEFCAASISVHQHESLDCWEQSIRHAYELFEMNGNRVIVIEELASELGVGSSIPVHTILNDWIRHTDGKLSFLGFVKLLHGVSTRQSLAKTR

>AtCRK3

MGQCYGKVNQSKQNGEEEANTTTYVVSGDGNQIQPLTPVNYGRAKNTPARSSNPSPWPSPFPHGSASPLPSGVSPSPARTSTPRRFFRRPFPPPSPAKHIKASLIKRLGVKPKEGPIPEERGTEPEQSLDKSFGYGKNFGAKYELGKEVGRGHFGHTCSGRGKKGDIKDHPIAVKIISKAKMTTAIAIEDVRREVKLLKSLSGHKYLIKYYDACEDANNVYIVMELCDGGELLDRILARGGKYPEDDAKAIVVQILTVVSFCHLQGVVHRDLKPENFLFTSSREDSDLKLIDFGLSDFIRPDERLNDIVGSAYYVAPEVLHRSYSLEADIWSIGVITYILLCGSRPFWARTESGIFRTVLRTEPNYDDVPWPSCSSEGKDFVKRLLNKDYRKRMSAVQALTHPWLRDDSRVIPLDILIYKLVKAYLHATPLRRAALKALAKALTENELVYLRAQFMLLGPNKDGSVSLENFKTALMQNATDAMRESRVPEILHTMESLAYRKMYFEEFCAAAISIHQLEAVDAWEEIATAGFQHFETEGNRVITIEELARELNVGASAYGHLRDWVRSSDGKLSYLGFTKFLHGVTLRAAHARPR

>AtCRK4

MGHCYSRNISAVEDDEIPTGNGEVSNQPSQNHRHASIPQSPVASGTPEVNSYNISPFQSPLPAGVAPSPARTPGRKFKWPFPPPSPAKPIMAALRRRRGAPPQPRDEPIPEDSEDVVDHGGDSGGGERLDKNFGFGKNFEGKYELGKEVGRGHFGHTCWAKAKKGKMKNQTVAVKIISKAKMTSTLSIEDVRREVKLLKALSGHRHMVKFYDVYEDADNVFVVMELCEGGELLDRILARGGRYPEVDAKRILVQILSATAFFHLQGVVHRDLKPENFLFTSRNEDAILKVIDFGLSDFIRYDQRLNDVVGSAYYVAPEVLHRSYSTEADMWSIGVISYILLCGSRPFYGRTESAIFRCVLRANPNFEDMPWPSISPTAKDFVKRLLNKDHRKRMTAAQALAHPWLRDENPGLLLDFSVYKLVKSYIRASPFRRSALKALSKAIPDEELVFLKAQFMLLDPKDGGLSLNCFTMALTRYATDAMMESRLPDILNTMQPLAQKKLDFEEFCAAAVSVYQLEALEEWEQIATSAFEHFEHEGNRIISVQELAGEMSVGPSAYPLLKDWIRSSDGKLSFLGYAKFLHGVTVRSSSSRPR

>AtCRK5

MGLCTSKPNSSNSDQTPARNSPLPASESVKPSSSSVNGEDQCVTTTNNEGKKSPFFPFYSPSPAHYFFSKKTPARSPATNSTNSTPKRFFKRPFPPPSPAKHIRAVLARRHGSVKPNSSAIPEGSEAEGGGVGLDKSFGFSKSFASKYELGDEVGRGHFGYTCAAKFKKGDNKGQQVAVKVIPKAKMTTAIAIEDVRREVKILRALSGHNNLPHFYDAYEDHDNVYIVMELCEGGELLDRILSRGGKYTEEDAKTVMIQILNVVAFCHLQGVVHRDLKPENFLFTSKEDTSQLKAIDFGLSDYVRPGKALRLYAICKLRFQNLETSICLYALTIAFADERLNDIVGSAYYVAPEVLHRSYSTEADIWSVGVIVYILLCGSRPFWARTESGIFRAVLKADPSFDDPPWPLLSSEARDFVKRLLNKDPRKRLTAAQALSHPWIKDSNDAKVPMDILVFKLMRAYLRSSSLRKAALRALSKTLTVDELFYLREQFALLEPSKNGTISLENIKSALMKMATDAMKDSRIPEFLGQLSALQYRRMDFEEFCAAALSVHQLEALDRWEQHARCAYELFEKEGNRPIMIDELASELGLGPSVPVHAVLHDWLRHTDGKLSFLGFVKLLHGVSSRTIKAH

>AtCRK6

MGHCYSRNISTVDDDDEIPSATAQLPHRSHQNHHQTSSSSSIPQSPATSEVNPYNISPFQSPLPAGVAPSPARTPGRKFKWPFPPPSPAKPIMAALRRRRGTAPHPRDGPIPEDSEAGGSGGGIGERLDKNFGFAKNFEGKYELGREVGRGHFGHTCWAKAKKGKIKGQTVAVKIISKSKMTSALSIEDVRREVKLLKALSGHSHMVKFYDVFEDSDNVFVVMELCEGGELLDSILARGGRYPEAEAKRILVQILSATAFFHLQGVVHRDLKPENFLFTSKNEDAVLKVIDFGLSDYARFDQRLNDVVGSAYYVAPEVLHRSYSTEADIWSIGVISYILLCGSRPFYGRTESAIFRCVLRANPNFDDLPWPSISPIAKDFVKRLLNKDHRKRMTAAQALAHPWLRDENPGLLLDFSIYKLVKSYIRASPFRRAALKSLSKAIPEEELVFLKAQFMLLEPEDGGLHLHNFTTALTRYATDAMIESRLPDILNMMQPLAHKKLDFEEFCAASVSVYQLEALEEWEQIATVAFEHFESEGSRAISVQELAEEMSLGPNAYPLLKDWIRSLDGKLNFLGYAKFLHGVTVRSSSSRPMR

>AtCRK7

MGLCHGKPIEQQSKNLPISNEIEETPKNSSQKAKSSGFPFYSPSPLPSLFKTSPAVSSSSVSSTPLRIFKRPFPPPSPAKHIRALLARRHGSVKPNEASIPEGSECEVGLDKKFGFSKQFASHYEIDGEVGRGHFGYTCSAKGKKGSLKGQDVAVKVIPKSKMTTAIAIEDVRREVKILRALTGHKNLVQFYDAFEDDENVYIVMELCQGGELLDKILQRGGKYSEVDAKKVMIQILSVVAYCHLQGVVHRDLKPENFLFTTKDESSPLKAIDFGLSDYVRPDERLNDIVGSAYYVAPEVLHRTYGTEADMWSIGVIAYILLCGSRPFWARSESGIFRAVLKAEPNFEEAPWPSLSPDAVDFVKRLLNKDYRKRLTAAQALCHPWLVGSHELKIPSDMIIYKLVKVYIMSSSLRKSALAALAKTLTVPQLTYLQEQFNLLGPSKNGYISMQNYKTAILKSSTEATKDSRVLDFVHMISCLQYKKLDFEEFCASALSVYQLEAMETWEQHARRAYELYEKDGNRVIMIEELATELGLGPSVPVHVVLQDWIRHSDGKLSFLGFVRLLHGVSSRTLQKA

>AtCRK8

MGGCTSKPSTSSGRPNPFAPGNDYPQIDDFAPDHPGKSPIPTPSAAKASPFFPFYTPSPARHRRNKSRDVGGGGESKSLTSTPLRQLRRAFHPPSPAKHIRAALRRRKGKKEAALSGVTQLTTEVPQREEEEEVGLDKRFGFSKEFHSRVELGEEIGRGHFGYTCSAKFKKGELKGQVVAVKIIPKSKMTTAIAIEDVRREVKILQALSGHKNLVQFYDAFEDNANVYIAMELCEGGELLDRILARGGKYSENDAKPVIIQILNVVAFCHFQGVVHRDLKPENFLYTSKEENSQLKAIDFGLSDFVRPDERLNDIVGSAYYVAPEVLHRSYTTEADVWSIGVIAYILLCGSRPFWARTESGIFRAVLKADPSFDEPPWPFLSSDAKDFVKRLLFKDPRRRMSASQALMHPWIRAYNTDMNIPFDILIFRQMKAYLRSSSLRKAALRALSKTLIKDEILYLKTQFSLLAPNKDGLITMDTIRMALASNATEAMKESRIPEFLALLNGLQYRGMDFEEFCAAAINVHQHESLDCWEQSIRHAYELFDKNGNRAIVIEELASELGVGPSIPVHSVLHDWIRHTDGKLSFFGFVKLLHGVSVRASGKTTR

>SlCDPK1

MGNTCVGPSISKNGIFQSVSAAMWRSRSPDDTASTVTNGESGRVETPTSAKETESPLPVQDKPPEPMTMPKLEEKEEEKPKKPKKPAEMKRVSSAGLRTDSVLQKKTGNLKEFFSIGKKLGQGQFGTTFLCTEKATGKRYACKSIAKRKLLTDDDVEDVRREVQIMHHLAGHPHVISIKGAYEDAVAVHLVMEYCAGGELFDRIIQRGHYTERKAAELTRTIVGVVEACHSLGVMHRDLKPENFLFVDQKEESLLKTIDFGLSIFFKPGDKFTDVVGSPYYVAPEVLRKRYGPEADVWSAGVIIYILLSGVPPFWAENEQGIFEQVLHGDLDFTTDPWPSISEDAKDLMRRMLIRDPRKRLTAHEVLCHPWVQVDGVAPDKPLDSAVLSRMKQFSAMNKLKKMALRVIAESLSEEEIAGLKEMFRMIDTDNSGQITFEELKDGLKRFGSNLKESEIYDLMQAADVDNSGTIDYGEFIAATLHLNKIERDDHLFAAFSYFDKDGSGYITADELQHACEEFGIGDVRMEEMIREADQDNDGRIDYNEFVAMMQKGNPVLGGGKKGLEQSFSIGFREALKL

>SlCDPK2

MGNTCVGPSISRNRFLQSVSAAMWPARVPDDSGSTTNGGSSRGGTVESGKDPDLVVQNRAPEQMTMPKSEQKEVEPVKKEEEPVKPKKAVEMKRVGSAGLKTDSVLQKKTGNLKEFFSIGKKLGQGQFGTTFKCVEKGTGKEYACKSIAKRKLLTDDDVEDVRREIQIMHHLAGHPNVISIKGAYEDAVAVHVVMERCAGGELFDRIIQRGHYTERKAAELTRTIFGVVEACHSLSVMHRDLKPENFLFVDQKEDSLLKTIDFGLSVFFKPGERFTDVVGSPYYVAPEVLKKRYGPEADVWSAGVIVYILLSGVPPFWAENEQGIFEQVLHGDLDFSSDPWPSISEDAKDLVRGMLVRDPKKRLTAHEVLCHRWVQVDGVAPDKPLDSAVLSRMKQFSAMNKLKKMALRVIAESLSEEEIAGLKEMFKMIDTDNSGQITFEELKVGLKRVGANLKESEIYDLMQAADVDNSGTIDYGEFIAATLHFNKIEREDHLFAAFSYFDKDGSGYITADELQQACEEFGIGDVHLEDMIRDADQDNDGRIDYNEFVAMMQKGHPAS

GVGKKGLEHSFSTGFRDVLKL

>SlCDPK3

MLSAGLQVESVLKTKTGLLKEHYDLGAKLGHGQFGTTFLCVEKATGEKYACKSIAKRKLLTPEDVEDVRREIQIMHHLSGNPNVISIKAAYEDGVAVHVVMELCSGGELFDRIVKQGHYTERQAAELARTIVGVVEACHALGVLHLDLKPENFLFVNEKEDSPLKIIDFGLSMFFKPGQIFSDIVGSPYYVAPEVLQKRYGQEADIWSAGVIIYILLTGVPPFWGESEQEIFDEVLRANIDFTSDPWPNISGDAKDLVKKMLVRDPKQRLTAHEVLCHPWVKIDGVAPDKPLDSAVLSRLTQFSAMNKLKKMALMVIAESLSEEEIAGLKEMFKMIDTDDSGHITLDELKVGLKQFGADLSETEIRDLMKAADVDNSGTIDYGEFIAAMLHVNKAEKEDYLSAAFSYFDKDGSGYITADELQKACEEFGMKDVRLEEIIQEVDQDNDGRIDYKEFVAMMQKGNANFGNRRLPNNFSIGFRDATKKCIVESKHRENASSY

>SlCDPK4

MGNNCVHAKISKDGFFSSSWWSRSPEMIAYEKKESSFQEGVDVVQSNPPELAKIESRKSDVIGTEQVMIVVTDEKKDAWMIKSEEMITITVDLKQEKTSNAKTKKKPHNVKRMASAGLQVDSVLKTKTGHLKEHYNLGEKLGHGQFGTTFLCIEKGTGKKYACKSIAKRKLLTDEDVDDVRREIQIMHHLSGHQNVISIKGAYEDAVAVHVVMELCTGGELFDRIIKRGHYSERQAAELARTILGVVEACHSLGVMHRDLKPENFLFVNEEEDSPLKTIDFGLSMFFKPGQIFDDVVGSPYYVAPEVLRKRYGPEADIWSAGVIIYILLSGVPPFWGESEEEIFDEVLHGDIDFDLDPWPKISQGAKDLVRRMLIRDPKKRLTAHEVLCHPWVQIDGVAPDKPLDSAIFTRLTQFSAMNKLKKMAIRVIAERLSEEEIAGLKEMFKMIDTDNSGQITFDELKIGLKKFGTNLNESEIRDLMKAADIDNSGTIDYGEFVAAMLHANKIEKEDYLFAAFSYFDKDGSGYITADELQKACEEFGIEDVHLEEIIQEADQDNDGRIDYNEFVAMMHKGNADLGKKRLPNNFNIGYREPMLAC

>SlCDPK5

MGNTCIGPKLGNNGFLQSVTAAVWKTRQQQHLPLANKGDSNSHKTQEHSSVGSSLNVDGNDANRFGGTQSTPPPHLKISGNDTNEKNSSPNVSNKPVEGVKQNKPSHVKRVSSIGLKIDSVLGRKTGNLKEIYSLGRKLGQGQFGTTYLCVDKVHGREMACKSIAKRKLNTEEDVEDVRREIQIMHHLAGHPSVVQIVGAYEDAVEVHVVMELCAGGELFDRILQRGHYSEKKAAELARVIVGVVEACHSMGVMHRDLKPENFLFINQDEDSHLKTIDFGLSVFFKPGEIFTDVVGSPYYVAPEVLRKHYGSECDIWSAGVIIYILLSGVPPFWEETEQGIFEQVLRGELDFVSEPWPSISESAKDLVRKMLVRDPKKRLTAHEVLCHPWVRVGGVAPDKPLDSAVLTRLNQFSAMNKLKKIAVRVIAESLSAEEIAGLKEMFKMIDTDNSGNITLEELKKGLERVGADLKDSEITSLMQAADTDNSGTIDYGEFIAAMLHLNKIQKEDHMYAAFSYFDQDGSGYITKDELQQACEKFGMSNIPIEELMREVDQDNDGRIDYNEFVAMMQDTGLGELGSRRR

>SlCDPK6

MGNTCSGPTLNKDSADSSKTDGKGSDSNNVQNTPPPHLQIPGDQDEKSKNSQNGDDIVVEDVKRNKSSNLKRVMSAGLQVDTVLGRKTGNLKDIYTLGRKLGQGQFGTTFLCVDKAQPKEFACKSIAKRKLTTEEDVEDVRREIQIMHHLAGHPSVVQIVGAYEDAVAVHVVMELCAGGELFDRIIKREESSLKTIDFGLSVFFRPGETFTDVVGSPYYVAPEVLRKRYGPECDIWSAGVIIYILLSGVPPFWDETEQGIFEQIVKGELDLVSEPWPAISESAKDLVRKMLVREPKKRLTAHEVLCHPWVRVGGVAPDKP

LDSAVLSRLNQFSAMNKLKKIAIRVIAENLSGEEIAGLKQMFKMIDADNSGHITLEELKKGLEKVGSKLKDSEINSLMQAADMDNSGTIDYGEFIAAMLHLNKVQKEDHMYAAFSYFDQDGSGYITQEELQKACEKFGLSNIPMEELMREVDQDNDGRIDYNEFVAMMQDTGFGKNGNKRV

>SlCDPK7

MGNNCVGPKLANNGFLQSVTAAVWKPNQSENLPLPNAGEPNSENSVDSSKGAVDGSGIQGNPPPHLMINADTQMGNNDVVIANNVTVTDKPPVEGVKQNKPTHVRRTSSIGVQIESVLGRKTANLKEIYSLGRKLGQGQFGTTFLCLGKSCGKQYACKSIAKRRLTTEEDVEDVRREIQIMHHLAGQPSVVQIVGAYEDAVAVHVVMELCAGGELFDRILQRGHYSEKKAAELARVIVGVVEACHSLGVMHRDLKPENFLFADQEEDSTLKAIDFGLSVFFKPGEIFNDVVGSPYYVAPEVLRKHYGLECDIWSAGVIIYILLSGVPPFWDETEQGIFEQVLTGELDFASEPWPAISESAKDLVRKMLVRDPKMRLTAHEVLCHPWVRVGGVAPDKPLDCAVLSRLNQFSAMNKLKKIAIRVIAESLSGEEIAGLKEMFKMIDADNSGHITLEELKTGLEKVGANLNDSEIVSLMQAADVDNSGTIDYGEFIAAMLHLNKIQKEDHMYAAFSYFDEDGSGYITQDELQKACDKFGLSNIPIEELMREVDQDNDGRIDYSEFVAMMQDTGFGDKGSKSVL

>SlCDPK8

MDSSDLTKTKTSSLSTPSKHFWVLPYKTQSLESLYTLGKILGQGQFGTTYLCTEISTSNLYACKTIPKKKLICKEDYEDVWREIQIMHHLSEHPNVVRIKGTYENALYVHIVMELCAGGELFDRIVEKGQYSEKEAAKLIKTIVGVVEACHSLGVMHRDLKPENFLFLSSQEDAALKATDFGLSVFYKPGETFSDVVGSPYYVAPEVLCKHYGPESDVWSAGVILYILLSGVPPFWAETDMGIFRQILRGKLDLESEPWPGISDSAKDLIRKILDRNPKRRLTAHEVLCHPWIVDDSMTPDKPLDSAVLSRLKQFSAMNKLKKMALRVIAERLSEEEIGGLKELFKMLDTDNSGTITFEELKEGLRRVGSELMESEIKDLMDAADIDNSGTIDYGEFIAATVHLNKLEREENLLSAFSFFDKDGSGYITIEELQQACKEFGLSELNLDEIIKDIDQDNDGQIDYGEFSAMMRKGTGGGVGRRTIRNTLNLGEALGLVQSEENV

>SlCDPK9

MASETDRKAATESTKTSTVPPLKPTWVLPYRTERLQQLYSIGKKLGQGQFGTTHLCTEKSTTTLYACKTIPKKKLICKEDYEDVWREIQIMHHLSEHPNVVRIKGTYEDTLYVHIVMELCAGGELFDRIVEKGHYSEREAAKLIKTIVGVVEGCHSLGVMHRDLKPENFLFLSSDEDAALKATDFGLSVFYKPGEMFSDVVGSPYYVAPEVLRKHYGPESDVWSAGVILYILLSGVPPFWAETEMGIFRQILQAKLDFESEPWPGISDSAKDLIRKMLDRNPKRRLTAHEVLCHPWIVDDTIAPDKPLDSAVLSRLKQFSAMNKLKKMALRVIAERLSEEEIGGLKELFKMIDTDNSGTISFDELKEGLRRVGSELMESEIKDLMDAADIDNSGTIDYGEFLAATVHLNKFEREENLVSAFSFFDKDGSGYITIDELQHACKEFGLSELNLDEMIKDIDQDNDGQIDYGEFAAMMRKGNSGGVGRRTMRNTLNLGEALGLVESKEEDV

>SlCDPK10

MEIPKSENSKPPTVSSSTKSCMNVLPYQTPRIGEHYTLGKKLGQGQFGTTYLCTENATGLEYACKTIPKRKLFCKEDYEDVWREIQIMHHLSEHPYVVRIKGTYEDNLFVHIVMEVCKGGELFDRIVQKGHFSEKKAAQLMKTIVKVVEACHSLGVMHRDLKPENFLFDSSDEDAKLKATDFGLSIFYKPGQYFSDVVGSPYYVAPEVLHKYYGPEIDVWSAGVILYILLCGVPPFWAETDNGIFKQILKGKIDFESEPWPQISDSAKDLVKKMLTRDPRARLTAHQVLCHPWIVDDNVAPDRPLGSAVLSRLKQFYDMNKLKKMALRVIAERLSEEEIGGLKQLFKMIDTDNSGTITYEELKHGLKRVGSDLTESEIKALMSAADFDNNGTIDYGEFIAATLHLNKMEREENLLAAFSYFDKDGSGYITIDELQQACQEFGLGDVKLEDIIKEIDIDNDGRIDYGEFATMMKKGNTGLAARTMRGNLTFNLADALGASDSDNGQ

>SlCDPK11

MAQVVAKKRPPISSKPSPNVLPYQTPRLREHYTLGKKLGQGQFGTTYQCTEKATGLQYACKSIPKRKLLCREDYEDVWREIQIMHHLSEHPNVVRIKGTYEDNLFVHLVMELCKGGELFDRIVQKGHYSERQAAHLMKTIVKVVEACHSLGVMHRDLKPENFLFDSSDEDATLKATDFGLSIFYKPGQYVSDVVGSPYYVAPEVLHKFYGPEIDVWSAGVILYILLSGVPPFWAETDNGIFKQILKGKIDFESEPWPHISDSAKDLVKKMLDKDPKARITAHEVLCHPWLVDDAAAPDKPLGSAVLNRLKQFYDMNKLKKMALRVIAERLSEEEIGGLKQLFKMIDTDSSGTITYEELKDGLKRVGSDLGESDIKALMKAADFDNSGTIDYGEFIAATLHLNKMEREENLLAAFSYFDKDGSGYITTDELQQACVEFGLGDVKLDDIIKEIDIDNDGRIDYGEFATMMKKGNTGFAARTMRGNLNFNLADALGASDNQADRIRWTNQ

>SlCDPK12

MGNTCRGSIGGKTFQGYNQPEDSSCSTNHNPSSGNSYSSSDNFSPTSNAQQNSNHKKEHSLSLVSPRKASMNRSGSNQAYYVMGHKTQNIRDLYTLGRKLGQGQFGTTYLCTEISTGAEYACKSISKRKLISKEDIDDVRREIQIMHHLAGHRNIVTIKGAYEDPLYVHIVMEICSGGELFDRIIQRGHYSERKAAELTKIIVGVVEACHSLGVMHRDLKPENFLLVNKDDDFSLKAIDFGLSIFFKPGQIFTDVVGSPYYVAPEVLLKHYGPEADVWTAGVILYILLSGVPPFWAETQQGIFDAVLKGHIDFDSDPWPLISESAKDLIRKMLCMQPSERLTAHEVLCHPWICENGVAPDRALDPAVLSRLKQFSAMNKLKKMALRVIAESLSEEEIAGLREMFKAMDTDNSGAITFDELKAGLRKYGSTLKDTEIRELMDAADVDNSGTIDYGEFIAATVHLNKLEREEHLMAAFQYFDKDGSGYITVDEVQQACIEHNMTDVYFEDIIREVDQDNDGRIDYGEFVAMMQKGNPCIGRRTMRNSLNLSMRDASGAQ

>SlCDPK13

MGNACRGSFGGKTFQGYPQPQDHSESNSNPKHNSDSPNPKKEQQPLVTMNRTSTNQSYYVLGHKTPNIRDLYTLGRKLGQGQFGTTYLCTELSSGIDYACKSIAKRKLISKEDVEDVRREIQIMHHLAGHKNIVSIKGAYEDPLYVHIVMELCGGGELFDRIIQRGHYTERKAADLTKIIVGVVEACHSLGVMHRDLKPENFLLVNKDDDFSLKAIDFGLSVFFKPGQIFTDVVGSPYYVAPEVLLKHYGPEADVWTAGVILYILLSGVPPFWAETQQGIFDAVLKGHIDFDSDPWPLISESAKDLIRKMLCMRPPERLTAHEVLCHPWICENGVAPDRALDPAVLSRLKHFSAMNKLKKMALRVIAESLSEEEIAGLKEMFKAMDTDNSGAITFDELKAGLRKYGSTLKDIEIRELMDADVDNSGTIDYGEFIAATIHLNKLDREEHLMAAFQYFDKDGSGYITVDELQQACADHNITDVFFEDIIREVDQDNDGRIDYGEFVAMMQKGNPCIGRRTMRNSLNFSMRDAPGAH

>SlCDPK14

MGNCCSSGEEQQPNETTNNNNNNEINNPKNVENNSSTQHNNLDQSSTTPTPTKTSPSPSSKPSKQSPIGLVLGRPMEDVRSTYTIGKELGRGQFGVTHLCTQKQTNEQYACKTIAKRKLVSKEDIEDVRREVQIMHHLTGQENIVQLKGAYEDKHSVHLVMELCAGGELFDRIIAKGHYTERAAASLLRTIVQIVDTCHSMGVIHRDLKPENFLLLSKDENAPLKATDFGLSVFYKQGDVFKDIVGSAYYIAPEVLKRRYGPEVDIWSIGVMLYILLCGVPPFWAENENGIFNAILRGHIDFSSDPWPSISSGAKDLVRKMLTVDPRQRLTAMQVLNHSWIKEDGEAPDTPLDNAVLHRLKQFRAMNKFKKVALRVIAGCLSEEEIMGLKQMFKNMDTDNSGTITLEELKQGLAKQGTKLSDYEIKQLMEAADVDGNGTIDYEEFITATVHMNKMDREEHLYTAFQYFDKDHSGYISREELEQALREFGMDDENDLREIINEVDTDHDGRINYDEFVAMMKKGNPEAATMNPRKRRDSFVA

>SlCDPK15

MGGCCSKAETDPAQNNEEIGQSYSKQGESNAGNNDMQGSTTPSKAPPHASPNHSSKPSKAAPIGPVLGRPMEDIKATYTLGKELGRGQFGVTHLCTHKQTGEQFACKTIAKRKLVNKEDIEDVRREVQIMHHLTEQPNIVELKGAYEDKHSVHLVMELCAGGELFDRIIAKGHYTERAAASLLRTIVQIVHTCHSMGVIHRDLKPENFLLLSKDEDAPLKATDFGLSVFYKQGDVFKDIVGSAYYIAPEVLKRRYGPEVDIWSIGVMLYILLCGVPPFWAESENGIFNAILRGHVDFSSDPWPSISSGAKDLVRKMLNSDPSQRLTALQVLNHSWIKEDGEAPDTPLDNAVLNKLKNFSAMNKFKKVALRVIAGCLSEEEIMGLKQMFRGIDTDNSGTITLEELKQGLAKQGNKLSDYEIKQLMESADADGNGTIDYEEFITATMQMNKMDREEHLYKAFQYFDKDSSGYITMEELEQALIEFGMNDAKDIKEIISEVDSDNDGRINYDEFVAMMKKGNPEVATNAKKRRDVFVE

>SlCDPK16

MGNCCSRGQPNNDDDHHIMSKEKSNDNDQSKSCNNNNNTTPPKSPDPSSKPSKKSPIGPVLGRPMEDVKKTYSIGKELGRGQFGVTHLCTHKQNGEQFACKTIAKRKLVNKEDIEDVKREVQIMHHLTGQQNIVELKGAYEDKHSVHLVMELCAGGELFDRIITKGHYTERAAATLLRTIVQIVHTCHSMGVIHRDLKPENFLLLNKDEDSPLKATDFGLSVFYKQGDVFKDIVGSAYYIAPEVLKRRYGPEVDIWSVGVMLYILLSGVPPFWAETEHGIFNAILRGHIDFSSDPWPSISHGAKDIVRKMLTSDPKQRLT

AIQVLNHPWIKEDGDAPDTPLDNAVLSRLKQFRAMNNFKKVALRVIAGCLSEEEIMGLKQMFKSMDADNSGAITLEELKQGLAKQGTKLSDYEIQQLMEAADADGNGTIDYEEFITATMHMNRMDKEEHLYTAFQYFDKDNSGYITIEELEQALREFGIDDGKDIQDIVAEVDSNNDGRINYDEFAAMMRKGSPETTANIKKRRESFVV

>SlCDPK17

MGNCNSLSSDQATTTSGGGGGGGAPSTPSTTGIRIIPSSQPPPPRPLSGIGRVLGRPMEDVRSIYIFGGELGRGQFGVTYLVTNKKTRQQFACKSIATRKLINKDDVDDVRREVQIMHHLTGHRNIVELKGTFEDKHHVHLVMELCAGGELFDRIIAKGHYSERAAAGVCRQMVTVVHNCHSMGVMHRDLKPENFLFLSSDETSPLKATDFGLSVFFKPGDVFKDLVGSAYYVAPEVLRREYGPEADIWSAGVILYILLSGVPPFYGENDQSIFDAVLRGHLDFSSDPWPSVSSSAKDLVKKMLRSDPRERISAAEVLNHPWMREDGDASDKPLDIAVLSRMKQFRAMNKLKKVALKVIAENLSEEEIIGLKEMFKSIDTDDSGTITFEELKAGLTKMGTKLSESEVRQLMEAADVDGNGTIDYLEFITATMHMNRMEREDHLYKAFEYFDKDKSGYITMEELEHALKEYNITDDKTIKEIIAEVDTDNDGRINYDEFAAMMRKGNPDFVNNRRRR

>SlCDPK18

MGLCFTKECCCPHYRDVPISSSPEYNPIPVSSQKYPQNSIPEPTPAPAPASTFMPNQIGPILGKPYVDIKTLYDLDKELGRGQFGITYLCSDKSSGLKYACKSISRRKLVTQKDIEDVRREVTILQYLSGQPNIVEFKGAYEDKNNLHLVMELCSGGELFDRITAKGNYSEKEAARIGRQIVNVVHVCHFMGVMHRDLKPENFLMVSKDDDSPLKATDFGLSVFIEEGKIYKDIVGSAYYVAPEVLKRKYGKEIDVWSAGVILYILLSGFPPFWAETEKGIFEEIVKGQLDFESSPWPSISTSAKDLVRKMLTMDPKKRITADEALQHPWLMKDGEASDKPIDSAVLSRLKQFRAMNKMKKLALKVIAENLSEDEIKGLKQMFNNMDTDGSGTITYEELKTGLSRLGSKLTESEIKQLMDAADVDNSGTIDYIEFITATMHRHKLEREENLYKAFQFFDKDSSGFITRDELRHAMEEYGMGDEATIDEILDDVDTNKDGLINYDEFVAMMRRGTVDEVKPR

>SlCDPK19

MGICASKGKPNNANNGHHGSGSGGVPIHRNEIQYTKSPGPEAQLHVRPPPSPKPAVRYDTILGKPYEDVKLHYTLGKELGRGQFGVTYLCTEIATGQQYACKSISKKKLVTKADKDDMRREIKIMQHLSGQPNIVEFKGAYEDKGSVYLVMELCGGGELFDRIIAKGHYSERAAATMCRAIVNVVHVCNFMGVLHRDLKPENFLLSDKSENAALKITDFGLSVFIQEGKSYKDIVGSAYYVAPEVLRRCYGKEIDIWSAGVMLYILLSGVPPFWAETEKGIFDAILKGTIDFESKPWPSVSSSAKDLVQKMLTKDPKKRI

TAAQVLEHPWLKEGGVASDKPLEGAVLSRMKQFRAMNKLKKLALKVIAENLSAEEIHGLKAMFHNIDTDNSGTITYEELKSGLAKLGSKLTEAEVKQLMEAADVDGNGSIDYTEFITATMHKHRLERDENLYTAFQYFDKDSSGFITRDELEAAMEEHGIGDPSCIREIISEVDTDNDGRINYEEFCTMMRSGAKQPGKVF

>SlCDPK20

MGGCFSKNRYLEGDGKGTSNRPGRRRRGNTGVASYQHYKHPVYYHPRPTAQQRHAHQVTVKSSQPGTILGKPYENIRAHYTLGRELGRGQYGVIYFCTENSTRRNYACKSILKRKLVSRKDTEDIKREIQMMQHLSGQPNIVEFKGAYEDHYSVHLVMELCAGGELFDRIIARGYYSEKDAADIIRQIVNVVYICHFMGVMHRDLKPENFLLTSKDEHTMIKATDFGLSVFIEQGKVYRDIVGSAYYVAPEVLRRSYGKEADVWSAGVILYILLSGVPPFWDETEKGIFHAILQGEIDFHSDPWPSISNSAKDLVRKMLTQDAKSRITSAEVLEHPWLQSGEASDKPIDSAVLSRMKQFRAMNKLKKLALKVIAENLSEEEIKGLKAMFANMDTDNSGTI

TYEELKSGLARLGSKLSEAEVKQLMEAVS

>SlCDPK21

MGGCFSKKYTQQDANGHRAGRRVNQAYQKPPQPQPERPYQPQPQQERPYQPPPQPAYQPPPQPKPQPQPHPVPVTVQSGQPQDQMQGPHMNNILGKPFEEIRKLYTLGKELGRGQFGVTYYCTENSTGNPYACKSILKRKLVSKNDREDMKREIQIMQHLSGQPNIVEFKGAYEDRQSVHLVMELCAGGELFDRIIARGYYSEKDAAEIIRQIVNVVNICHFMGVMHRDLKPENFLLTSKDENAMLKATDFGLSVFIEEGKVYRDIVGSAYYVAPEVLRRSYGKEADVWSAGVILYILLSGVPPFWAETEKGIFNTILKG

EIDFQSDPWPSISNSAKDLIRKMLTQEPRKRITSAQVLEHPWLRLGEASDKPIDSAVLSRMKQFRAMNKLKKLALKVIAENLSEEEIKGLKAMFHNIDTDNSGTITYEELKSGLARLGSKLTETEVKQLMEAADVDGNGSIDYIEFITATMHRHRLERDEHLFKAFQHFDKDHSGFITRDELENAMKEYGMGDEATIKEIIAEVDTDNDGRINYEEFCAMMRSGTTQPQQKLF

>SlCDPK22

MGNCNACIRPEEASKTDPQTKPKKPRERRPNPYSGSPAPIRVLKDFIPKTRISDKYILGRELGRGEFGVTYLCTDRETREALACKSISKKKLRTAVDIEDVRREVAIMSSLPDHPNIVKLRATYEDNEAVHLVMELCEGGELFDRIVARGHYSERAAAGVARTVAEVVRMCHANGVMHRDLKPENFLFANKKEHSALKAIDFGLSVFFKPGERFSEIVGSPYYMAPEVLKRNYGPEVDIWSAGVILYILLCGVPPFWAETEQGVALSILRGVIDFKREPWPQVSEKAKSLVRQMLEPDPKKRLTAQQVLDHPWIQNAKKASNVPLGDIVRTRLKQFSIMNRFKKKALRVIAEHLKLEEIEVIREMFALMDSDGDGKITYDELKTGLRKVGSQLAEAEMKLLMDVADVDGNGVLDYGEFVAVIIHLQRMENDEHFRRAFMFFDKDGSGYIELDELREALADESGACDTDVVNEIMREVDTDKDGQISFEEFVGMMKAGTDWRKASRQYSRERFKSLSVNLMKDGSLQLQDVLSGQTVIV

>SlCDPK23

MGNCCRSPAAVAREDVKSSNYSGNDHGRKDKYSAGNKQKQITVLTDVKKENVEERYLVDRELGRGEFGITYLCIDRSSRELLACKSISKRKLRTAVDVEDVRREVAIMKHLPQNSSIVSFKEACEDENAVHLVMELCEGGELFDRIVARGHYTERAAAAVTRTIVEVVQLCHKHGVIHRDLKPENFLFANKKENSPLKAIDFGLSIFFKPGEKFSEIVGSPYYMAPEVLKRNYGPEIDIWSAGVILYILLCGVPPFWAESEQGVAQAILRGAIDFKREPWPSISEGAKNLVRQMLEADPKLRLSAKQVLEHPWLQNAKKAPNVPLGDVVKSRLKQFSMMNRFKRKALRVIADFLSNEEVGDLKEMFNKIDTNNDGIVSVEELKAGLKLNSQLAESEVQMLIEAIDTNGKGTLDYGEFIAISLHLQRMANDEHLHKAFSYFDKDGNGYIEPDELRDALMEDGADDCTNVANDIFQEVDTDKDGRISFEEFAAMMKTGTDWRKASRHYSRGRFNSLSVKLMKDGSINLGNE

>SlCDPK24

MGTCMSVQNASFLKRTRMRPTPIDQETCSKSASRTSVPKSQKFLRPINVVNDPSGDDIYQRYEFGKELGRGEFGITYQCVDKTSGENVACKTIAKSKLRTEIDVEDVRREVVIMRHLPKHPNIVSYKEVYEDKDAVYLVMELCEGGELFDRIVARGHYTERAAALVTKTILEVVQVCHKHGVIHRDLKPENFLYANVNENAQLKAIDFGLSIFFEPGQRFGEIVGSPYYMAPEVLRRNYGPEVDVWSAGVILYILLCGVPPFWAETEEGIAHAIVKGTIDFNRDPWPRVSDEAKDLVKGMLDANPYNRFTVEEVLDHHWIQNADKVSNVCLGEGVRTKIKQFTLMNKFKKKVLRVVADNLPLDQVHGIKQMFYMMDTDKNGNLSFQELKDGLHMMGQTVAEPEVHLLMDAADVDGNGMLNCEEFVTMAVHLQRLSNDDHLKQAFLQFDKNKSGFIEYEDLKISLFDDSLAPQNDQVINDIIFDADLDKDGRISYQDFKVMMSTGTDWKMGSRQYSKAMLNALSMRLFKDKSMQLTN

>SlCDPK25

MGNCCAVPKTSETEEKKRGKNKPNPFSVDYGHHGNGHKSYVLDNPTGTDIEATYELGRELGRGEFGVTYLSTDKVSGEVYACKSISKKKLRTRVDIEDVRREVEIMKHLPKHPNIVTLKDTYEDDNAVHIVMELCEGGELFDRIVARGHYTERAAAAVTRTIVEVIQMCHKHGVMHRDLKPENFLFENKKETAPLKAIDFGLSVFFKPGERFNEIVGSPYYMAPEVLKRDYGPEVDVWSAGVILYILLCGVPPFWAETEQGVAQAIIRSVVNFKRDPWPKVSDNAKDLVKKMLNPDPSQRLTAQEVLDHPWIQNAKKAPNVSLGETVKARLKQFSMMNKLKKRALRVIAEHLTVDEVAGIKEGFQLMDISNKGKIDVNELRVGLQKLGHQIPESDVQILMDVGDVDKDGFLDYGEFVAISVHLRKMANEEHLKKAFDFFDRNQNGYIEIEELREALDDEIETNSEEVINAIMQDVDTDKDGRISYDEFSTMMKAGTDWRKASRQYSRERYNSLSLKLMKDGSLQS

>SlCDPK26

MGNCCVKPGKSAEKKNKKNNSKPNPFSIDYGGTKHASGSGNKLVVLKEPTGQNIHDKYDLGHELGRGEFGVTYLCTDLEGGEKYACKSISKKKLRTAVDIDDVRREVEIMKHLPVHPNIVTLKDTYEDDNAVHIVMELCEGGELFDRIVARGHYTERAAAGILKTVVEVVQMCHRQGVMHRDLKPENFLFGNKKETAPLKAIDFGLSVFFKPGERFNEIVGSPYYMAPEVLKRNYGPEVDVWSAGVILYILLCGVPPFWAETEQGVAQAIIRSVIDFKRDPWPKVSDNAKDLVKKMLDPDPTRRLTAHQVLEHPWLHNIKKAPNVSLGETVKARLKQFSVMNKLKKKALTVIAEFLSAEEVAGMKEAFEMMDTGKKGKINLNELKDGLQKLGHQIPDADLHILMEAADVDGDGSLNYPEFVAVSIHLRKMANDEHLHKAFSFFDKNQSGFIEIEELRSALRDEDDSNSEEVTNAIMHDVDTDKDGRISYEEFAAMMKAGTDWRKASRQYSRERFNSLSLKLMREGSLQVENKV

>SlCDPK27

MGNCCGTPGNSSENKKKKNKPNPFALDYGATQASGGDGNKLVVLKDPTGHNIQEKYDLGCELGRGEFGVTYLCTDVDTGDKYACKSISKKKLRTAVDIDDVRREVEIMKHLPKHPNIVTLKDTYEDDNAVHIVMELCEGGELFDRIVARGHYTERAAAVIMKTIVEVVQMCHMHGVMHRDLKPENFLFGNKKETAPLKAIDFGLSVFFKPGERFNEIVGSPYYMAPEVLKRNYGPEVDVWSAGVILYILLCGVPPFWAETEQGVAQAIIRSVVDFKRDPWPKVSDNAKDLVKKMLDPDPTRRLTAQQVLEHTWLQNIKKAPNVSLGETVKARLKQFSVMNKLKKRALTIMAEFLSAEEVAGMKDAFDMMDTGKKGKINLGELKNGLQKLGHQIPDVDLQILMEAADVDGDGSLNYGEFVAVSVHLRKMANDEHLHKAFSVFDRDQSGYIEIEELRSALSDEDGGNSEEVINAIMHDVDTDKDGRISYEEFAAMMKAGTDWRKASRQYSRERFNSLSLKLMRDGSIQVGKEEGR

>SlCDPK28

MGSCFSSSKVSGSNSNTPSTNNTTTNTNTAVNAHQNRRETSKAPSTTVVNPRNQEGCRDKGNINQKNQQKQPRNSQQNVKPSSRRQGGVIPCGKRTDFGYHKDFEKRYTIGKLLGHGQFGYTYVATDKSSGDRVAVKRIEKNKMVLPIAVEDVKREVKILKALGGHENVVQFYNSFEDDNYVYIVMELCEGGELLDRILSKKDSRYTEKDAAIVVRQMLKVAAECHLHGLVHRDMKPENFLFKSTKEDSPLKATDFGLSDFIRPGKKFQDIVGSAYYVAPEVLKRRSGPESDVWSIGVITYILLCGRRPFWDKTEDGIFKEVLRNKPDFRRKPWSNISNSAKDFVKKILVKDPRARLTAAQALSHPWVREGGDASEIPLDISVLSNMRQFVKYSRLKQFALRALASTLDEEEIADLRDQFSAIDVDKNGVISLEEMRQALAKDLPWKMKESRVLEILQAIDSNTDGLVDFPEFVAATLHVHQLEEHNSTKWQQRSQAAFEKFDVDKDGFITPEELKMHTGLRGSIDPLLEEADIDKDGKISISEFRRLLRTASMSSPTVRDSRGM

>SlCDPK29

MGNICFSSSKVSGSNSNTPSTTTTNTAAVNGHRNRRSSANPVSATTNTSRKQEGSHYNRQKGKDNGGVKQQTRNSQKNVKHNTRKQSGIIPCGKRTDFGYDKDFDNKFTIGKLLGHGQFGYTYVATDKSNGNRVAVKRIEKKKMVVPIAVEDVKREVKILKALAGHENVVDFYNAFEDDNYVYIVMELCEGGELLDRILAKKDSRYTEKDAAIVVGQMLKVAAQCHLHGLVHRDMKPENFLFKSSKEDSSLKATDFGLSDFIRPGKKFQDIVGSAYYVAPEVLKRKSGPESDVWSIGVITFILLCGRRPFWDKTEDGIFK

EVLRNKPDFRRKPWPTISNSAKDFVKKLLVKDPRARLTAAQALSHPWVREGGDASEIPLDISVLSNMRQFVKYSRLKQFALRALASTLDEEELADVRDQFSAIDVDKNGVISLEEMRQALAKDLPWKMKESRVLEILQAIDSNTDGLVDFPEFVAATLHVHQLEEHNLLKWQQRSQTAFEKFDVDRDGFITPEELRMHTGLKGSIDPLLEEADIDKDGKISLSEFRRLLRTASISSRMVNSPTVRGSRKI

>SlCRK1

MGQCCSKGVSGENGGSVVAIGDGNSAVSTNNRPKPPPSPVRQSVGNGMSYTNNSTPAHSFTASPFQSPYPAGIAPSPSPVGTPRRKFKWPFPPPSPAKPILSAIFKRQGGTSVKPKEGPIPEDEGGEGERQLDKSFGYPKNLTSKYELGKEVGRGHFGHTCWAKGKKGELKNQPVAVKIISKAKMTTAISIEDVRREVKILKALSGHQNLVKFYDAFEDANNVYIVMELCEGGELLDRILSRGGRYTEEDAKSIVVQILNVVAFCHLQGVVHRDLKPENFLFAKKDEDSPMKVIDFGLSDFIKPDQRLNDIVGSAYYVAPEVLHRSYSIEADMWSIGVITYILLCGSRPFWARTESGIFRSVLRADPNFEDSPWPAVSAEARDFVKRLLNKDHRKRMTASQALTHPWLRTENPFVPLDILIFKLVKSYIRTSPLKRAALKALSKALTEEELIYLKAQFNLLEPKAGFVSLDNFRMALMKQTTDAMREARVLDIINLLEPLSYKQMDFEEFCAAAISTYQLEALENWEHIASAAFNYFEQEGNRVISVEELAQEMNLGPTAYAFLKDWIRPSDRKLSFLGYTKFLHGVTMRSSSTRHHR

>SlCRK2

MGQCYGKTVPTVRDADGPITDVIGDSDHPLQQTPVSSNNLPSVKNTPARSSANSPWPSPYPHGVVGVTPSPARSTPRRFFKRPFPPPSPAKHIKASLAKRFGHAKPPAEGPIPEDDTPEPEQSLDKNFGYNKNFGAKFELGKEIGRGHFGHTCHAVGKKGELKDLPVAVKIISKTKMTTAVSIEDVRREVKILRALSGHKHLVKFHDGCEDANNVYIAMELCEGGELLDRILSRGGKYSEDDAKLIIVQILNVVAFCHLQGVVHRDLKPENFLFTSRDEDADMKLIDFGLSDFIRPDERLNDIVGSAYYVAPEVLHRSYSLEADIWSIGVITYILLCGSRPFWARTESGIFRSVLRSDPNFEDLPWPSVSPEAKDFVKRLLNKDYRKRMTAAQALTHPWLRSESHPIPLDIFVYKLVKSYLHATPLKRAALKALSKALTEDELVYLRAQFMLLEPSQDGRVSIENFRLALLGNATEAMRESRVHDILNAMTALSYKKLDFEEFCAAAISTYQLEALEEWEQIAAVAFQHFEQEGNRHVSVEELARELNVGPTAHSILRDWIRNDGKLNMLGYTKFLHGVTLRSTPVRRH

>SlCRK3

MGLCHGKPIQSPQNLSENPIIPIDNEQELNSHTGKTPKFPFYSPSPLPSAFKNSPANSSVTSTPLRFLKRVPPSPAKHIRALLARRHGSIKPNEATIPEGSECDIGLDKIFGYSKNFDSHYDLGEEVGRGHFGYTSAAKGKRGSLKGHDVAVKVIPKSKMTTAIAIEDVRREVKILRALTGHRNLVQFYDAYEDEENVYIVMELCKGGELLDRILARGGKYSEDDAKAVMVQLLSVVSYCHLQGVVHRDLKPENFLFVSKDENSPLKAIDFGLSDYVKPDERLNDIVGSAYYVAPEVLHRSYGTEADMWSIGVIAYILLCGSRPFWARTESGIFRAILKTDPNFDEAPWPSLSSDAVDFVKRLLNKDYRKRLTAAQALSHPWLAGHHDMKIPLDMIVYKLVKAYVYSSSLRKTALRALAKTLTIPQLAYLRDQFTLLGPSKSGLVALPNFKMAVMKNSTDAMKDSRVFDFVNTVSSLQYRKLDFEEFCAASISVHQLEGMESWEQHARRGYEFFEKDGNRPIMIEELASELGLSPSVPVHVVLQDWIRHSDGKLSFLGFVRLLHGVSSRTFQKA

>SlCRK4

MGACTSKPPRPNPYSPQEILPPPETPNVAETQKENEAKKSPFFPFYSPSPARFFLSKKSPARHSSASKSANSTPARLFKRPFPPPSPAKHIKALLLRRHGSVKPNAASIPEGEETEGANLDKSFGFSKQFVSKYEIGEEVGRGHFGYTCSAIVKKGELKGQKVAVKVIPKAKMTTAISIEDVRREVKILRALTGHNNLIQFYDAFEDRDNVYIVMELCQGGELLDRILARGGKYSEEDAKDVMVQILNVVAFCHLQGVVHRDLKPENFLFLSKDESSPLKAIDFGLSDFVRPDEKLNDIVGSAYYVAPEVLHRSYGIEADVWSIGVIAYILLCGSRPFWARTESGIFRAVLKADPTYDEAPWPTLTSEANDFVKRLLNKDPRKRMSAAQALCHPWMRNHSGAKLPLDILIFRLMKAYMRSSSLRKAALRALSKTLTADELFYLKEQFALLEPDKNGNIKLENIRSALMKYGTDAMKESRIPDFLASLNALQYRKMDFEEFCAAALSVHQLEALERWEQHARCAYEIFDKDGNRAIVIEELASELGLGPSIPVHAVLNDWIRHTDGKLSFLGFVKLLHGPSTRGLAKAQ

>SlCRK5

MGGCTSKPSPEPNYSNNDLYAPDGTAIPAKDNNSEDSKSKEGAEVGKKSPFFPFYSPSPAHYFFSKKSPARSPSNSTPRRFFKRPFPPPSPAKHIRAVLARRHGSVKPNAIPEGNELEGGGGLDKSFGFSKNFENKYELGEEVGRGHFGYTCKAKFKKGELKGQEVAVKVIPKAKMTTTIAIEDVRREVKILRALTGHENLVKFYDAYEDHENVYIVMELCEGGELLDRILLRGGKYTEDDARAVLTQILKVVAFCHLQGVVHRDLKPENFLFMSKEENAQLKAIDFGLSDFVKPDERLNDIVGSAYYVAPEVLHRSYSTEADVWSIGVISYILLCGSRPFWARTESGIFRAVLKADPGFEEQPWPTLSSEAKDFVKRLLNKDPRKRMTAAQALGHPWIKNSHDVEVPLDILIFKLMKTYMRSSALWKAALRALSKTLTVDELVHLKQQFALLEPNKNGTINLDNIKAALMKYATDAMKEARIHDFVASLNALQYRRMDFEEFCAAALSVYQLEALDQWEQHARCAYEIFEKDGNRAIVIEELASELGLGPSVPVHAVLLDWIRHTDGKLSFLGFAKLLHGVSSRSITKVQ

>SlCRK6

MGACTSRPSNYAGDSNGNRATLPVKSTPNNNEDGNSHQQERTKKDEADVGKKSPFFPFYSPSPAHYLFSKKSPLRSPANASTNSTPRRFFKRPFPPPSPAKHIRAVLARRHGSVKPNESAIPEVNETEVGGGGGGGAGLDKSFGFSKNFGNKYELGEEVGRGHFGYTCKAKFRKGEVKGQEVAVKVIPKSKMTTAIAIEDVRREVKILRALTGHSNLVKFYDSYEDHTNVYIVMELCEGGELLDRILSRGGKYSEDDAKTVMIQILKVVAFCHLQGVVHRDLKPENFLFTSKEENAQLKAIDFGLSDFVKPDERLNDIVGSAYYVAPEVLHRSYSTEADVWSIGVIAYILLCGSRPFWARTESGIFRSVLKADPSFEEQPWPTLSSEAKDFVKRLLNKDPRKRMTAAQALGHPWIKNSHNIEVPLDILIFKLMKAYMRSSALRKAALRALSKTLTVDELFYLKEQFALLQPSKNGTISFDHVKTVLMKHATDAMKEARIHDFLASLNALQYRRMDFEEFCAAALSVHQLEAFDRWEQHARCAYEIFEKEGNRAIMIEELASELGLSPSVPVHAVLHDWLRHTDGKLSFLGFAKLLHGVSSRSIAKVQ

>OsCPK1

MGNRTSRHHRAAPEQPPPQPKPKPQPQQQQQQWPRPQQPTPPPAAAPDAAMGRVLGRPMEDVRATYTFGRELGRGQFGVTYLVTHKATGKRFACKSIATRKLAHRDDIEDVRREVQIMHHLTGHRNIVELRGAYEDRHSVNLIMELCEGGELFDRIIARGHYSERAAAALCREIVAVVHSCHSMGVFHRDLKPENFLFLSKSEDSPLKATDFGLSVFFKPGEHFKDLVGSAYYVAPEVLKRNYGAEADIWSAGVILYILLSGVPPFWAESEDGIFDAVLRGHIDFSSEPWPSISNGAKDLVKKMLRQDPKERLTSAEILNHPWIREDGEAPDKPLDITVISRMKQFRAMNKLKKVALKVVAENLSDEEITGLKEMFRSLDTDNSGTITLEELRSGLPKLGTKISESEIRQLMEAADVDGNGTIDYAEFISATMHMNRLEKEDHILKAFEYFDKDHSGYITVDELEEALKKYDMGDDKTIKEIIAEVDTDHDGRINYQEFVAMMRNNNPEIAPNRRRMF

>OsCPK2

MGNCCPGSGDAEPASSDASTGNGSSSFKAGASPSSAPAQNKPPAPIGPVLGRPMEDVRSIYTIGKELGRGQFGVTSLCTHKATGQKFACKTIAKRKLSTKEDVEDVRREVQIMYHLAGQPNVVELKGAYEDKQSVHLVMELCAGGELFDRIIAKGHYTERAAASLLRTIVEIIHTCHSLGVIHRDLKPENFLLLSKDEDAPLKATDFGLSVFFKQGEVFKDIVGSAYYIAPEVLKRSYGPEADIWSVGVILYILLCGVPPFWAESEHGIFNSILRGQVDFTSDPWPRISASAKDLVRKMLNSDPKKRISAYEVLNHPWIKEDGEAPDTPLDNAVMNRLKQFRAMNQFKKAALRVIAGCLSEEEIRGLKEMFKSMDSDNSGTITVDELRKGLSKQGTKLTEAEVQQLMEAADADGNGTIDYDEFITATMHMNRMDREEHLYTAFQYFDKDNSGCISKEELEQALREKGLLDGRDIKDIISEVDADNDGRIDYSEFAAMMRKGNPEANPKKRRDVVI

>OsCPK3

MGNCCRSPAAAAREDVKSSHFPASAGKKKPHQARNGGVGGGGGGGGGGGGGGGAGQKRLPVLGEEGCELIGGIDDKYALDRELGRGEFGVTYLCMDRDTKELLACKSISKRKLRTAVDVEDVRREVAIMRHLPKSASIVSLREACEDEGAVHLVMELCEGGELFDRIVARGHYTERAAANVTRTIVEVVQLCHRHGVIHRDLKPENFLFANKKENSPLKAIDFGLSIFFKPGEKFSEIVGSPYYMAPEVLKRNYGPEIDIWSAGVILYILLCGVPPFWAETEQGVAQAILRGNIDFKREPWPNVSENAKDLVRRMLEPDPKLRLTAKQVLEHPWLQNAKKAPNVPLGDIVKSRLKQFSRMNRFKRRALRVIADHLSAEEVEDIKEMFKAMDTDNDGIVSYEELKSGIAKFGSHLAESEVQMLIEAVDTNGKDALDYGEFLAVSLHLQRMANDEHLRRAFLFFDKDGNGYIEPEELREALVDDGAGDSMEVVNDILQEVDTDKDGKISYDEFVAMMKTGTDWRKASRHYSRGRFNSLSMKLIKDGSVKLVNE

>OsCPK4

MGACFSSHTATAAADGGSGKRQQRKGDHKGKLPDGGGGEKEKEAARVEFGYERDFEGRYQVGRLLGHGQFGYTFAATDRASGDRVAVKRIDKAKMVRPVAVEDVKREVKILKELKGHENIVHFYNAFEDDSYVYIVMELCEGGELLDRILAKKNSRYSEKDAAVVVRQMLKVAAECHLHGLVHRDMKPENFLFKSTKEDSPLKATDFGLSDFIKPGKKFHDIVGSAYYVAPEVLKRRSGPESDVWSIGVITYILLCGRRPFWNKTEDGIFREVLRNKPDFRKKPWPGISSGAKDFVKKLLVKNPRARLTAAQALSHPWVREGGEASEIPVDISVLSNMRQFVKYSRFKQFALRALASTLKEEELADLKDQFDAIDVDKSGSISIEEMRHALAKDLPWRLKGPRVLEIIQAIDSNTDGLVDFEEFVAATLHIHQMAELDSERWGLRCQAAFSKFDLDGDGYITPDELRMVQHTGLKGSIEPLLEEADIDKDGRISLSEFRKLLRTASMSNLPSPRGPPNPQPL

>OsCPK5

MGNTCGVTLRSKYFASFRGASQRHDEAGYAPVATSAAAAAAADEPAGKKAPRGSAAAADAPHAASMKRGAPAPAELTANVLGHPTPSLSEHYALGRKLGQGQFGTTYLCTDLATGVDYACKSIAKRKLITKEDVEDVRREIQIMHHLAGHRNVVAIKGAYEDPQYVHIVMELCAGGELFDRIIERGQFSERKAAELTRIIVGVIEACHSLGVIHRDLKPENFLLANKDDDLSLKAIDFGLSVFFKPGQVFTDVVGSPYYVAPEVLRKCYGPEADVWTAGVILYILLSGVPPFWAETQQGIFDAVLKGVIDFDSDPWPVISDSAKDLIRRMLNPRPKERLTAHEVLCHPWICDHGVAPDRPLDPAVLSRIKQFSAMNKLKKMALRVIAESLSEEEIAGLKEMFKAMDTDNSGAITYDELKEGMRKYGSTLKDTEIRDLMEAADVDNSGTIDYIEFIAATLHLNKLEREEHLVAAFSYFDKDGSGYITVDELQQACKEHNMPDAFLDDVIKEADQDNDGRIDYGEFVAMMTKGNMGVGRRTMRNSLNISMR

>OsCPK6

MGNYYSCGASSTSSPTSPSLVDYYYCYHRYPSSCSSTSTATSSGGRMPIRSHQQRLSSPTAVLGHETPALREVYTVGRKLGQGQFGTTYLCTQVSTGAEYACKSIAKRKLLSPEDVEDVRREIQIMHHLAGHGSVVTIQGAYEDNLYVHIVMELCEGGELFDRIVERGYFSERKAAEITRVIVGVVEACHSLGVMHRDLKPENFLLKESSSSSSLKAIDFGLSVFFKPGQVFSDVVGSPYYVAPEVLCKHYGPEADVWTAGVIVYILLSGVPPFWAETQQGIFDAVLRGSLDFDSDPWPTISDSAKDLIRRMLRSPPRERLTAHQVLCHPWVCDDGVAPDRPLAPAVLSRLKQFSAMNRLKKMALRVIARNLSEEELAGLKEMFKAMDTDASGAITFDELKEGLRRYGSNLREAEIRDLMDAADVDKSGTIDYDEFIAATVHLNKLEREEHLLAAFAYFDRDGSGYITVDELEHACRDHNMADVGIDDIIREVDQDNDGRIDYGEFVAMMKKGAIDIIGNGRLTIGRPTTATSDDPSPTISSSSR

>OsCPK7

MGNQCQNGTLGSDYHNRFPREHAVGYVQGDSYLDLKKFDDTWPEVNNFKPTAASILRRGLDPTSINVLGRKTADLREHYIIGRKLGQGQFGTTYLCTEINTGCEYACKTIPKRKLITKEDVEDVRREIQIMHHLSGHKNVVAIKDVYEDGQAVHIVMELCAGGELFDRIQEKGHYSERKAAELIRIIVSIVAMCHSLGVMHRDLKPENFLLLDKDDDLSIKAIDFGLSVFFKPGQVFTELVGSPYYVAPEVLHKRYGPESDVWSAGVILYVLLSGVPPFWAETQQGIFDAVLKGHIDFQSDPWPKISDSAKDLIRKMLSHCPSERLKAHEVLRHPWICENGVATDQALDPSVISRLKQFSAMNKLKKLALRVIAERLSEEEIAGLREMFKAVDTKNRGVITFGELREGLRRFGAEFKDTEIGDIMEAAHNDNNVTIHYEEFIAATLPLNKIEREEHLLAAFTYFDKDGSGYITVDKLQRACGEHNMEDSLLEEIISEVDQNNDGQIDYAEFVAMMQGSNVGLGWQTMESSLNVHSLCWLFFQRPVLRNESRHVCVTLISMLAFL

>OsCPK8

MGNCCGTPATAEEGGKRRRRGKQKKANPFTVAYNRAPSSAGAAAGRPGLMVLRDPTGRDLGARYELGGELGRGEFGITYLCTEAETGDRYACKSISKRKLRTPVDVEDVRREVEIMRHMPSHPNIVSLRAAYEDEDNVHLVMELCEGGELFDRIVARGHYTERAAAAVTRTIVEVVQMCHRHGVMHRDLKPENFLYANKKDSSPLKAIDFGLSVFFRPGERFTEIVGSPYYMAPEVLKRHYGPEVDVWSAGVILYILLCGVPPFWAETEQGVAQAIIRSVVDFKREPWPRVSEPAKDLVKRMLDPNPMTRLTAEQVLEHPWLHDSKKMPDIPLGDAVRARLQQFAAMNKLKKKALKVIAEHLSAEEAADIKDMFDKMDVSKNGQLTFEDFKAGIRKLGNQMPDSDLKILMDAADIDKNGILDYQEFVAVSIHVRKIGNDEHIQKAFSYFDQNKSGYIEIEELREALVDEIDGNDEDIINSIIRDVDTDKDGKISYDEFAVMMKAGTDWRKASRQYSRQRFSNLSLKLQKDGSISDDTQ

>OsCPK9

MGNTCCVAPATTDEVGAPPRDHHHAAKKSPAPSATTTTATRQRHGQEPKPKPKPRARAKPNPYDWAPPRVLPARGGAAASAVRVLEGVVPHHPRLRVTDKYQLGRELGRGEFGVTHLATDRATRERLACKSIPKRRLRTAVDVADVRREVAIMASLPDHPALVRLRAAYEDADAVHLVMELCDGGELFDRIVARGRYTERAAAAAARTVAEVVRACHAHGVMHRDLKPENFLYAGKAEDAQLKAIDFGLSVFFRPGERFREIVGSPYYMAPEVLRRDYGPEVDIWSAGVILYILLCGVPPFWAETEQGVARAILRGAADFDREPWPRISRAAKSLVRQMLDVDPRRRPTAQQVLDHPWLHHAARAPNVPLGDVVRARLKQFSLMNRLKKKAMRVIAEHLSVEEVEVIKDMFALMDTDNNGRVTLQELKDGLTKVGSKLAEPEMELLMEAADVDGNGYLDYGEFVAVTIHLQRLSNDNHLRTAFLFFDKDGSGYIDRAELADALADDSGHADDAVLDHILREVDTDKDGRISYEEFVAMMKSGTDWRKASRQYSRERFKTLSNSLIKDGSITMAR

>OsCPK10

MGNTCVGPSISKNGFFQSVSTVLWKARQDGDDALPGANGAPDGGGQGRLPAPPPPTSDAPLAVQNKPPEHVKIVSTTDTASAEQDASKSSAGSDSGEAARPRPRVPPVKRVSSAGLLVGSVLKRKTESLKDKYSLGRKLGQGQFGTTYLCVERATGKEFACKSILKRKLVTDDDVEDVRREIQIMYHLAGHPNVISIRGAYEDAVAVHLVMELCAGGELFDRIVQKGHYTERKAAELARVIVGVVEVCHSMGVMHRDLKPENFLFADQTEEAALKTIDFGLSIFFRPGQVFTDVVGSPYYVAPEVLKKKYGQEADVWSAGVIIYILLCGVPPFWAENEQGIFEEVLHGRLDFQSEPWPSISEGAKDLVRRMLVRDPKKRLTAHEVLRHPWVQVGGLAPDKPLDSAVLSRMKQFSAMNKLKKMALRVIAENLSEDEIAGLKEMFKMIDTDNSGQITFEELKVGLKKVGANLQESEIYALMQAADVDNSGTIDYGEFIAATLHMNKIEREDHLFAAFQYFDKDGSGYITADELQLACEEFGLGDVQLEEMIREVDEDNDGRIDYNEFVAMMQKPTMGLPAKKSGGLQNSFSIGFREALRMS

>OsCPK11

MGNNCVGPSAAGQNGFFANVALWRPRPADAAPPALPPPSSAPSDQAPEPVTIPPSEHSSHHSSRSTDPSTPTSAAEQPANKAAPKVKRVQSAGLLADSVLKRDVNTARLKDLYTIGKKLGQGQFGTTYLCVEKATGREFACKSIAKRKLLTQEDVEDVRREIQIMHHLAGHANVVSIVGAYEDAVAVQLVMELCAGGELFDRIIQRGHYSEKAAAQLARVIVGVIEACHSLGVMHRDLKPENFLFIHQKEDSPLKAIDFGLSIFFKPGETFTDVVGSPYYVAPEVLMKHYGREVDVWSAGVIIYILLSGVPPFWDESEQGIFEQVLKGDLDFSSEPWPNISESAKDLVRKMLIRDPKKRLTAHEALCHPWVCVDGVAPDKPLDSAVLSRLKQFSAMNKLKKMALRVIAESLSEEEIAGLKEMFKMLDTDNSGHITLEELKTGLQRVGANLMDSEIDALMEAADIDNSGTIDYGEFIAATLHINKVEKEDKLFAAFSYFDKDGSGYITQDELQKACEEFGIGDTRIEDIIGDIDQDNDGRIDYNEFVEMMQKGNNAMGKMGQHSTGNFGLGEALKLR

>OsCPK12

MGNCFTKTYEIPITSGTMRRPASTAERSKARGGDEPGTWRRPSFPRHGAPPHRPPTGSSSAAGALSRRASGGGGEMGPVLQRAMVSVRSLYQLDRKLGSGQFGTTYLCTERATGNRYACKSVSKRKLVRRTDVDDVRREITILQHLSGQPNIAEFRGAYEDNDHVHLVMEFCSGGELFDRITAKGSYSERQAAAVCRDILTVVHVCHFMGVIHRDLKPENFLLASADDDAPLKAIDFGLSVFIEEGKVYKDIVGSAYYVAPEVLQRNYGKEADIWSAGVILYILLCGTPPFWAETEKGIFDAILVNQVDFSTSPWPSISESAKDLIRQMLHRDPQKRITASQALEHRWLKEGGASDRPIDSAVLSRMKQFKAMNKLKQLALKVIAENLSPEEIKGLKQMFNNMDTDRSGTITVEELKVGLTKLGSRISEAEVQKLMEAVDVDKSGSIDYSEFLTAMINKHKLEKEEDLLRAFQHFDKDNSGYITRDELEQAMAEYGMGDEANIKQVLDEVDKDKDGRIDYEEFVEMMRKGIQT

>OsCPK13

MGNACGGSLRSKYLSFKQTASQRHDTDDNNNAAAADSPKKPSRPPAAAKTDDHPVSASAPAAAMRRGQAPADLGSVLGHPTPNLRDLYAMGRKLGQGQFGTTYLCTELSTGVDYACKSISKRKLITKEDIEDVRREIQIMHHLSGHKNVVAIKGAYEDQLYVHIVMELCAGGELFDRIIQRGHYSERKAAELTRIIVGVVEACHSLGVMHRDLKPENFLLANKDDDLSLKAIDFGLSVFFKPGQTFTDVVGSPYYVAPEVLLKHYGPEADVWTAGVILYILLSGVPPFWAETQQGIFDAVLKGFIDFDSDPWPVISESAKDLITKMLNPRPKERLTAHEVLCHPWIRDHGVAPDRPLDPAVLSRIKQFSAMNKLKKMALRVIAESLSEEEIAGLKEMFQTMDADNSGAITYDELKEGLRKYGSTLKDTEIRDLMDAADIDNSGTIDYIEFIAATLHLNKLEREEHLVAAFSYFDKDGSGYITVDELQQACKEHNMPDAFLDDVINEADQDNDGRIDYGEFVAMMTKGNMGVGRRTMRNSLNISMRDAPGAL

>OsCPK14

MGNCCPPGSSSEPDPPPASSGSSRPAGSAGAAASPATISPSAAPAPAKPPAPIGPVLGRPMEDVKSIYTVGKELGRGQFGVTSLCTHKATGQRFACKTISKRKLSTKEDVEDVRREVQIMYHLAGQPGVVELKGAYEDKHAVHLVMELCAGGELFDRIIAKGHYTEHAASSLLRTIVEIIHTCHSMGVIHRDLKPENFLLLSKDEHAPLKATDFGLSVFFKEGEVFRDIVGSAYYIAPEVLKRSYGPEADIWSIGVMLYILLCGVPPFWAESEHGIFNSILRGHVDFSSEPWSRISHGAKDLVRRMLHSDPKQRISAYDVLNHPWIKEDGEAPDTPLDNAVLGRLKQFRAMNQFKKAALRVIAGCLSEEEIRGLKEMFKSMDSDNSGTITVDELRKGLAKKGTKLTEAEVQQLMEAADADGNGTIDYEEFITATMHMNRMDREEHLYTAFQYFDKDNSGYITIEELEQALREKGLMDGREIKDIISEVDADNDGRINYTEFVAMMRKGDPEANPKKRRDVVL

>OsCPK15

MVSSSSSPQSKPPKPKPKPLSPPMGARASRHRQSPDQSQSQSPSPHHKHHHHHQTTRAPKPKPKPQPPPPQQPRSQPPPPPRHQPQQAPQQAAAEDGVGRVLGRPMEDVRATYTFGRELGRGQFGVTYLATHKPTGRRYACKSIAARKLARPDDLDDVRREVHIMHHLTGHRNIVELRGAYEDRHSVNLVMELCEGGELFDRIIARGHYSERAAAALCREIVSVVHSCHSMGVMHRDLKPENFLFLNKREDSPLKATDFGLSVFFKPGEQFRDLVGSAYYVAPEVLKRLYGAEADIWSAGVILYILLSGVPPFWAENEDGIFDAVLQGHIDFSSEPWPSISSGAKDLVKRMLRQDPKERLTAAEILNHPWIREDGEAPDKPLDITVISRMKQFRAMNKLKKVALKVVAENLSEEEIVGLKEMFKSLDTDNSGTITLEELRAGLPKLGTKISESELRQLMEAADVDGNGSIDYVEFISATMHMNRLEKEDHIYKAFEYFDKDHSGFITVDELEEALTKYDMGDEATIKEIIAEVDTDHDGRINYQEFVAMMKNNSPEIVPNRRRMF

>OsCPK16

MGNCCRSPAAAAREDVKTSHFPASTGGGKKKPHQARNGGGGGGGGGGGGWEKKRLSVLGEEGSEVNGGIEEKYALDRELGRGEFGVTYLCMDRCSRELLACKSISKRKLRTPVDVEDVRREVAIMRHLPRSASIVSLREACEDDGAVHLVMELCEGGELFDRIVARGHYTERAAAAVTRTIVEVVQLCHRHGVIHRDLKPENFLFANKKENSPLKAIDFGLSIFFKPGEKFSEIVGSPYYMAPEVLKRNYGPEIDIWSAGVILYILLCGVPPFWAETEQGVAQAILRGNIDFKREPWPNVSDNAKDLVRQMLQPDPKLRLTAKQVLEHTWLQNAKKAPNVPLGDIVKSRLKQFSRMNRFKRRALRVIADHLSAEEVEDIKDMFKVMDTDNDGIVSYEELKSGIAKFGSHLAESEVQMLIEAVDTNGRGALDYGEFLAVSLHLQRMANGEHLRRAFLFFDKDGNGYIEPEELQEALVEDGATDIMEVVKDILQEVDTDKDGKISYEEFVAMMKTGTDWRKASRHYSRGRFNSLSIRLIKDG

SVKLGNE

>OsCPK17

MGNTCVGPSSAADRHGFFHSVSLAVLWRPGGRAEPSQPPGYPPRESSHSSVTSSTAPERVTIADSDLSSSTPNKGGNKPKVRRVQSAGLLADSVLKRDSERLKDLYTLGKKLGQGQFGTTYQCVEKATGKVLACKSIAKRKLVSEEDVEDVRREIQIMHHLAGHPSVVSIVGAYEDAVAVHLVMELCAGGELFDRIVQRGHYSEKAAAQLARVIIGVVEACHSLGVMHRDLKPENFLFVNHKEDSPLKTIDFGLSIFFKPGENYSDVVGSPYYVAPEVLMKHYGREVDVWSAGVIIYILLSGVPPFWDESEQGIFEKVLKGDLDFSSDPWPAISDSAKDLVRKMLNRDPRKRLTAHEALCHPWVCVDGVAPDKPLDSAVLTRLKQFSAMNKLKKMALRVIAENLSEDEIAGLREMFKMLDTDNSGQITLEELKTGLRRVGANLKDSEITTLMEAADIDNSGSIDYGEFIAATMHLNKVEREDNLFAAFSYFDKDSSGYITQDELQKACEEFGIGDAHLEDIIKDIDQDNDGRIDYNEFVTMMQKGNNPLGKKGQGQLSFGLREALKLG

>OsCPK18

MGLCSSSSARRDAGTPGGGNGAGNKDNAGRKGIVACGKRTDFGYDKDFEARYALGKLLGHGQFGYTFAAVDRRSSERVAVKRIDKNKMVLPVAVEDVKREVKILKALQGHENVVHFYNAFEDDNYVYIVMELCEGGELLDRILAKKDSRYSEKDAAVVVRQMLKVAAECHLHGLVHRDMKPENFLFKSTKEDSSLKATDFGLSDFIRPGKHFRDIVGSAYYVAPEVLKRKSGPESDVWSIGVITYILLCGRRPFWDKTEDGIFKEVLKNKPDFRRKPWPNITPCAKDFVQKLLVKDPRARLTAAQALSHEWVREGGQASDIPLDISVLHNMRQFVKYSRFKQFALRALASTLNAEELSDLRDQFNAIDVDKNGTISLEELKQALAKDVPWRLKGPRVLEIVEAIDSNTDGLVDFEEFVAATLHVHQLVEHDTEKWKSLSQAAFDKFDVDGDGYITSDELRMQTGLKGSIDPLLEEADIDRDGKISLDEFRRLLKTASMSSRNVQTPRSVHRS

>OsCPK19

MGSCCSRATSPDSGRGGANGYGYSHQTKPAQTTPSYNHPQPPPPAEVRYTPSAMNPPVVPPVVAPPKPTPDTILGKPYDDVRSVYSLGKELGRGQFGVTYLCTEIASGKQYACKSISKRKLVSKADKEDIRREIQIMQHLSGQQNIVEFRGAYEDKSNVHVVMELCAGGELFDRIIAKGHYSERAAATICRAVVNVVNICHFMGVMHRDLKPENFLLATKEENAMLKATDFGLSVFIEEGKMYRDIVGSAYYVAPEVLRRNYGKEIDVWSAGVILYILLSGVPPFWAETEKGIFDAILQGEIDFESQPWPSISESAKDLVRKMLTQDPKKRITSAQVLQHPWLRDGEASDKPIDSAVLSRMKQFRAMNKLKKMALKVIASNLNEEEIKGLKQMFTNMDTDNSGTITYEELKAGLAKLGSKLSEAEVKQLMEAADVDGNGSIDYVEFITATMHRHKLERDEHLFKAFQYFDKDNSGFITRDELESALIEHEMGDTSTIKDIISEVDTDNDGRINYEEFCAMMRGGGMQQPMRLK

>OsCPK20

MGNCCVTPEGSGRGRKKQQQEQKQKQKEPKQQQQQQKKGKKPNPFSIEYNRSSAPSGHRLVVLREPTGRDIAARYELGGELGRGEFGVTYLCTERETGDAYACKSISKKKLRTAVDIEDVRREVDIMRHLPKHPNIVTLRDTYEDDNAVHLVMELCEGGELFDRIVARGHYTERAAALVTRTIVEVVQMCHKHGVMHRDLKPENFLFANKKETAALKAIDFGLSVFFTPGERFTEIVGSPYYMAPEVLKRNYGPEVDVWSAGVILYILLCGVPPFWAETEQGVAQAIIRSVIDFKRDPWPRVSDNAKDLVKGMLNPDPRRRLNAQQVLDHPWLQNIKKAPNVNLGETVKARLQQFSVMNKFKKHALRVIAEHLSVEEVAGIKDMFEKMDLNKDNMINFDELKLGLHKLGHQMADADVQILMDAADVDGNGSLDYGEFVALSVHLRKIGNDEHLHKAFAYFDRNQSGYIEIDELRESLADDLGANHEEVINAIIRDVDTDKDGKISYDEFAAMMKAGTDWRKASRQYSRERFTSLSLKLQKDGSLQLTTTQ

>OsCPK21

MGGCYSAYASSRKLRGRISKISLVIPDPVPDAEAASPRKDGVDGDGDDVRGGGGGCDDGGDVVAIATTTADEFARRYVLGKELGRGEFGVTRRCSDAATGEALACKTIRKHRRLAPPRVTAAKAAAAHGEDVKREVAIMRRMSSASSSRGGGAASSAAVVRLREACEDAADGSVHLVMELCEGGELFDRIVARGHYSERAAANIFRTIVDVVQLCHSNGVIHRDLKPENFLFANKSEDSPLKVIDFGLSVFFKPGDRFTEVVGSAYYMAPEVLRRSYGPEVDVWSAGVILYILLCGVPPFWGDNDEKIAQAILRGAIDFNREPLPRVSANAKDLVRRMLDPNPSTRLTAKQVLEHPWLKNADTAPNVSLGDAVRARLQQFSAMNKFKKKALGVVARNLPGEEVDKYVQMFHHMDKDKNGHLSLDELLEGLHINGQPVPEPEIRMLLEAADTDGNGTLDCDEFVTVSVHLKKMSNDEYLAAAFNYFDKDGSGFIELDELREEVGPNEQAILEILRDVDTDKDGRISYQEFELMMKSGADWRNASRHFSRANFSTLSRRLCKDTLTP

>OsCPK22

MGGCSSAFAVSTRMIRFSRGRVPAAILPVTSNDEPCCSCSPENNNKNNDGGGGGCDGGEHQKGKSWRRWQYRRCGGGGGGGGGRKNAILGDAADVKTAAGFAERYRLGAELGRGEFGVTRRCSDAATGEALACKTIRRKRLRRCRGDAEDVRREVEILRRISALGAGADSVVRLRDACEDSDGVHLVMELCEGGELFDRIFARGHYTERAAAKLARTIVGVVQLCHENGVMHRDLKPENFLFANKSEDSPLKAIDFGLSVFFKPGERFTQVVGSTYYMAPEVLNRSYGPEADVWSAGVILYILLCGVPPFWGDNDEKTVTAILQGGINFQREPWPKVSPHAKDLVSKMLDPDPSTRLTAKEVLEHPWLKNADRAPNVSLGEIVRSRLMQFSAMNKFKKKALGVVAKNLPVEEMDKYTQMFHKMDKDNSGNLTLEDLKLGLQINGHPVPETEIEMLLEAGDIDGNGTLDCEEFVTVLLHIKKMSNEEYLPKAFKFFDKDGNGFIEMEELMDALGDELGPTEQVVKDIIRDIDTDKDGRISYQEFESMMISGSDWRNASRRYSKANFSSLSRKLCKGNS

>OsCPK23

MGNSCQNGTYGNNYQNSNRFQNDRFASRYVDGNDTEDCYSGSSRASLAGALRQGLNLKSPVLGYKTPNVRELYTLGRELGQGQFGKTYLCTEISTGCQYACKTILKSNLRCVSDIEDVRREIQIMHHLSGQKNIVTIKDTYEDEQAVHIVMELCAGGELFSKIQKRGHYSERKAAELIKIIVGIIETCHSHGVMHRDLKPENFLLLDADDEFSVKAIDFGLSVFFRPGQVFREVVGSPYYIAPEVLEKRYGPEADIWTAGVILYVLLTGVPPFWADTQSGIYEKVLDGRIDFKSNRWPRISDSAKDLIKKMLCPYPSERLKAHEVLKHPWICDNGVATNRALDPSVLPRLKQFSAMNRLKKLSLQIIAERLSEEEIVGLREMFKAMDTKNRSVVTFGELKGLKRYSSVFKDTEINDLMEAADDTTSTINWEEFIAAAVSLNKIEREKHLMAAFTYFDKDGSGFITVDKLQKACMERNMEDTFLEEMILEVDQNNDGQIDYAEFVTMMQSNNFGLGWQTVESSLNVALREAPQVY

>OsCPK24

MQPDPSGSGGDGNANAKAKLAPPPVTAAGGRPVSVLPHKTANVRDHYRIGKKLGQGQFGTTYLCVDKASGGEFACKSIPKRKLLCREDYEDVWREIQIMHHLSEHPNVVRIRGAYEDALFVHIVMELCAGGELFDRIVAKGHYTERAAAQLIRTIVAVVEGCHSLGVMHRDLKPENFLFASAAEDAPLKATDFGLSMFYKPGDKFSDVVGSPYYVAPEVLQKCYGPESDVWSAGVILYILLCGVPPFWAETEAGIFRQILRGKLDFESEPWPSISDSAKDLVRNMLCRDPTKRLTAHEVLCHPWIVDDAVAPDKPIDSAVLSRLKHFSAMNKLKKMALRVIAESLSEEEIGGLKELFKMIDTDDSGTITFDELKEGLKRVGSELTEHEIQALMEAADIDNSGTIDYGEFIAATLHMNKLEREENLVSAFSFFDKDGSGFITIDELSQACREFGLDDLHLEDMIKDVDQNNDGQIDYSEFTAMMRKGNAGGAGRRTMRNSLQLNLGEILNPSNS

>OsCPK25

MGQCCTGGGKAVAGDEAEPGTSKAAPPSRGTSSKNGSAKQQPCSPAAKAAATEAAAAASSSKKPAGPIGEVLERPMEEVRTTYSIGKELGRGQFGVTHLCTHKATGEKLACKTIAKRKLANKEDVDDVRREVQIMHHLSGQPNIVDLRGAYEDKHNVHLVMELCAGGELFDRIIARGHYTERAAAALLRAIVGIVHTCHSMGVIHRDLKPENFLLLSKGDDAPLKATDFGLSVFFKEGEVFRDIVGSAYYIAPEVLKRKYGPEADIWSIGVMLYIFLAGVPPFWAESENAIFTAILRGQIDLASEPWPKISSGAKDLVRKMLNINPKERLTAFQVLNHPWIKEDGDAPDVPLDNVVLNRLKQFRAMNQFKKAALRIIAGCLSEEEIKGLKEMFKNIDKDNSGTITLEELKNGLAKQGTKFSDNEIEQLMEAADADGNGIIDYEEFVTATVHMNKMDREEHLYTAFQYFDKDNSGYITKEELEQALKEQGLYDANEIKDVITDADSNNDGRIDYSEFVAMMRKGSGCAEATNPKKKRRDLVL

>OsCPK26

MGQCCTGGGKAVAGDEAEPGTSKAAPPSRGTSSKNGSAKQQPCSPAAKAAATEAAAAASSSKKPAGPIGEVLERPMEEVRTTYSIGKELGRGQFGVTHLCTHKATGEKLACKTIAKRKLANKEDVDDVRREVQIMHHLSGQPNIVDLRGAYEDKHNVHLVMELCAGGELFDRIIARGHYTERAAAALLRAIVGIVHTCHSMGVIHRDLKPENFLLLSKGDDAPLKATDFGLSVFFKEGEVFRDIVGSAYYIAPEVLKRKYGPEADIWSIGVMLYIFLAGVPPFWAESENAIFAAILRGQIDLASEPWPKISSGAKDLVRKMLNINPKERLTAFQVLNHPWIKEDGDAPDVPLDNVVLNRLKQFRAMNQFKKAALRIIAGCLSEEEIKGLKEMFKNIDKDNSGTITLEELKNGLAKQGTKFSDNEIEQLMEAADADGNGIIDYEEFVTATVHMNKMDREEHLYTAFQYFDKDNSGYITKEELEQALKEQGLYDANEIKDVITDADSNNDGRIDYSEFVAMMRKGSGCAEATNPKKKRRDLVL

>OsCPK27

MGNVCIGPRRNFAKNGLLGILRPRHAAPSSPSQPTTTSRSIPVVLPSAPSSKPPPPTQTAPPVPVVISEPPPPQPQPEPQPAAPSQPPPPQEQPSPPPPASSNTTQQPPPPQQRQQSRAKKPAHIKRISSAGLQVESVLRRKTENLKDKYSLGRKLGQGQFGTTYLCVDKANGGEYACKSIAKRKLLTDEDVEDVRREIQIMHHLAGHPNIISIRGAYEDAVAVHVVMELCAGGELFDRIVRKGHYTERQAAGLARVIVAVVESCHSLGVMHRDLKPENFLFVGNEEDAPLKTIDFGLSMFFRPGEVFTDVVGSPYYVAPEVLKKSYGQEADVWSAGVIIYILLCGVPPFWAETEQGIFEQVLHGTLDFESDPWPNVSDGAKDLLRKVLVRDPKKRLTAHEVLCHPWLQMSGSAPDKPLDSAVLSRLRQFSAMNKLKKMALRVIAENLSEEEIAGLKEMFKMMDTDNSGQINYEELKAGLERVGANMKESEIYQLMQAADIDNSGTIDYGEFIAATLHLNKVEREDHLYAAFQYFDKDGSGYITSDELQQACDEFGIEDVRLEDMIGEVDQDNDGRIDYNEFVAMMQKTTTGFGKKGGHNFSGFRDALKSHS

>OsCPK28

MQPDPQPHGRGREKAAGAGPRLPPPVTAPSVGRPASVLPHKTANVRDHYRIGKKLGQGQFGTTYLCVGKPDGGEYACKSIPKRKLLCREDYEDVWREIQIMHHLSEHPNVVRIRGAYEDALFVHIVMELCAGGELFDRIVAKGHYTERAAALLIRTIVGVVEGCHSLGVMHRDLKPENFLFASTAEDAPLKATDFGLSVFYKPGDKFSDVVGSPYYVAPEVLQKIYGPEADVWSAGVILYILLCGVPPFWAETESGIFRQILRGKLDLESDPWPSISDSAKDLVRNMLIRDPTKRFTAHEVLCHPWIVDDAVAPDKPIDSAVLSRLKHFSAMNKLKKMALRVIAESLSEEEIGGLKELFKMIDTDNSGTITYDELKNGLKRVGSDLMEPEIQALMDAADIDNSGTIDYGEFLAATLHMNKLEREENLVSAFTFFDKDGSGFITIDELSQACEQFGLSDVHLEDMIKDVDQNNDGQIDYSEFAAMMRKGNAGGANAGGVTSTGGTGRRTMRNSLRVNLGDILKPNEN

>OsCPK29

MGNCCVSRPSGADKRRRCGSSTAPHTRGGRRVIGAANMRCLSTVSSVSDAARAVMSNEPATVLGNSGSSGNGGVMAAEEMLRRYEIGEELGRGEFGVTRRCRDAVTGERLACKSISKRKLRSSVDVEDVRREVAIMRSLPAHANVVRLREAFEDADAVHLVMEVCEGGELFDRIVARGHYTERAAAAVMRTIMDVVQHCHKNGVMHRDLKPENFLYANASENSPLKVIDFGLSVCFKPGARFNEIVGSPYYMAPEVLKRNYGQEIDIWSAGVILYILLCGVPPFWAETDEGIAQAIIRSHIDFQREPWPKVSDNAKDLVRRMLDPNPYTRLTAQQVLEHPWIQNASAAPNIPLGEAVRSRLKQFTVMNKFKKKALLVVAEYLPTEELDAIRELFNMLDTKKKGHLTLEELRKGLQVIGHNIHDTDVDMLMEAADIDGNGILDCKEFVTVSIHLKKIRSDEHLPKVFSFFDKNGSGYIEIEELKEALSPRGDQKSIDDIFLDVDIDKDGKISYEEFELMMSAGMDWRNASRQYSRAVYNTLSRKIFKEVSLKLDHSGPLVAAGK

>OsCPK30

MGLCHGKPSQIPEPEAEEAAAAAGVAVAGAASPGPAAAAAAAAAAKPGTPKQPKFPFYLPSPLPASSYKSSPANSSVASTPARGGLKRPFPPPSPAKHIRALLARRHGSVKPNEAPIPEGGETEVGLDKGFGFSKHFFAKYELGDEVGRGHFGYTCSAKAKKGDHKGHDVAVKVIPKAKMTTAIAIEDVRREVRILSSLTGHSNLVQFYDAFEDEDNVYIVMELCKGGELLDRILARGGKYSEEDAKVVMVQILSVVSFCHLQGVVHRDLKPENFLFTSKDENSALKVIDFGLSDFVKPDERLNDIVGSAYYVAPEVLHRSYGTEADMWSIGVIAYILLCGSRPFWARTESGIFRAVLKAEPSFDEAPWPTLTAEAKDFVKRLLNKDYRKRMTAAQALSHPWIRNSQQVKIPLDMIIYKLMRAYISSSSLRKSALRALAKTLTANQLFYLREQFELLGPNKNGYISLQNLKTALVKNSTDAMKDSRVIDFVNTVCTLQYRKLDFEEFAASAVSVYQMEALETWEQHARRAYELFDKEGNRPIVIEELASELGLGPSVPLHVVLQDWIRHADGKLSFLGFIKLLHGVSSRSIPKA

>OsCPK31

MRGLVLQGRQQSLQGRLRSSSKFCVSRPVAPRPCKACRRAARSGSSSCSRAVAVQVVASLDYIATASDEGVLKLPARTELDPEEIKSVFGYPRNLLDSYYLGRVIGAGSFGVVREGIEVSTGRRFAVKTVSKVPKRGSPTPRYLLKLRAEVEVMQQLGVSLNAVHLHDVFEDDVNVHMVMELCEGGALLERVESGVYSELYISKLVRSILRFIAQCHAKGIIYRDVNPDNFLFLTAEEDSPLKATDFGLSIRHYSHEPKLTSRSGTPAYMAPELVMQCYDEKADLWSVGMLAYQLLTGRFPFWEDVRNETLSDVWKAILSSEIDWNAPELQPLSSAARDLLERLLQRNPVMRPSAAEALEHPWLAQEGAANDMPLKGSVVQRLQRFATYTHLKQVVLRMITEDMRQRGKAPSFNSALQELFAAYDKDKSGTISFEELAEGLRGQGYVVNESEVRQLMEKMDMDHDGNVGGDEFLATLIDWGQVMQEQEWQSYVDQAFNRMDLDGDGFIDLDELLSELPAAYFHEPSSEDERISEAKRMLREADENGDGRISKQEFYNLLRDNVAPDSLSMYDDRLSHNVAAMSV

>OsCRK1

MGLCHGKSAAVLEPTVEEEEEGATRVAEAAAAPAKPASPAPSAAAAAAAPAKPGTPKQHKFPFYLPSPLPASSYKGSPANSSVASTPARGGFKRPFPPPSPAKHIRALLARRHGSVKPNEASIPESGEPGVALDKGFGFSRHFAAKYELGREVGRGHFGYTCAATCKKGELKGDDVAVKVIPKAKMTTAIAIEDVRREVRILSSLAGHSNLVQFYDAYEDEENVYIVMELCKGGELLDRILARGGKYSEEDAKVVMRQILSVASFCHLQGVVHRDLKPENFLFSSKDENSAMKVIDFGLSDFVKPDERLNDIVGSAYYVAPEVLHRSYGTEADMWSIGVIVYILLCGSRPFWARTESGIFRAVLKADPSFEEAPWPTLSAEAKDFVRRLLNKDYRKRMTAAQALCHPWIRGTEEVKLPLDMIIYRLMRAYISSSSLRRAALRALAKTLTTDQIYYLREQFELIGPNKSDLITLQNLKTALMKNSTNAMKDSRVVDFVNTISNIQYRKLDFEEFSAAAISVYQMEGLETWEQHARQAYEFFDKEGNRPIVIDELASELGLG

PSVPLHVVLQDWIRHPDGKLSFLGFMKLLHGVSSRTIPKT

>OsCRK2

MGQCYGKGASGRTADDEGGVVTEHQSPPPANGLPSTPPRQQAQAQAQQVGTPRRRGSKSGSTTPGHQTPGVAWPSPYPSGGASPLPAGVSPSPARSTPRRFFKRPFPPPSPAKHIKATLAKRLGGGKPKEGTIPEEGGVGAGGGGGGAADGAETERPLDKTFGFSKNFGAKYELGKEVGRGHFGHTCSAVVKKGEYKGQTVAVKIIAKAKMTTAISIEDVRREVKILRALSGHNNLVKFYDACEDGLNVYIVMELCEGGELLDRILARGGRYTEEDAKAIVVQILSVVAFCHLQGVVHRDLKPENFLFTTRDENAPMKLIDFGLSDFIRPDERLNDIVGSAYYVAPEVLHRSYSMEADIWSIGVITYILLCGSRPFWARTESGIFRSVLRADPNFDDSPWPTVSAEAKDFVKRFLNKDYRKRMTAVQALTHPWLRDEQRQIPLDILIFRLIKQYLRATPLKRLALKALSKALREDELLYLKLQFKLLEPRDGFVSLDNFRTALTRYLTDAMKESRVLEFLHALEPLAYRRMDFEEFCAAAISPYQLEALERWEEIAGTAFQQFEQEGNRVISVEELAQELNLAPTHYSIVQDWIRKSDGKLNFLGFTKFLHGVTIRGSNTRRH

>OsCRK3

MGGCHAKPLTHEEADGACSPPRERPQPPATPPRGSGAATPAWKTKPWASPFFGFSTPSPSPAHHLFSASSPRRSPAPSAPTTPARRLLRLPFPPPSPAKHIRQALARRHGPPRPPIPEEGGDVEGEGGRGLDKGFGFNKGFAAKYDMGDEVGRGHFGYTCAAKIKKGARKGDAVAVKVIPKAKMTTSIAIEDVRREVKILKALAGHKNLVQFYDAYEDNDNVYIVMELCEGGELLDRILSRGGKYSEDDAKAVLVQILNVVSFCHIQGVVHRDLKPENFLFTSKDENSQLKAIDFGLSDFVKPDERLNDIVGSAYYVAPEVLHRCYSTEADVWSIGVIAYILLCGSRPFWARTESGIFRSVLKADPSYNEAPWPSLTLEAMDFVKRLLCKDPRRRMTAAQALSHPWIRNYNDIKLPLDILIFRLIKAYIRSSSLRKAALRALSKTLTVDELFYLKGQFSLLEPDRNGCITLDNIRMALTREATYAMKESRVQEILVSLSALQYRRMDFQEFCAAAVSVHQLEALDRWEQHARSAYDFFEKDGNRAIVIDELASELGLSPSVPLHVVLQDWIRHTDGKLSFLGFVKLLHGMSSRSLSKMR

>OsCRK4

MGLCHGKPSQIPEPEAEEAAAAAGVAVAGAASPGPAAAAAAAAAAKPGTPKQPKFPFYLPSPLPASSYKSSPANSSVASTPARGGLKRPFPPPSPAKHIRALLARRHGSVKPNEAPIPEGGETEVGLDKGFGFSKHFFAKYELGDEVGRGHFGYTCSAKAKKGDHKGHDVAVKVIPKAKMTTAIAIEDVRREVRILSSLTGHSNLVQFYDAFEDEDNVYIVMELCKGGELLDRILARGGKYSEEDAKVVMVQILSVVSFCHLQGVVHRDLKPENFLFTSKDENSALKVIDFGLSDFVKPDERLNDIVGSAYYVAPEVLHRSYGTEADMWSIGVIAYILLCGSRPFWARTESGIFRAVLKAEPSFDEAPWPTLTAEAKDFVKRLLNKDYRKRMTAAQALSHPWIRNSQQVKIPLDMIIYKLMRAYISSSSLRKSALRALAKTLTANQLFYLREQFELLGPNKNGYISLQNLKTALVKNSTDAMKDSRVIDFVNTVCTLQYRKLDFEEFAASAVSVYQMEALETWEQHARRAYELFDKEGNRPIVIEELASELGLGPSVPLHVVLQDWIRHADGKLSFLGFIKLLHGVSSRSIPKA

>OsCRK5

MGQCYARNVPVDGEGGGGVVTTTTTTTISVSASAAGEEVEVGRGGGGGGGRRSGRPSPAGTPRRRGATPARSSVAGSPWAGSPLPEGIAPSPAPSATTPRRFFRRPFPPPSPAKHIKASLARRLGQRSPATAQAQQAAKPPAEVPIPEHGGGGGAAAGEVERELDKSFGYDRHFAAKYELGKEVGRGHFGHTCLARARKGDMRGQLLAVKVISKAKMTTAISIEDVRREVKILKALSGHSNLVKFYDACEDALNVYIIMELCEGGELLDRILSRGGRYTEEDAKVIVEQILNVVSFCHLQGVVHRDLKPENFLFSTRDDHSPMKIIDFGLSDFIRPDERLNDIVGSAYYVAPEVLHRSYSTEADMWSIGVITYILLCGSRPFWARTESGIFRSVLRADPNFDDAPWSSISPEAKDFVKRLLNKDYRKRMTAAQALSHPWLRDECRPIPLDMLVFKLIKAYLRSTPFKRAALKALSRAITEDELIYIRAQYNLLEPSSTDGRLCIENFRMALLQNSTDAMKESRALDILNALEPLAYRRMDFEEFRAATISPYQLEALSRWEEIAGTAFEYFEQEGNRPITIEELAQEMNLSSAAYSIVRDWIRPADGKLSFLGYTKFLHGLTMRSSNARRHH
