# Supplementary figures and images for: Genome-wide identification and expression analysis of calcium‑dependent protein kinase and its related kinase gene families in melon (Cucumis melo L.)
Source: PLoS One. 2017 Apr 24;12(4):e0176352. doi: 10.1371/journal.pone.0176352 (PMC5402965; doi:10.1371/journal.pone.0176352)

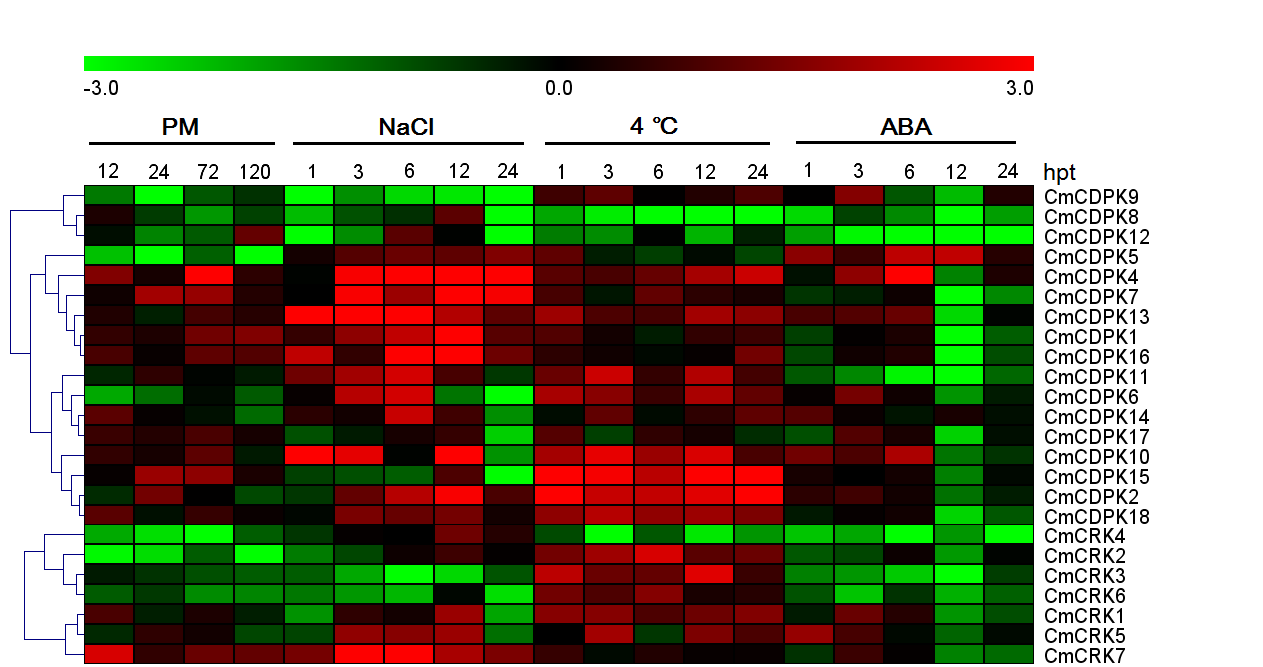

Supplement: S1 Fig — (TIF) [file pone.0176352.s002.tif]
